# Insights into the thermodynamic–kinetic synergistic separation of propyne/propylene in anion pillared cage MOFs with entropy–enthalpy balanced adsorption sites†

**DOI:** 10.1039/d2sc05742e

**Published:** 2022-11-28

**Authors:** Yunjia Jiang, Lingyao Wang, Tongan Yan, Jianbo Hu, Wanqi Sun, Rajamani Krishna, Dongmei Wang, Zonglin Gu, Dahuan Liu, Xili Cui, Huabin Xing, Yuanbin Zhang

**Affiliations:** a Key Laboratory of the Ministry of Education for Advanced Catalysis Materials, College of Chemistry and Life Sciences, Zhejiang Normal University Jinhua 321004 China ybzhang@zjnu.edu.cn; b State Key Laboratory of Organic-Inorganic Composites, Beijing University of Chemical Technology Beijing 100029 China; c Department of Chemistry, Zhejiang University 38 Zheda Road 310027 Hangzhou P. R. China; d Van't Hoff Institute for Molecular Sciences, University of Amsterdam Science Park 904 1098 XH Amsterdam Netherlands; e College of Physical Science and Technology, Yangzhou University Jiangsu 225009 China

## Abstract

Propyne/propylene (C_3_H_4_/C_3_H_6_) separation is an important industrial process yet challenged by the trade-off of selectivity and capacity due to the molecular similarity. Herein, record C_3_H_4_/C_3_H_6_ separation performance is achieved by fine tuning the pore structure in anion pillared MOFs. SIFSIX-Cu-TPA (ZNU-2-Si) displays a benchmark C_3_H_4_ capacity (106/188 cm^3^ g^−1^ at 0.01/1 bar and 298 K), excellent C_3_H_4_/C_3_H_6_ IAST selectivity (14.6–19.3) and kinetic selectivity, and record high C_3_H_4_/C_3_H_6_ (10/90) separation potential (36.2 mol kg^−1^). The practical C_3_H_4_/C_3_H_6_ separation performance is fully demonstrated by breakthroughs under various conditions. 37.8 and 52.9 mol kg^−1^ of polymer grade C_3_H_6_ can be produced from 10/90 and 1/99 C_3_H_4_/C_3_H_6_ mixtures. 4.7 mol kg^−1^ of >99% purity C_3_H_4_ can be recovered by a stepped desorption process. Based on the *in situ* single crystal analysis and DFT calculation, an unprecedented entropy–enthalpy balanced adsorption pathway is discovered. MD simulation further confirmed the thermodynamic–kinetic synergistic separation of C_3_H_4_/C_3_H_6_ in ZNU-2-Si.

## Abstract 2

A chemically robust metal organic framework ZNU-2-Si with optimal pore chemistry and pore structure is reported for record C_3_H_4_ storage and C_3_H_4_/C_3_H_6_ separation by thermodynamic–kinetic synergism along with entropy–enthalpy balanced adsorption sites.

## Graphical abstract

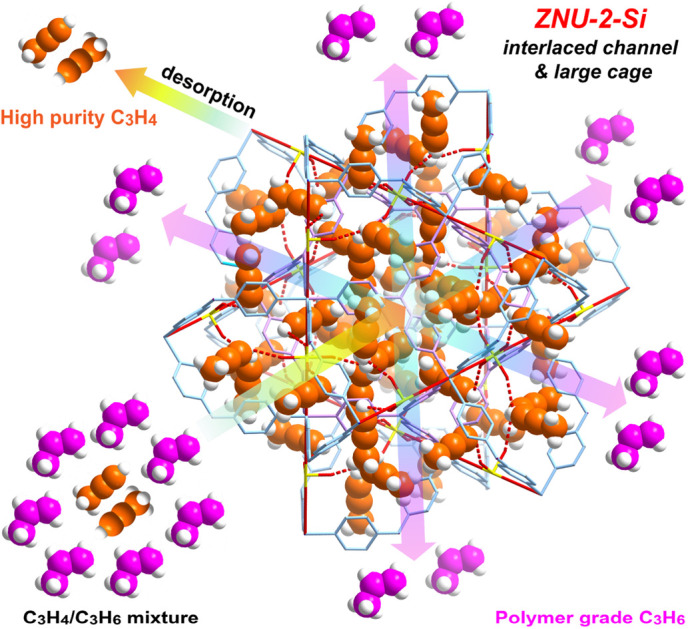

## Introduction

Propylene (C_3_H_6_) is the world's second largest volume hydrocarbon with the global production capacity exceeding 140 million tons in 2020. It is a basic olefin feedstock for the manufacture of various polymers and chemicals such as polypropylene and propylene oxide.^1^ Originating from the cracking of crude oil, C_3_H_6_ is inevitably mixed with a small amount of propyne (C_3_H_4_), which must be reduced to a ppm level before further processing as it can severely poison the C_3_H_6_ polymerization catalysts.^2^ The state-of-the-art industrial technologies for the removal of C_3_H_4_ from C_3_H_6_ rely on noble-metal catalyst based selective hydrogenation, which suffers from several drawbacks such as high cost, low efficiency and potential secondary pollution. On the other hand, C_3_H_4_ that can be manufactured from the catalytic or thermal pyrolysis of C_3_H_6_ is also a fundamental material for speciality fuel and chemical intermediates.^3^ To recover C_3_H_4_, solvent extraction is the current dominant technology, which is not only energy intensive but also associated with pollution. Thus, it is of urgent importance to develop new technologies for efficient C_3_H_4_/C_3_H_6_ separation.

**Scheme 1:**
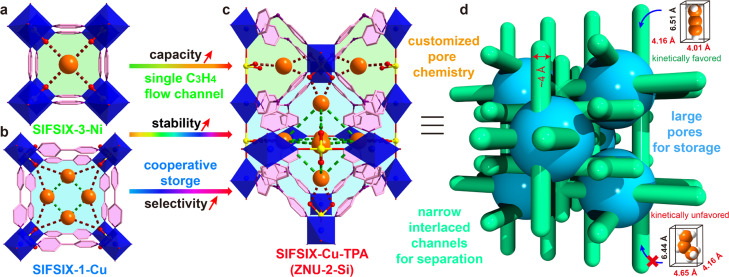
Strategies to overcome the trade-off of capacity and selectivity in C_3_H_4_/C_3_H_6_ separation in a stable cage-like APMOF by the thermodynamic–kinetic synergism mechanism. (a) Structure of SIFSIX-3-Ni. (b) Structure of SIFSIX-1-Cu. (c) Structure of SIFSIX-Cu-TPA. (d) Illustration of the thermodynamic–kinetic cooperation for C_3_H_4_/C_3_H_6_ separation.

Adsorptive separation based on porous solid adsorbents has been recognized as a promising alternative technology for gas/vapor separation because of its eco-friendly nature and energy efficiency.^4–10^ However, due to the great similarity in the molecular size (C_3_H_4_: 4.16 × 4.01 × 6.51 Å^3^, C_3_H_6_: 4.65 × 4.16 × 6.44 Å^3^) and polarizability (C_3_H_4_: 55.5 × 10^−25^ cm^3^, C_3_H_6_: 62.6 × 10^−25^ cm^3^), the adsorptive separation of C_3_H_4_/C_3_H_6_ by molecular recognition is still very challenging.^11^ Only two examples of zeolites are reported and their C_3_H_4_ capacity is relatively low.^10^ Recently, metal–organic frameworks (MOFs) with tuneable pore size/shape and chemistry have emerged as a new class of porous materials for the separation of C_3_H_4_/C_3_H_6_.^12–27^ Among them, **pcu** type anion pillared MOFs (APMOFs) with strong Lewis basic functional sites display the benchmark separation performance.^12–18^ Nonetheless, the trade-off between the capacity and selectivity is still a critical problem to overcome. For example, SIFSIX-3-Ni (pore size = 4.2 Å, Scheme 1a) as a single-molecule trap for C_3_H_4_ can afford extremely high C_3_H_4_/C_3_H_6_ selectivity (>200), but the capacity of C_3_H_4_ is only 67 cm^3^ g^−1^; SIFSIX-1-Cu (pore size = 8.0 Å, Scheme 1b) can accommodate a large amount of C_3_H_4_ (201 cm^3^ g^−1^) by cooperative host–guest interactions, but the separation selectivity is <10. Besides, these pillared layered SIFSIX MOFs are not chemically stable and some are even sensitive to humid air, which hinders the practical applications.^8*d*,28^ On the other hand, the kinetic separation of C_3_H_4_/C_3_H_6_ has never been reported.

Anion pillared cage-like MOFs with **ith-d** topology are a new class of APMOFs.^27,29^ The rational combination of anion pillars and tridentate organic linkers provides anion sustained cage-like APMOFs with ultrahigh chemical stability. In 2021, Wu *et al.* discovered the first pillar-cage **ith-d** MOF SIFSIX-Cu-TPA (Scheme 1c) with a complete SiF_6_^2−^ cross-link for CO_2_/C_2_H_2_ separation.^29^ Soon after, our group reported independently an isomorphic MOF termed ZNU-2 (TIFSIX-Cu-TPA).^27^ Considering the slight change of the organic linker, the metal ion or anion pillar in **pcu**-type APMOFs can lead to a dramatic separation difference, we envision that tuning the pore aperture and pore window in cage-like APMOFs can also be applied to tune the gas adsorption properties. Nonetheless, the length and angle matching between the tridentate ligand and anion pillar is very significant to construct the pillar embedding structures. Pillar-cage Tripp-Cu-SIFSIX with the overlong organic linker 2,4,6-tris(4-pyridyl)pyridine is not stable upon guest removal since the mononuclear Cu ion center is only half sustained by SiF_6_^2−^ and coordination unsaturated.^30^ [Cu_3_(SiF_6_)_3_(TMTPB)_4_] (FJI-W1) with triangular ligand 1,3,5-trimethyl-2,4,6-tris(4-pyridyl)benzene belongs to pillar-layer APMOFs that feature 1D hexagonal channels.^26^ Thus, only the modification of the anion pillar or metal ion (size difference < 0.1 Å) is a good alternative to fine-tune the pore structure and chemistry while retaining the topology. Furthermore, the integration of large cages and narrow interlaced channels has the potential to show a kinetic difference for C_3_H_4_ and C_3_H_6_ molecules with a slight diameter difference (Scheme 1d), which has not been explored in theory yet.

With this in mind, herein we prepared three isomorphic APMOFs using SiF_6_^2−^, TiF_6_^2−^, and NbOF_5_^2−^ as the pillars, and investigated the C_3_H_4_/C_3_H_6_ adsorption and separation performance. To our delight, these three reticular MOFs exhibit quite distinctive but ordered C_3_H_4_ adsorption capacity as well as C_3_H_4_/C_3_H_6_ selectivity. The pore size follows the sequence of SIFSIX-Cu-TPA < TIFSIX-Cu-TPA < NbOFFIVE-Cu-TPA while the C_3_H_4_ adsorption capacity and the separation selectivity are both SIFSIX-Cu-TPA > TIFSIX-Cu-TPA > NbOFFIVE-Cu-TPA. Benchmark high uptakes of C_3_H_4_ are observed both at low pressure (106 STP cm^3^ g^−1^ at 0.01 bar and 298 K) and normal pressure (188 STP cm^3^ g^−1^ at 1 bar and 298 K) on SIFSIX-Cu-TPA. The C_3_H_4_ storage density reached 0.60 and 0.65 g cm^−3^ at 298 and 278 K, 89% and 97% of the liquid C_3_H_4_ density. The calculated IAST selectivity is 14.6–19.3 depending on the ratio of C_3_H_4_/C_3_H_6_ (1/99–50/50). Record high C_3_H_4_/C_3_H_6_ (10/90) separation potential (36.2 mol kg^−1^) is obtained, which is 65% higher than the previous benchmark of NKMOF-11 without anion functionalities. The modestly high C_3_H_4_ adsorption heat of 43.2 kJ mol^−1^ is advantageous for both C_3_H_4_ adsorption and facile regeneration. Simulated breakthroughs indicated SIFSIX-Cu-TPA displays the best separation performance for C_3_H_4_/C_3_H_6_ (10/90) mixtures. Practical separations of C_3_H_4_/C_3_H_6_ (50/50, 10/90, 1/99) mixtures were also confirmed by breakthrough experiments. Notably, the practical separation performance is even superior to that of simulation due to the kinetic enhancement, which has never been reported in C_3_H_4_/C_3_H_6_ separation. 37.8 and 52.9 mol kg^−1^ of C_3_H_6_ is produced from the 10/90 and 1/99 C_3_H_4_/C_3_H_6_ mixtures, respectively. The productivity is increased to 79.2 mol kg^−1^ when the process temperature decreased to 278 K. Such high productivity has never been achieved by chemically stable porous materials. 4.7 mol kg^−1^ of >99% purity C_3_H_4_ can be recovered. Repeated breakthrough experiments under dry or humid conditions showed the complete retention of separation performance, confirming the high stability of SIFSIX-Cu-TPA for practical separations. The *in situ* single crystal structure of C_3_H_4_-loaded SIFSIX-Cu-TPA directly demonstrates the C_3_H_4_ binding configuration under near-saturation conditions, which is distinct from the previous study.^27^ Comprehensive modelling studies including Grand Canonical Monte Carlo (GCMC) simulations, Molecular Dynamics (MD) simulations and Density Functional Theory (DFT) calculations were completely applied to investigate the adsorption/separation process, which indicated that the contracted channel serves as a single molecule flow channel that differentiates C_3_H_4_/C_3_H_6_ kinetically while the large cage provides high affinity for C_3_H_4_ adsorption by cooperative host–guest and guest–guest interactions. To the best of our knowledge, the kinetic separation of C_3_H_4_/C_3_H_6_ has for the first time been revealed by MD simulations. The obvious thermodynamic–kinetic synergism in breakthroughs has never been reported in porous materials for C_3_H_4_/C_3_H_6_ separation. Moreover, our study unprecedentedly disclosed the important role of entropy effects on C_3_H_4_ adsorption and gas cluster assembly in the pores while the GCMC and DFT based gas binding configuration may not reflect the practical gas binding sites due to the neglect of the entropy effect.

## Results and discussion

**Fig. 1:**
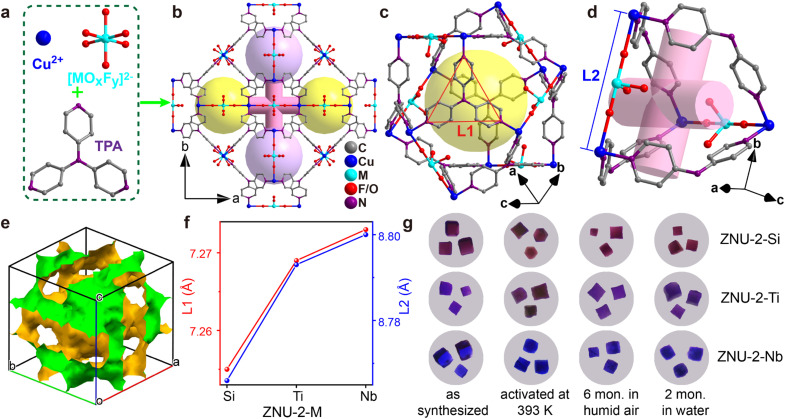
Porous structure and stability test of ZNU-2-M (M = Si, Ti, Nb). (a) Basic units to construct ZNU-2-M. (b) Structure of ZNU-2-M with cage-like pores and interlaced channels. (c) Structure of the icosahedral Cu^II^ cage with the N⋯N distance (L1) of TPA highlighted. (d) Structure of the interlaced channel between four cages with the Cu⋯Cu distance (L2) highlighted. (e) The voids of ZNU-2-Si illustrating the interlinked 3D channels. (f) Comparison of the L1 and L2 among ZNU-2-M. (g) Optical microscopy of single crystals of ZNU-2-M (M = Si, Ti, Nb) after different treatments.

The single crystals of isostructural SIFSIX-Cu-TPA (ZNU-2-Si), TIFSIX-Cu-TPA (ZNU-2-Ti) and NbOFFIVE-Cu-TPA (ZNU-2-Nb) are all produced by layering a MeOH solution of TPA onto an aqueous solution of CuX (X = SiF_6_^2−^, TiF_6_^2−^, NbOF_5_^2−^). ZNU-2-Nb has been reported for the first time (Fig. 1a and b). All of these three coordination complexes crystallize in three-dimensional (3D) frameworks with the cubic *Pm*3̄*n* space group (Table S1†). The frameworks consist of large icosahedral cages (∼8.5 Å, Fig. 1c) with 12 outlets and narrow interlaced channels (∼4 Å, Fig. 1d) that connect four independent cages (Fig. S6 and S7†). The large cages have abundant Lewis basic F binding sites in the surface for C_3_H_4_ adsorption and storage. Such interconnected 3D channel pores (Fig. 1e) are distinct from those of pillar-layer MOFs (*e.g.* SIFSIX-3-Ni) with straight 1D channels. Due to the tiny size difference of anion pillars, the pore aperture and channel diameter also show a very slight difference (<0.05 Å), which is reflected in the N⋯N and Cu⋯Cu distances (Fig. 1f). As the channel diameter is very close to the cross-sections (4.01 × 4.16 Å^2^ for C_3_H_4_ and 4.65 × 4.16 Å^2^ for C_3_H_6_), a slight shrinking of the channel may provide a much enhanced kinetic difference in C_3_H_4_/C_3_H_6_ adsorption. Thus, ZNU-2-Si with a reduced channel diameter has the potential to show a remarkable kinetic difference in C_3_H_4_ and C_3_H_6_ adsorption. In brief, ZNU-2-Si features the most promising structure to offer benchmark C_3_H_4_/C_3_H_6_ separation performance by thermodynamic–kinetic synergism.

Before gas adsorption experiments, the chemical and thermal stability of ZNU-2-M is fully studied since stability is a prerequisite for practical use in real-world systems. To our delight, all three materials are highly stable in humid air and water as indicated by the PXRD patterns (Fig. S12, S14 and S15†). To provide straightforward evidence, we take photographs of the single crystals of ZNU-2-M after different treatments (Fig. 1g, S86, S88 and S90†). As shown, the single crystals are still of high quality after being left in humid air for 6 months, soaking in water for 2 months, soaking in acidic and basic aqueous solutions or exposure to humid HCl vapor at 323 K for 3 h. Heating at 393 K under vacuum provides desolvated ZNU-2-M with the same crystal structure as indicated by single crystal and powder XRD analysis (Fig. 1g, S11, S14, S15 and S86–S90†). TGA curves showed that the framework of ZNU-2-M is stable at 523, 581, and 573 K (250, 308 and 300 °C), respectively (Fig. S13–S15†). The weight reduction between room temperature and 423 K is ascribed to the loss of solvents (MeOH/H_2_O) in the pores of ZNU-2-M.

Encouraged by the ultrahigh stability of ZNU-2-M, we are interested in investigating their permanent porosity as well as unary gas adsorption difference between C_3_H_4_ and C_3_H_6_. At first, N_2_ gas adsorption experiments at 77 K were conducted (Fig. S16–S19†), which indicated their microporous character with pore size distribution in the range of 6.27–9.84 Å, 6.56–9.40 Å, and 7.85–9.40 Å, respectively, very close to the pore aperture of ∼8.5 Å calculated from the single crystal structure. The BET surface areas and pore volumes are 1339/1380/1281 m^2^ g^−1^ and 0.565/0.575/0.521 cm^3^ g^−1^, for ZNU-2-Si, ZNU-2-Ti and ZNU-2-Nb respectively. These BET surface areas are all superior to the benchmark of SIFSIX-1-Cu (1128 m^2^ g^−1^) in pillar-layer APMOFs.^14^

**Fig. 2:**
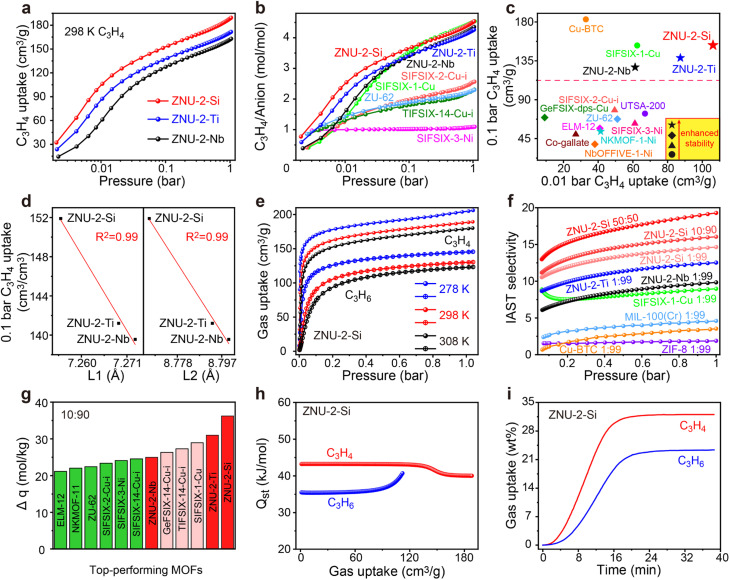
(a) C_3_H_4_ adsorption isotherms in the ZNU-2 family at 298 K. (b) Comparison of the C_3_H_4_ adsorption isotherms of the ZNU-2 family with fluorinated anion hybrid microporous materials. (c) Comparison of the low pressure C_3_H_4_ uptake and stability among top-performing MOFs. (d) Plot of C_3_H_4_ uptake at 0.1 bar *vs.* N⋯N distance (L1)/Cu⋯Cu distance (L2). (e) C_3_H_4_ and C_3_H_6_ adsorption isotherms for ZNU-2-Si at 278, 298 and 308 K. (f) Comparison of IAST selectivity of the ZNU-2 family with other MOFs showing high C_3_H_4_ capacity (>100 cm^3^ g^−1^). (g) Comparison of ZNU-2's IAST based separation potential (Δ*q* = C_3_H_4_ uptake × 9 − C_3_H_6_ uptake) for C_3_H_4_/C_3_H_6_ (10/90) mixtures with reported top performing MOFs. (h) The isosteric heat of adsorption, *Q*_st_, for C_3_H_4_ and C_3_H_6_ in ZNU-2-Si. (i) Adsorption kinetic curves of C_3_H_4_ and C_3_H_6_ in ZNU-2-Si.

Single-component C_3_H_4_ adsorption isotherms were then collected at 298 K (Fig. 2a). At 1 bar, the C_3_H_4_ uptakes were 188, 171 and 162 cm^3^ g^−1^ for ZNU-2-Si, ZNU-2-Ti and ZNU-2-Nb, corresponding to 4.52, 4.25, and 4.34 C_3_H_4_ molecules adsorbed per anion (Fig. 2b). Such a high C_3_H_4_/anion ratio means every free F site can bind 1.13, 1.06, and 1.09 C_3_H_4_ molecules, much higher than those of SIFSIX-2-Cu-i (2.57), TIFSIX-14-Cu-i (2.31), ZU-62 (2.30) and SIFSIX-3-Ni (1.09) (Fig. 2b). The adsorption capacities under 0.01 and 0.1 bar were further compared with those of other MOFs (Fig. 2c). The C_3_H_4_ uptake of ZNU-2-Si at 0.01 bar is record high at 106 cm^3^ g^−1^. This uptake is even much higher than the saturated capacities (1 bar) of most MOFs, such as ELM-12 (61.4 cm^3^ g^−1^),^19^ SIFSIX-3-Ni (66.8 cm^3^ g^−1^),^14^ NKMOF-11 (69.4 cm^3^ g^−1^),^20^ GeFSIX-14-Cu-i (75.3 cm^3^ g^−1^),^17^ Ca-based MOF (67.4 cm^3^ g^−1^),^25^ UTSA-200 (81.1 cm^3^ g^−1^),^15^ ZU-62 (82.0 cm^3^ g^−1^)^18^ and TIFSIX-14-Cu-i (86.5 cm^3^ g^−1^).^17^ Interestingly, a good negative linear relationship between the C_3_H_4_ uptakes under low pressure (0.1 bar) and the N⋯N/Cu⋯Cu distances is observed (Fig. 2d). Such a structure–capacity relationship has never been reported before. Then C_3_H_4_ and C_3_H_6_ adsorption isotherms on ZNU-2-M at 278, 298 and 308 K were all collected (Fig. 2e). The C_3_H_6_ adsorption capacities are much lower than those of C_3_H_4_, especially in low pressure regions. The C_3_H_4_/C_3_H_6_ selectivity on ZNU-2 at 298 K was calculated by using Ideal Adsorbed Solution Theory (IAST). As shown in Fig. 2f, the selectivity of ZNU-2-Si for 1/99 C_3_H_4_/C_3_H_6_ is 14.64, which is higher than that of ZNU-2-Ti (12.53), ZNU-2-Nb (9.84), ZIF-8 (1.9),^15^ FJI-W1 (2.2),^26^ Cu-BTC (3.2),^15^ MIL-100(Cr) (4.5),^15^ and SIFSIX-1-Cu (9.0)^14^ (Fig. 2f). The increased ratio of C_3_H_4_ in the gas mixture results in increased C_3_H_4_/C_3_H_6_ selectivity, which is 16.05 and 19.29 for 10/90 and 50/50C_3_H_4_/C_3_H_6_ mixtures, respectively. The simultaneous increase of the C_3_H_4_/C_3_H_6_ selectivity along the uptakes or C_3_H_4_ ratios suggests the existence of cooperative interactions inside ZNU-2-Si.

The static C_3_H_4_ and C_3_H_6_ uptakes from the 10/90 mixture of C_3_H_4_/C_3_H_6_ were calculated for the ZNU-2 family and other leading materials (Fig. S30, S35, S40 and S42–S48†). The separation potential (Δ*q* = *q*_1_*y*_2_/*y*_1_ − *q*_2_)^31^ as a combined metric of both selectivity and capacity was utilized here for further comparison, which showed a trend of ZNU-2-Si (36.2 mol kg^−1^) > ZNU-2-Ti (31.0 mol kg^−1^) > SIFSIX-1-Cu (29.0 mol kg^−1^)^14^ > TIFSIX-14-Cu-i (27.3 mol kg^−1^)^17^ > GeFSIX-14-Cu-i (26.3 mol kg^−1^)^17^ > ZNU-2-Nb (25.0 mol kg^−1^) > SIFSIX-14-Cu-i (24.6 mol kg^−1^) > SIFSIX-3-Ni (24.1 mol kg^−1^)^14^ > SIFSIX-2-Cu-i (23.4 mol kg^−1^)^14^ > ZU-62 (22.4 mol kg^−1^)^18^ > NKMOF-11 (22.0 mol kg^−1^)^20^ > ELM-12 (21.2 mol kg^−1^)^19^ at 1 bar and 298 K (Fig. 2g). The isosteric enthalpies of adsorption (*Q*_st_) for ZNU-2-M were then calculated with the Clausius–Clapeyron equation. *Q*_st_ values at near-zero loading for C_3_H_4_ and C_3_H_6_ were 43.2/43.0/41.6 and 35.5/34.5/32.4 kJ mol^−1^, respectively (Fig. 2h, S28, S33 and S38†). The *Q*_st_ values for C_3_H_4_ in the ZNU-2 family are lower than those of most MOFs for C_3_H_4_/C_3_H_6_ separation such as ZU-62 (71.0 kJ mol ^−1^),^25^ SIFSIX-3-Ni (68.0 kJ mol^−1^),^14^ NKMOF-1-Ni (65.1 kJ mol ^−1^),^25^ Ca-based MOF (55.4 kJ mol ^−1^),^25^ UTSA-200 (55.3 kJ mol^−1^),^15^ ELM-12 (60.6 kJ mol ^−1^)^19^ and SIFSIX-2-Cu-i (46.0 kJ mol ^−1^),^14^ but slightly higher than that of SIFSIX-1-Cu (37.2 kJ mol^−1^)^14^ (Table S18†). Such modestly high *Q*_st_ not only facilitates preferential C_3_H_4_ adsorption, but also allows the facile recovery of C_3_H_4_ by desorption under mild conditions. To further compare the adsorption difference of C_3_H_4_ and C_3_H_6_ on ZNU-2-Si, we studied the kinetic adsorption behavior. The adsorption rate of C_3_H_4_ in ZNU-2-Si is faster than that of C_3_H_6_. This means that the intra-crystalline diffusion of C_3_H_4_ is faster than that of C_3_H_6_ (Fig. 2i). To the best of our knowledge, such kinetic difference has not been reported in pillar-layered APMOFs for C_3_H_4_/C_3_H_6_ separations. Besides, adsorption thermodynamics and diffusion are usually anti-synergistic as stronger adsorption often implies reduced mobility.^32^ Therefore, ZNU-2-Si with thermodynamic–kinetic synergism is highly promising to provide benchmark practical C_3_H_4_/C_3_H_6_ separation performance.

**Fig. 3:**
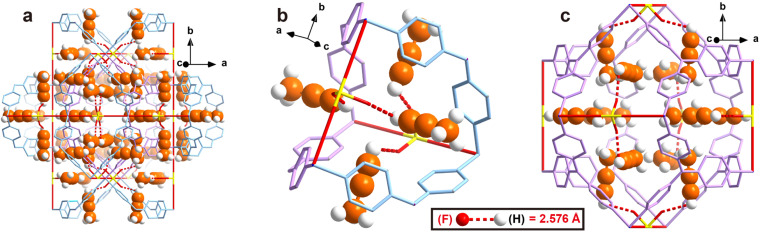
Single crystal structure of C_3_H_4_ loaded ZNU-2-Si. (a) A holistic view. (b) View around the interlaced channel. (c) View around the cage.

To obtain direct host–guest interaction information between ZNU-2-Si and adsorbed gases, we introduced C_3_H_4_ and C_3_H_6_ into the desolvated ZNU-2-Si and measured it in the single crystal X-ray diffractometer. Due to the high stability of ZNU-2-Si, the C_3_H_4_ and C_3_H_6_ loaded structures are ambiguously resolved (Fig. 3 and S10†). 24 C_3_H_4_ molecules and 18 C_3_H_6_ molecules are adsorbed per cell, equal to 4 C_3_H_4_ and 3 C_3_H_6_ molecules for every SIFSIX anion, consistent with the experimental results. After adsorption, the framework remained in the same cubic *Pm*3̄*n* space group with negligible bond or angle changes (Table S2†). From the *in situ* crystals, C_3_H_4_ showed disorder into two overlapping configurations. The configuration with the alkynyl C–H end closer to SiF_6_^2−^ is chosen to be discussed in the following text. The hydrogen bond distances between the terminal C_3_H_4_ hydrogen and F atom of SiF_6_^2−^ are all 2.576 Å. Interestingly, no single C_3_H_4_ molecule is completely loaded in the narrow interlaced single molecule channel, which was previously considered as the best energy favored single molecule binding site.^27^ Instead, four C_3_H_4_ molecules are equally close to the interlaced channel while their alkynyl C–H ends are inside (Fig. 3b). On the other hand, all C_3_H_4_ molecules can be considered to locate in the large cage with their C–H end reaching outside (Fig. 3c). Therefore, every large cage can accommodate 12 C_3_H_4_ molecules. The C_3_H_6_ adsorption sites are very close to that for C_3_H_4_. Due to the high symmetry, every free F atom is able to bind 0.75 C_3_H_6_ molecules (Fig. S10†). Strangely, the hydrogen bond distance (2.108 Å) between terminal =CH_2_ and the F atom is even shorter than the ≡C–H⋯F distance. Thus, DFT calculations are applied to directly compare their binding energies, which will be discussed in the next part.

The structure of ZNU-2-Si with less C_3_H_4_ loading is also obtained by controlling the adsorption pressure at 0.01 bar. However, due to the ultrahigh adsorption uptake of C_3_H_4_ under low pressure, there is still a large amount of C_3_H_4_ observed in the cage of ZNU-2-Si and the binding sites are the same. The only difference is the occupancy of every C_3_H_4_ molecule is only *ca.* 50%, equal to 6 C_3_H_4_ molecules in every cage. Such uptake is close to the experimental adsorption capacity (106 cm^3^ g^−1^) under 0.01 bar.

**Fig. 4:**
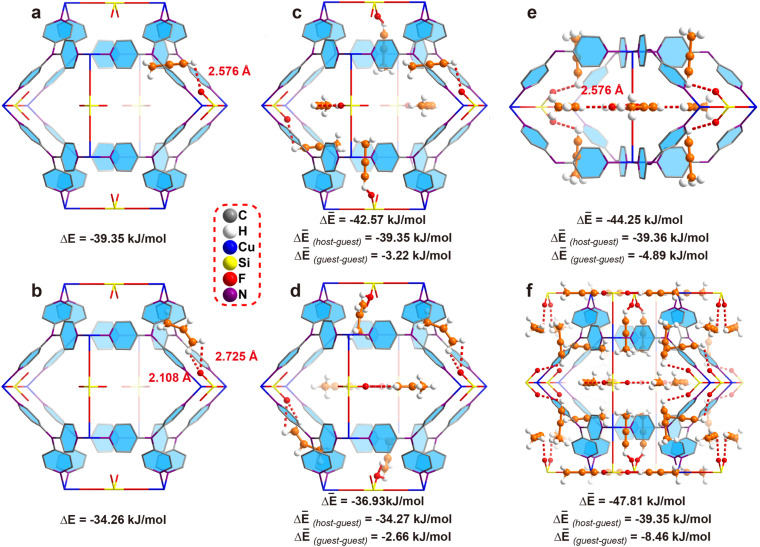
The DFT-D calculated interaction energy of ZNU-2-Si and gas molecules under different loadings based on the single crystal structure. (a and b) A C_3_H_4_ or C_3_H_6_ molecule located in a cage. (c and d) Six C_3_H_4_ or C_3_H_6_ molecules located in a cage. (e) 8 C_3_H_4_ molecules located near two neighbouring interlaced channels. (f) 24 C_3_H_4_ molecules in a unit cell.

In most of the literature, bond length is used to compare the interaction strength. However, in our case, the ≡C–H⋯F distance (2.576 Å) is longer than the =CH⋯F distance (2.108 Å), making it difficult to judge which interaction is stronger as the acidity of the ≡C–H hydrogen is stronger than that of =CH_2_. Thus, crystallography based DFT calculation is applied to calculate the bonding energy. First of all, we calculate the bonding energy between the framework and single gas molecule. To our delight, the results indicated the binding energy between a single C_3_H_4_ molecule and ZNU-2-Si is −39.35 kJ mol^−1^ (Fig. 4a) while that for C_3_H_6_ is only −34.26 kJ mol^−1^ (Fig. 4b), indicating the interaction between C_3_H_4_ and ZNU-2-Si is stronger. The binding energy difference (5.09 kJ mol^−1^) is also close to the experimental *Q*_st_ difference (7.7 kJ mol^−1^).

The binding energies of ZNU-2-Si and six gas molecules were also calculated, which are −42.57 and −36.93 kJ mol^−1^ for C_3_H_4_ and C_3_H_6_ (Fig. 4c and d), respectively. These binding energies can be separated into two parts: ZNU-2-Si⋯gas (host–guest) interaction energy and gas⋯gas (guest–guest) interaction energy. For C_3_H_4_, the ZNU-2-Si⋯C_3_H_4_ binding energy is still −39.35 kJ mol^−1^ and the interaction energy of six C_3_H_4_⋯C_3_H_4_ molecules is −3.22 kJ mol^−1^ (Fig. 4c). For C_3_H_6_, the ZNU-2-Si⋯C_3_H_6_ binding energy is −34.27 kJ mol^−1^ and the interaction energy of six C_3_H_6_⋯C_3_H_6_ molecules is −2.66 kJ mol^−1^ (Fig. 4d). These results indicated that the C_3_H_4_⋯C_3_H_4_ interactions are stronger than C_3_H_6_⋯C_3_H_6_ interactions in the confined cavity while the ZNU-2-Si⋯gas molecules remained nearly unchanged with the loading increase.

To gain more insight into the C_3_H_4_⋯C_3_H_4_ cluster, we choose another two models with different C_3_H_4_ molecules for comparison. Fig. 4e displays the structure of 8 C_3_H_4_ molecules in two neighboring interlaced channels, where the C_3_H_4_⋯C_3_H_4_ interaction energies increased to −4.89 kJ mol^−1^. Fig. 4f displays the complete loading of C_3_H_4_ molecules in the cages (*i.e.* 24 C_3_H_4_ molecules in a unit cell), where the C_3_H_4_⋯C_3_H_4_ interaction energies are further increased to −8.46 kJ mol^−1^. These results unambiguously revealed the boosted C_3_H_4_ adsorption behavior in ZNU-2-Si through cooperative guest⋯guest interactions.

**Fig. 5:**
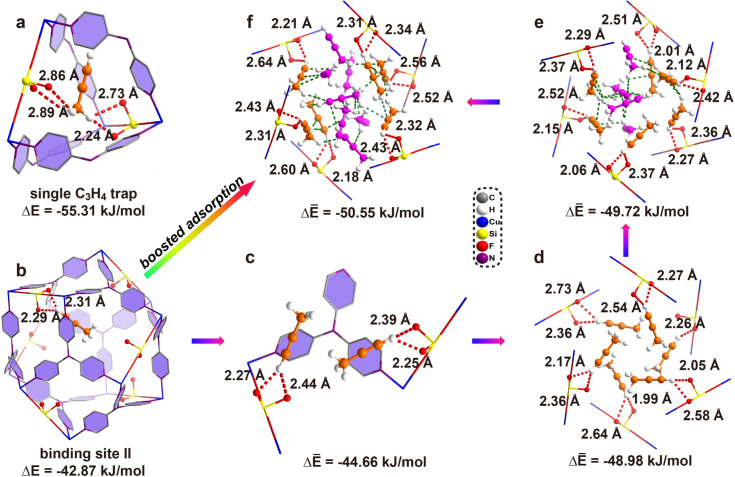
The DFT-D optimized C_3_H_4_ adsorption configuration based on GCMC simulation and bonding energy of C_3_H_4_ in ZNU-2-Si. (a) Binding site I inside the interlaced channel. (b) Binding site II inside the cage. (c–f) 2, 6, 10, and 13 C_3_H_4_ molecules adsorbed inside the cage.

To gain more insight into the gas adsorption behavior, GCMC simulations were performed, which indicated two distinct binding sites: one located completely in the interlaced channel and the other completely inside the cage. Moreover, the results indicated that 30 C_3_H_4_ molecules can be adsorbed in a single unit cell at 298 K and 1 bar (Fig. S68†), equal to 209 cm^3^ g^−1^ for ZNU-2-Si, similar to the experimental value of 188 cm^3^ g^−1^. DFT calculations were then applied to identify the adsorption configuration and binding energies of C_3_H_4_ in ZNU-2-Si. Fig. 5a shows that the C_3_H_4_ molecule in the first binding site is completely in the interlaced channel. The three hydrogen atoms from the methyl group in C_3_H_4_ strongly interact with three F atoms at the sharing edges of four different cages. The hydrogen bond distances are 2.24, 2.73, 2.86 and 2.89 Å. Besides, multiple additional weak van der Waals interactions exist with the C⋯H distances of 2.68, 2.77 and 2.85 Å (Fig. S56†). All of these interactions contribute to a high binding energy of −55.31 kJ mol^−1^. The second binding site located inside the cage adsorbs C_3_H_4_ by two strong hydrogen bonds between the terminal hydrogen of C_3_H_4_ and two adjacent F atoms with distances of 2.29 and 2.31 Å (Fig. 5b). This binding energy is −42.87 kJ mol^−1^. The binding energy for the second C_3_H_4_ molecule inside the cage increases to −46.45 kJ mol^−1^. Thus, the average binding energy of two C_3_H_4_ molecules inside the cavity is −44.66 kJ mol^−1^. In addition, the binding energies increase to −48.98, −49.72, and −50.55 kJ mol^−1^ for accommodation of 6, 10 and 13 C_3_H_4_ molecules in a cage, respectively (Fig. 5c–f). To provide direct comparison, the GCMC simulation result with 24 C_3_H_4_ molecules located both in the narrow channel and the large cage was chosen as a model for optimization. DFT calculation indicated the average bonding energy is −50.85 kJ mol^−1^ (Fig. S69a†), which is still higher than that (−47.81 kJ mol^−1^) based on the single crystal structure. Moreover, the GCMC optimized C_3_H_4_ configurations (*i.e.* 6 C_3_H_4_ molecules completely in the 6 narrow channels and 18 C_3_H_4_ molecules in two large cages) do not display distinct changes under DFT optimization.

As described above, the GCMC based DFT calculation obviously provided more energy favorable binding sites for C_3_H_4_ molecules compared to those based on the single crystal structure. Then why do C_3_H_4_ molecules not follow this pathway for accommodation? Analysis of the cage-channel structure indicates that the narrow interlaced channel is the only passage that connects cages. Gas molecules in cage I must pass through the intersection to reach cage II. Thus, once the intersection is occupied, the diffusion is limited. Moreover, the entropy of C_3_H_4_ in the interlaced channel is the lowest because the rotation is highly restricted in the narrow channel. The diffusion of C_3_H_4_ from the intersection to the large cages is entropy favorable. Therefore, the experimentally observed C_3_H_4_ binding configuration is an entropy–enthalpy balanced result. As GCMC simulations neglect the influence of diffusion or entropy effect, they may not reflect the real binding sites in biporous materials.^33^ Binding site I (Fig. 5a) can be the exact location for the adsorption of the first C_3_H_4_ molecule under extremely low pressure. Once the pressure or number of C_3_H_4_ molecules increases, the diffusion or entropy effect becomes obvious, and the symmetrical binding sites in Fig. 4 to provide higher entropy are favored. On the other hand, the final C_3_H_4_ adsorption configuration can be considered as the result of competitive adsorption of C_3_H_4_ from different cages. Due to the high symmetry of the framework, four C_3_H_4_ molecules in the neighbouring large cages show the same potential to enter the interlaced channel to be strongly trapped but this narrow channel can only accommodate a single C_3_H_4_ completely. Thus, four C_3_H_4_ molecules squeeze their smaller ≡C–H ends into the interlaced channel but leave their larger ≡C–CH_3_ ends outside of the channel. We further calculate the bonding energy between 24 C_3_H_4_ molecules and ZNU-2-Si based on single crystal structures with all molecules relaxed (Fig. S69b†). In this case, the binding energy of −50.42 kJ mol^−1^ is only slightly inferior to the GCMC result (−50.85 kJ mol^−1^), which is easy to be covered by the entropy penalty. In brief, GCMC based DFT calculations can provide some information on the initial adsorption while *in situ* single crystal structures give the direct adsorption behavior under the measured conditions.

**Fig. 6:**
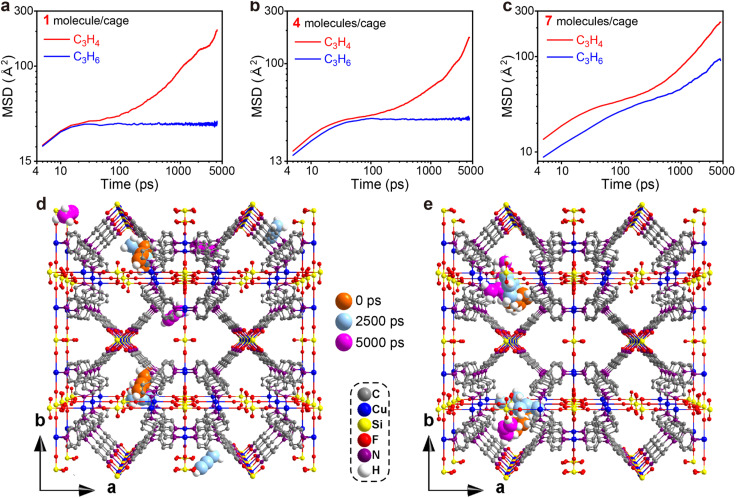
MD simulations. (a–c) MSD plot of C_3_H_4_ and C_3_H_6_ molecules in ZNU-2-Si with 1, 4 and 7 molecules in a single cage. (d and e) Snapshots of MD simulation of C_3_H_4_ (d) and C_3_H_6_ (e) molecules at 0, 2500, and 5000 ps.

To gain some insight into the distinct adsorption kinetic difference of C_3_H_4_ and C_3_H_6_ in ZNU-2-Si as well as to provide more evidence for the entropy effect, MD simulations were carried out. The configurations of C_3_H_4_ and C_3_H_6_ molecules are based on the GCMC simulations and the whole framework is considered flexible except the Cu atoms. Fig. 6a–c illustrate the MSD in the *x*, *y* and *z* directions for 1, 4, and 7 C_3_H_4_ or C_3_H_6_ molecules per cage of ZNU-2-Si respectively. These graphs show that within the period of 5000 ps, the C_3_H_4_ molecules can migrate to other cages through the interlaced channels freely independent of the pressure, namely the number of C_3_H_4_ molecules located in a cage (Fig. 6d, S70, S71 and S73†). However, the C_3_H_6_ molecules can only move inside the original cage and are not able to spread to other cages until the number of molecules accommodated in a single cage reaches 5 (Fig. 6e, S70, S72 and S74†). MD-derived C_3_H_4_ and C_3_H_6_ diffusion coefficients in ZNU-2-Si were further calculated. The values are 4.72 × 10^−11^/6.79 × 10^−14^, 4.89 × 10^−11^/4.64 × 10^−13^, and 7.55 × 10^−11^/2.50 × 10^−11^ m^2^ s^−1^ for 1, 4 and 7 C_3_H_4_ or C_3_H_6_ molecules located inside a cage. Therefore, the diffusion coefficient of C_3_H_4_ is much higher than that of C_3_H_6_, especially under low pressure with the number of the gas molecules in a cage less than 5 (Table S20†). In other words, the diffusion rate of C_3_H_4_ in ZNU-2-Si is much faster than that of C_3_H_6_. The C_3_H_4_/C_3_H_6_ kinetic selectivity is as high as ∼695 under low pressures and ∼3.0 under high pressures. Such high kinetic selectivity has never been found in porous materials for C_3_H_4_/C_3_H_6_ separation, which is highly related to its unique framework structure with large cavities and narrow channels. Since the cages are connected by narrow interlaced channels, the gas molecules must pass through the channels when they need to diffuse from one cage to another. As the cross-section of C_3_H_6_ (4.65 × 4.16 Å^2^) is larger than that of C_3_H_4_ (4.01 × 4.16 Å^2^), larger pressure is needed to expand the channel sizes (original size ∼ 4 Å) by the rotation of the pyridine ring. Therefore, the narrow interlaced channels can be regarded as molecular sieves to allow the C_3_H_4_ molecules to pass through while prohibiting the migration of the C_3_H_6_ molecules under certain pressures. Only when the pressure increased to a higher degree did the gate opening (*i.e.* ligand rotation) allow C_3_H_6_ to diffuse fast within different cages.

We also tried MD simulation considering the framework is completely rigid. In this case, it is very difficult for both C_3_H_4_ and C_3_H_6_ molecules to diffuse from one cage to another due to the limitation of the over-contracted intersection (∼4 Å). Therefore, the free energies of C_3_H_4_ and C_3_H_6_ moving from the narrow channels to the large cages were compared by calculating the potential of mean force (PMF). The results are presented in Fig. S75† which showed that C_3_H_4_ has a lower free energy barrier than C_3_H_6_, suggesting the diffusion of C_3_H_4_ is much easier than C_3_H_6_.

**Fig. 7:**
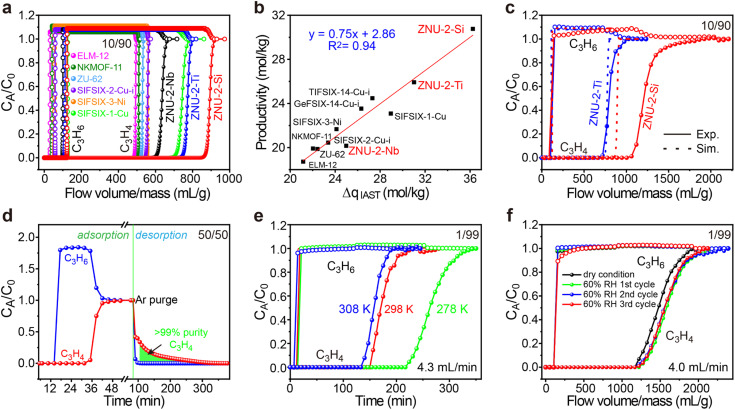
(a) Simulated breakthrough curves of ZNU-2-Si and other top-performing materials for C_3_H_4_/C_3_H_6_ (10/90) at 298 K. (b) Plots of the calculated productivity of C_3_H_6_ in >99.996% purity and separation potential Δ*q*_IAST_. (c) Comparison of the experimental and simulated breakthrough curves of ZNU-2-Si and ZNU-2-Ti for C_3_H_4_/C_3_H_6_ (10/90). (d) Experimental breakthrough curves and desorption curves of ZNU-2-Si for C_3_H_4_/C_3_H_6_ (50/50) at 298 K. (e) Experimental breakthrough curves of ZNU-2-Si for C_3_H_4_/C_3_H_6_ (1/99) at 278, 298, and 308 K. (f) Experimental breakthrough curves of ZNU-2-Si for C_3_H_4_/C_3_H_6_ (1/99) at 298 K under dry and humid conditions (activation conditions of ZNU-2-Si between cycles: Ar flow rate 20 mL min^−1^ at 393 K).

To evaluate the practical separation performance of ZNU-2-Si for selective C_3_H_4_/C_3_H_6_ separation, transient breakthrough simulations were conducted for the 10/90 C_3_H_4_/C_3_H_6_ mixture. The results showed that highly efficient separations could be accomplished by ZNU-2-Si (Fig. 7a). The productivity of C_3_H_6_ (>99.996% purity) in a single adsorption process is also calculated for ZNU-2-Si and other benchmark materials, which showed ZNU-2-Si has the record C_3_H_6_ productivity of 30.8 mol kg^−1^ (Fig. 7b), consistent with the separation potential Δ*q*_IAST_ based on the static gas adsorption isotherms. Experimental breakthrough studies with the C_3_H_4_/C_3_H_6_ (10/90) mixture flowed over a ZNU-2-Si packed column with a flow rate of 4 mL min^−1^ at 298 K were then carried out. The experimental results were superior to the simulated one and 37.8 mol kg^−1^ of high purity C_3_H_6_ can be produced (Fig. 7c). Such enhancement can be attributed to the existence of the kinetic effect, which has never been reported in C_3_H_4_/C_3_H_6_ separation. For isomorphic ZNU-2-Ti, the kinetic enhancement is not obvious. The experimental C_3_H_4_ productivity (25.50 mol kg^−1^) is even slightly lower than that of the simulation (25.93 mol kg^−1^). The difference between ZNU-2-Si and ZNU-2-Ti can be accounted for by the reduced channel aperture in ZNU-2-Si that increases the diffusion difference in C_3_H_4_/C_3_H_6_ adsorption.

To thoroughly identify the separation performance of ZNU-2-Si, we conducted more breakthrough experiments under various conditions. C_3_H_4_/C_3_H_6_ mixtures containing a higher ratio (50%) or lower ratio (1%) of C_3_H_4_ were tested. In both cases, clean C_3_H_4_/C_3_H_6_ separations were achieved. For the 50 : 50 C_3_H_4_/C_3_H_6_ mixture, the retention time of C_3_H_4_ is over twice that of C_3_H_6_. 7.06 mol kg^−1^ of C_3_H_4_ was captured in the column with a purity of ∼86% (Fig. 7d). Controlling the desorption conditions, 4.7 mol kg^−1^ of >99% purity C_3_H_4_ can be recovered from the column by evacuation after blowing C_3_H_6_ out firstly (Fig. 7d and S81†). This record high dynamic productivity of C_3_H_4_ is impossible to obtain by other APMOFs due to their low C_3_H_4_ capacity. For the 1 : 99 C_3_H_4_/C_3_H_6_ mixture, C_3_H_6_ broke out at ∼18 min and became saturated immediately while C_3_H_4_ was not detected until ∼156 min and reached saturation slowly (Fig. 7e). The calculated experimental productivity of C_3_H_6_ from the 1 : 99 C_3_H_4_/C_3_H_6_ mixture at 298 K is 52.9 mol kg^−1^, much higher than those of SIFSIX-1-Cu (5.0 mol kg^−1^), ELM-12 (15.0 mol kg^−1^), SIFSIX-3-Ni (20.0 mol kg^−1^), SIFSIX-2-Cu-i (25.5 mol kg^−1^) and ZNU-2-Ti (42.0 mol kg^−1^). Upon lowering the experimental temperature to 278 K, the productivity of C_3_H_6_ increased to 79.20 mol kg^−1^, exceeding that of UTSA-200 (62.9 mol kg^−1^, 298 K)^15^ and NKMOF-11 (74.4 mol kg^−1^, 298 K)^20^ (Fig. 7e and S79†). Due to its extremely high water stability, we further carried out the breakthrough experiments under humid conditions. The C_3_H_4_/C_3_H_6_ (1 : 99) mixture was firstly bubbled into a bottle full of water and then introduced into the column packed with ZNU-2-Si. The humidity was measured constantly, which was stable at ∼60% after reaching equilibrium. From the repetitive humidity tests, it can be concluded that the influence of moisture is negligible for C_3_H_4_/C_3_H_6_ separation in ZNU-2-Si (Fig. 7f). Finally, the breakthrough experiments were conducted for six cycles, and the excellent separation capacity of ZNU-2-Si was retained, indicating that ZNU-2-Si possesses a high cycling stability (Fig. S84 and S85†). In summary, ZNU-2-Si sets a new record for practical simultaneous C_3_H_6_ purification and C_3_H_4_ recovery/storage by the combination of high productivity of polymer grade C_3_H_6_, large amount recovery of C_3_H_4_, retention of separation performance under humid conditions, outstanding recycling capacity and facile regeneration conditions.

## Conclusions

In conclusion, we reported a chemically stable MOF, ZNU-2-Si, with large three-dimensional pores and narrow interlaced channels for record propyne storage and propyne/propylene separation. Notable features of this work include: (1) benchmark C_3_H_4_ capacity of 106 cm^3^ g^−1^ under a low pressure of 0.01 bar; (2) extremely high C_3_H_4_ storage capacity (188 cm^3^ g^−1^, 298 K) and storage density (0.60/0.65 g cm^−3^ at 298/278 K) at 1.0 bar; (3) record high C_3_H_4_/C_3_H_6_ (10/90) separation potential (36.2 mol kg^−1^); (4) record high experimental C_3_H_6_ productivity (37.81 mol kg^−1^) from 10/90 C_3_H_4_/C_3_H_6_ mixtures; (5) record high >99% purity C_3_H_4_ recovery (4.7 mol kg^−1^) from a 50/50 C_3_H_4_/C_3_H_6_ mixture by a stepped desorption process; (6) benchmark experimental C_3_H_6_ productivity (52.9/79.2 mol kg^−1^ at 298/278 K) from 1/99 C_3_H_4_/C_3_H_6_ mixtures; (7) excellent breakthrough recyclability and performance retention under humid conditions; (8) unprecedented revelation of the adsorption and separation mechanism by *in situ* single crystal analysis and GCMC/MD simulations and DFT calculations. In general, our work not only proposes a strategy of using MOFs with large cages and narrow channels for thermodynamic–kinetic synergistic separation, but also highlights the importance of combining the *in situ* single crystal structure analysis and theoretical studies to investigate the adsorption/separation mechanism. These cage-like APMOFs with optimal pore chemistry and pore structures are supposed to be promising for many other challenging gas separations.

## Data availability

All the data supporting this article have been included in the main text and the ESI.†

## Author contributions

Y. J.: synthesis, characterization, adsorption experiments, draft preparation; L. W.: single crystal structure measurement and analysis, funding; T. Y.: GCMC simulation, DFT calculation, MD simulation; J. H.: GCMC simulation, DFT calculation; W. S.: breakthrough experiments; R. K.: IAST calculation, breakthrough simulation; D. W.: discussion, advice; Z. G.: PMF calculation; D. L.: supervision of the theoretical study; X. C.: supervision of the theoretical study; H. X.: supervision of the theoretical study; Y. Z.: concept, supervision, draft preparation, funding.

## Conflicts of interest

There are no conflicts to declare.

## Supplementary Material

SC-014-D2SC05742E-s001

SC-014-D2SC05742E-s002

SC-014-D2SC05742E-s003
