# Supplementary material for: Insights into the thermodynamic–kinetic synergistic separation of propyne/propylene in anion pillared cage MOFs with entropy–enthalpy balanced adsorption sites
Source: Chem Sci. 2022 Nov 28;14(2):298–309. doi: 10.1039/d2sc05742e (PMC9811657; doi:10.1039/d2sc05742e)
Supplement: SC-014-D2SC05742E-s001 [file SC-014-D2SC05742E-s001.pdf]

Electronic Supplementary Material (ESI) for Chemical Science.
This journal is © The Royal Society of Chemistry 2022

Supporting information

Insights into thermodynamic-kinetic synergistic separation of propyne/propylene in anion pillared cage MOFs with entropy-enthalpy balanced adsorption sites

Yunjia Jiang,[§a] Lingyao Wang,[§a] Tongan Yan,[§b] Jianbo Hu,[§c] Wanqi Sun,[a] Rajamani Krishna,[d] Dongmei Wang,[a] Zonglin Gu,[e] Dahuan Liu,[b] Xili Cui,[c] Huabin Xing[c] and Yuanbin Zhang*[a]

[§] These authors contributed equally to this work

[a] Key Laboratory of the Ministry of Education for Advanced Catalysis Materials, College of Chemistry and Life Sciences, Zhejiang Normal University, Jinhua 321004, China. E-mail: ybzhang@zjnu.edu.cn.

[b] State Key Laboratory of Organic-Inorganic Composites, Beijing University of Chemical Technology, Beijing 100029, China.

[c] Department of Chemistry, Zhejiang University 38 Zheda Road, 310027 Hangzhou, P. R. China.

[d] Van't Hoff Institute for Molecular Sciences, University of Amsterdam, Science Park 904, 1098 XH Amsterdam, Netherlands.

[e] College of Physical Science and Technology, Yangzhou University, Jiangsu, 225009, China.

Table of Contents

I	General Information and Procedures	p. S3–S18
II	Characterization (SCXRD, PXRD, TGA)	p. S19–S30
III	Adsorption data, IAST selectivity and *Q*st	p. S31–S59
IV	Crystallography based DFT calculation	p. S60–S65
V	GCMC simulation based DFT calculation	p. S66–S80
VI	MD simulation	p. S81–S87
VII	Breakthrough simulations and experiments	p. S88–S93
VIII	Stability test	p. S94–S100
IX	References	p. S101–S104

I General Information and Procedures

Unless otherwise noted, all the reactions were performed under air without N_2 or Ar protection. All reagents were used as received without purification unless stated otherwise.

Chemicals: Tri(pyridin-4-yl)amine (TPA, 99%) was purchased from Tensus Biotech Company. 4,4'-Bipyridine (98%) and pyrazine (99%) were purchased from Energy Chemical. 1,2-Di(pyridin-4-yl)ethyne (97%) and 1,2-di(pyridin-4-yl)diazene (98%) wee purchased from Chemsoon. The purity of the organic compound was identified by ^{1}H NMR and $^{13}C\{^{1}H\}$ NMR. $Cu[NO_3]_2\cdot 3H_2O$ (99%), $Ni(BF_4)_2$ (99%)， $(NH_4)_2GeF_6$ (99.99%), $(NH_4)_2SiF_6$ (99.99%) and CuO (99%) were purchased from Energy Chemical. $(NH_4)_2TiF_6$ (98%) was purchased from Alab Chemical. Nb_2O_5 was purchased from Macklin. Aqueous H_2SiF_6 (35%) was purchased from Alfa Aesar. HF ($\geq$ 40%) was purchased from Greagent. C_3H_4 (99.9%), C_3H_6 (99.9%), N_2(99.9999%), He (99.9999%), Ar (99.9999%), C_3H_4/C_3H_6 (50:50), C_3H_4/C_3H_6 (10:90) and C_3H_4/C_3H_6 (1:99) were purchased from Datong Co., Ltd. All other reagents were purchased from Adamas-beta and used without further purification.

Preparation of $CuSiF_6\cdot 4H_2O$: $CuSiF_6\cdot 4H_2O$ was prepared according to the reported literature [1]. CuO (3.015 g, 38 mmol, 1 eq) and H_2SiF_6 (aq, 35%, 15 mL, 1 eq) were added to a 50 mL Teflon lined stainless autoclave. The mixture was heated at 105 ℃ for 24 h. After that the mixture was cooled to room temperature and a clear blue aqueous $CuSiF_6$ solution was obtained with a small amount of unreacted CuO black solid in the bottom. After removing the solid by filtration, the blue aqueous solution was evaporated at 80 °C for more than 5 h in an oil bath, yielding the blue crystalline powder of $CuSiF_6\cdot 4H_2O$ (7.8 g, 73.0% based on CuO).

Caution! Hydrofluoric acid is toxic and corrosive! It must be handled with extreme caution and the appropriate protective gear.

Preparation of $CuNbOF_5·4H_2O$: $CuNbOF_5·xH_2O$ was prepared according to the reported literature [2]. CuO (1.50 g, 18.9 mmol, 2 eq), Nb_2O_5 (2.51 g, 9.45 mmol, 1 eq) and 4.11 mL HF (aq, 40%, 10 eq) were added to a 50 mL Teflon lined stainless autoclave. The mixture was stirred under the room temperature for 2 h and then was heated at 60 °C for 24 h. After that the mixture was cooled to room temperature and a clear blue aqueous $CuNbOF_5$ solution was obtained. After removing the solid by filtration, the blue aqueous solution was evaporated at 80 °C for more than 5 h in an oil bath, yielding the blue crystalline powder of $CuNbOF_5·4H_2O$ (5.2 g, 81.0% based on CuO).

Preparation of ZNU-2-Nb: To a 5 mL long thin tube was added a 1 mL of aqueous solution with $CuNbOF_5·4H_2O$ (~1.8 mg). 2 mL of MeOH/H_2O mixture (v:v=1:1) was slowly layered above the solution, followed by a 1 mL of MeOH solution of TPA (~1.0 mg). The tube was sealed and left undisturbed at 298 K. After ~1 week, purple single crystals were obtained.

Preparation of ZNU-2-Ti: To a 5 mL long thin tube was added a 1 mL of aqueous solution with $Cu(NO_3)_2·3H_2O$ (~1.3 mg) and $(NH_4)_2TiF_6$ (~1.0 mg). 2 mL of MeOH/H_2O mixture (v:v=1:1) was slowly layered above the solution, followed by a 1 mL of MeOH solution of TPA (~1.0 mg). The tube was sealed and left undisturbed at 298 K. After ~1 week, purple single crystals were obtained.

Preparation of ZNU-2-Si: To a 5 mL long thin tube was added a 1 mL of aqueous solution with $CuSiF_6·4H_2O$ (~1.5 mg). 2 mL of MeOH/H_2O mixture (v:v=1:1) was slowly layered above the solution, followed by a 1 mL of MeOH solution of TPA (~1.0 mg). The tube was sealed and left undisturbed at 298 K. After ~1 week, dark violet single crystals were obtained.

Preparation of gas loaded ZNU-2-Si: The synthesized ZNU-6 was filled into a glass tube and activated at 120 °C for 12 h. After the sample cooling down, the C_3H_4 or

C_3H_6 was induced into the sample respectively with Builder SSA 7000 (Beijing) instrument until the pressure reach to 1 bar at 298 K and maintain the state for another hour. Then, the tube was sealed. Finally, the crystals were picked out and covered with the degassed oil, and single crystal X-ray diffraction measurements were carried out at 298 K as soon as possible.

Preparation of SIFSIX-1-Cu: SIFSIX-1-Cu was prepared according to the reported literature [3-5]. 58.3 mg 4,4'-bipyridine (0.37 mmol) was dissolved in 6.5 mL ethylene glycol at 338 K in a 25 mL round bottom flask and an aqueous solution (3 mL) of $CuSiF_6 \cdot 4H_2O$ (51.8 mg, 0.19 mmol) was added to the former solution. The mixture was then heated at 338K for 3 h with stirring. The obtained purple powder was washed with methanol, and soaked in anhydrous MeOH for storage.

Preparation of of SIFSIX-2-Cu-i: SIFSIX-2-Cu-i was prepared according to the reported literature [4-6]. A MeOH solution (4.0 mL) of 1,2-di(pyridin-4-yl)ethyne (~51.5 mg, 0.286 mmol) was mixed with an aqueous solution (4.0 mL) of $CuSiF_6 \cdot 4H_2O$ (~72.2 mg, 0.260 mmol) in a 25 mL round bottom flask and then heated at 358 K for 12 h. The obtained blue powder was washed with methanol, and soaked in anhydrous MeOH for storage. Single crystals of SIFSIX-2-Cu-i was prepared according to the reported literature [6]: To a 5 mL long thin tube was added 2 mL of DMSO solution of 1,2-di(pyridin-4-yl)ethyne (20.7 mg). 2 mL of MeOH solution of $CuSiF_6 \cdot 4H_2O$ (41.4 mg) was slowly layered above the solution. The tube was sealed and left undisturbed at 298 K. After ~1 week, blue single crystals were obtained.

Preparation of ZU-62: ZU-62 was prepared according to the reported literature [2]. A preheated water solution (4.0 mL) of $CuNbOF_5$ (~73.0 mg) was mixed with a preheated methanol solution (4.0 mL) of 1,2-di(pyridin-4-yl)ethyne (~51.5 mg) in a 25 mL round bottom flask. Then the mixture was heated at 353 K for 24 h. The obtained blue powder was washed with methanol, and soaked in anhydrous MeOH for storage. Single crystals of ZU-62 were prepared according to the reported literature

[2]: To a long thin tube was added 3 mL of DMSO solution with 1,2-di(pyridin-4-yl)ethyne (~21.9 mg). 2 mL of DMSO/MeOH mixture (v:v=1:1) was slowly layered above the solution, followed by 3 mL of MeOH solution of $CuNbOF_5$ (~15 mg). The tube was sealed and left undisturbed at 298 K. After ~1 week, blue single crystals were obtained.

Prparation of SIFSIX-3-Ni: SIFSIX-3-Ni was prepared according to the reported literature [4, 5, 7]. A methanol solution (20 mL) of $(NH_4)_2SiF_6$ (1 mmol)，$Ni(BF_4)_2$ (1 mmol) and pyrazine (2 mmol) was mixed in a 50 mL round bottom flask, and then heated at 358 K for 3 days. The obtained blue powder was washed with methanol/water, and soaked in anhydrous MeOH for storage.

Preparation of SIFSIX-14-Cu-i: SIFSIX-14-Cu-i was prepared according to the reported literature [8, 9]. A methanol solution (3.0 mL) of 1,2-di(pyridin-4-yl) diazene (~49.0 mg) was mixed with an aqueous solution (2.5 mL) of $CuSiF_6$ (~68.6 mg) in a 25 mL round bottom flask. Then the mixture was heated at 353 K for 15 min, additional 1 h at 323 K, and then at 298 K for 24 h resulting in a bright grey precipitate, which was then washed with methanol, and soaked in anhydrous MeOH for storage. Single crystals of SIFSIX-14-Cu-i were prepared according to the reported literature [8]: Saffron prism-shaped single crystals of SIFSIX-14-Cu-i/UTSA-200 were synthesized in quantitative yield at room temperature by slow diffusion of a methanol solution of $CuSiF_6$ (2 mL, 0.15 mmol) into a DMSO solution of 1,2-di(pyridin-4-yl)diazene (0.12 mmol) after one week.

Preparation of TIFSIX-14-Cu-i: TIFSIX-14-Cu-i was prepared according to the reported literature [10]. A preheated ethanol solution (2.0 mL) of 1,2-di(pyridin-4-yl)diazene (~60.0 mg) was mixed with a preheated glycol solution (3.0 mL) of $Cu(NO_3)_2{\cdot}3H_2O$ (~60.4 mg) and $(NH_4)_2TiF_6$ (~49.5 mg) in a 25 mL round bottom flask. Then the mixture was heated at 338 K for 24 h. The obtained brownish red powder was washed with methanol, and soaked in anhydrous MeOH for storage.

Single crystals of TIFSIX-14-Cu-i were prepared according to the reported literature [10]: To a long thin tube was added 3 mL of DMSO solution with 1,2-di(pyridin-4-yl)diazene (~9 mg). 1 mL of DMSO/ MeOH mixture (v:v=1:1) was slowly layered above the solution, followed by 3 mL of MeOH solution of $Cu(NO_3)_2{\cdot}3H_2O$ (~9.1 mg) and $(NH_4)_2TiF_6$ (~7.5 mg). The tube was sealed and left undisturbed at 298 K. After ~1 week, blue single crystals were obtained.

Preparation of GeFSIX-14-Cu-i: GeFSIX-14-Cu-i was prepared according to the reported literature [11]. A methanol solution (20.0 mL) of 1,2-di(pyridin-4-yl)diazene (~50.3 mg) was mixed with an aqueous solution (25.0 mL) of $Cu(NO_3)_2{\cdot}3H_2O$ (~62.8 mg) and $(NH_4)_2GeF_6$ (~57.9 mg) in a 100 mL round bottom flask. Then the mixture was heated at 298 K for 24 h. The obtained brownish red powder was washed with methanol, and soaked in anhydrous MeOH for storage. Single crystals of GeFSIX-14-Cu-i were prepared according to the reported literature [11]: To a long thin tube was added 3 mL of DMSO solution with 1,2-di(pyridin-4-yl)diazene (~9 mg). 1 mL of DMSO/ MeOH mixture (v:v=1:1) was slowly layered above the solution, followed by 3 mL of MeOH solution of $Cu(NO_3)_2{\cdot}3H_2O$ (~9.1 mg) and $(NH_4)_2GeF_6$ (~8.4 mg). The tube was sealed and left undisturbed at 298 K. After ~2 week, blue single crystals were obtained.

[1] Sun, W. Q. et al. Flexible molecular sieving of C_2H_2 from CO_2 by a new cost-effective metal organic framework with intrinsic hydrogen bonds. *Chem. Eng. J.* **439**, 135745 (2022).

[2] Yang, L. et al. An Asymmetric Anion-Pillared Metal-Organic Framework as a Multisite Adsorbent Enables Simultaneous Removal of Propyne and Propadiene from Propylene. *Angew. Chem. Int. Ed.* **57**, 13145-13149 (2018)..

[3] Noro, S. et al. Framework Engineering by Anions and Porous Functionalities of Cu(II)/4,4'-bpy Coordination Polymers. *J. Am. Chem. Soc.* **124**, 2568-2583 (2002).

[4] Cui, X. et al. Pore chemistry and size control in hybrid porous materials for acetylene capture from ethylene. *Science* **353**, 141-144 (2016).

[5] Yang, L. et al. A Single-Molecule Propyne Trap: Highly Efficient Removal of Propyne from Propylene with Anion-Pillared Ultramicroporous Materials. *Adv. Mater.* **30**, 1705374 (2018).

[6] Nugent, P. et al. Porous materials with optimal adsorption thermodynamics and kinetics for CO_2 separation. *Nature* **495**, 80-84 (2013).

[7] Kumar, A. et al. Direct Air Capture of CO_2 by Physisorbent Materials. *Angew. Chem. Int. Ed.* **54**, 14372-14377 (2015).

[8] Li, B. et al. An Ideal Molecular Sieve for Acetylene Removal from Ethylene with Record Selectivity and Productivity. *Adv. Mater.* **29**, 1704210 (2017).

[9] Li, L. et al. A Metal-Organic Framework with Suitable Pore Size and Specific Functional Sites for the Removal of Trace Propyne from Propylene. *Angew. Chem. Int. Ed.* **57**, 15183-15188 (2018).

[10] Yang, L. et al. A highly sensitive flexible metal–organic framework sets a new benchmark for separating propyne from propylene. *J. Mater. Chem. A* **6**, 24452-24458 (2018).

[11] Zhang, Z. et al. Sorting of C_4 Olefins with Interpenetrated Hybrid Ultramicroporous Materials by Combining Molecular Recognition and Size-Sieving. *Angew. Chem. Int. Ed.* **56**, 16282-16287 (2017).

Single-crystal X-ray diffraction studies were conducted at 293 K、173 K、184 K on the Bruker D8 VENTURE diffractometer equipped with a PHOTON-II detector (MoKα, λ = 0.71073 Å). Indexing was performed using APEX2. Data integration and reduction were completed using SaintPlus 6.01. Absorption correction was performed by the multi-scan method implemented in SADABS. The space group was determined using XPREP implemented in APEX2. The structure was solved with SHELXS-97 (direct methods) and refined on F2 (nonlinear least-squares method) with SHELXL-97 contained in APEX2, WinGX v1.70.01, and OLEX2 v1.1.5 program packages. All non-hydrogen atoms were refined anisotropically. The contribution of disordered solvent molecules was treated as diffuse using the Squeeze routine implemented in Platon.

Powder X-ray diffraction (PXRD) data were collected on the SHIMADZU XRD-6000 diffractometer (Cu Kαλ = 1.540598 Å) with an operating power of 40 KV, 30 mA and a scan speed of 4.0°/min. The range of 2θ was from 5° to 50°.

Thermal gravimetric analysis was performed on the TGA STA449F5 instrument. Experiments were carried out using a platinum pan under nitrogen atmosphere which conducted by a flow rate of 60 mL/min nitrogen gas. First, the samples were heated at 353 K for 2 h to remove the water residue and equilibrated for 5 minutes, then cooled down to 323 K. The data were collected at the temperature range of 323 K to 873 K with a ramp of 10 K /min.

The static gas adsorption equilibrium measurements were performed on the Builder SSA 7000 (Beijing) instrument. Before gas adsorption measurements, the samples of ZNU-2 series (ZNU-2-Nb, ZNU-2-Ti, ZNU-2-Si) (~100 mg) were evacuated at 298 K for 2 h firstly, and then at 393 K for 10 h until the pressure dropped below 7 μmHg. The sorption isotherms were collected at 77, 278, 298 and 308 K on activated samples.The experimental temperatures were controlled by liquid

nitrogen bath (77 K), ethanol-water bath (273 K) and water bath (298 and 308 K), respectively.

Before gas adsorption measurements, the sample of SIFSIX-1-Cu was evacuated at 298 K for 24-48 h until the pressure dropped below 7 μmHg; the sample of SIFSIX-2-Cu-i was evacuated at 353 K for 2 days until the pressure dropped below 7 μmHg; the sample of ZU-62 was evacuated at 353 K for 2 days until the pressure dropped below 7 μmHg; the sample of SIFSIX-3-Ni was evacuated at 353 K for 2 days until the pressure dropped below 7 μmHg; the sample of SIFSIX-14-Cu-i was evacuated at 298 K for 36 h until the pressure dropped below 7 μmHg; the sample of TIFSIX-14-Cu-i was evacuated at 338 K for 24 h until the pressure dropped below 7 μmHg; the sample of GeFSIX-14-Cu-i was evacuated at 298 K for 18 h until the pressure dropped below 7 μmHg. The sorption isotherms were collected at 298 K on activated samples.

The gas adsorption kinetics measurements were performed on the TGA STA449 F5 instrument. Before gas adsorption measurements, the sample of ZNU-2 was activated. After loading the activated ZNU-2-Si (~10 mg) into the pan of the balance (precision: 10^{-7} g), it was firstly heated under N_2 flow (20 mL/min) from 298-423 K with a ramp of 10 K /min. The temperature of 423 K was stayed for 2 hour for the complete removal of moisture adsorbed during the transfer and weighing, which is evidenced by the consistent weight. Then, the sample was cooled to 298 K under N_2 flow (20 mL/min). The temperature of 298 K was stayed for 1 hours. Finally, C_3H_4 or C_3H_6 was introduced with a flow rate of 10 mL/min. The weight was measured constantly.

Fitting of experimental data on pure component isotherms

The unary isotherm data for C_3H_4, and C_3H_6, measured at three different temperatures 278 K, 298 K, and 308 K in ZNU-2 series were fitted with good accuracy using the dual-site Langmuir-Freundlich model, where we distinguish two distinct adsorption sites A and B:

$$q = \frac{q_{sat,A} b_A p^{vA}}{1 + b_A p^{vA}} + \frac{q_{sat,B} b_B p^{vB}}{1 + b_B p^{vB}} \tag{S1}$$

Here, P is the pressure of the bulk gas at equilibrium with the adsorbed phase (Pa), q is the adsorbed amount per mass of adsorbent (mol kg^{-1}), $q_{sat,\ A}$ an $q_{sat,\ B}$ are the saturation capacities of site A and B (mol kg^{-1}), b_A and b_B are the affinity coefficients of site A and B (Pa^{-1}).

In eq (S1), the Langmuir-Freundlich parameters b_A, b_B can be temperature dependent or temperature independent .

$$b_A = b_{A0} \exp\left(\frac{E_A}{RT}\right); \quad b_b = b_{B0} \exp\left(\frac{E_B}{RT}\right) \tag{S2}$$

In eq (S2), E_A, E_B are the energy parameters associated with sites A, and B, respectively.

The isosteric heat of adsorption, Q_{st}, is defined as

$$Q_{st} = -RT^2 \left(\frac{\partial \ln p}{\partial T}\right)_q \tag{S3}$$

where the derivative in the right member of eq (S3) is determined at constant adsorbate loading, q. The calculations are based on the use of the Clausius-Clapeyron equation.

IAST calculations of adsorption selectivity and uptake capacities:

We consider the separation of binary 50/50 C_3H_4(1)/C_3H_6(2), 10/90 C_3H_4(1)/C_3H_6(2) and 1/99 C_3H_4(1)/C_3H_6(2) mixtures in various MOFs at 298 K, and varying total pressures.

The adsorption selectivity for separation of binary mixtures of species 1 and 2 is defined by

$$S_{ads} = \frac{q_1/q_2}{p_1/p_2} \tag{S4}$$

where q_1, q_2 are the molar loadings (units: mol kg^{-1}) in the adsorbed phase in equilibrium with a gas mixture with partial pressures p_1, p_2 in the bulk gas.

The C_3H_4(1)/C_3H_6(2) mixture separations are envisaged to be carried out in fixed bed adsorbers. In such devices, the separations are dictated by a combination of adsorption selectivity and uptake capacity. Using the shock wave model for fixed bed adsorbers, Krishna[1, 2] has suggested that the appropriate metric is the separation potential, Δq_2. The appropriate expression describing the productivity of pure C_3H_6 in the desorption phase of fixed-bed operations is

$$\Delta q_2 = q_1 \frac{y_{20}}{y_{10}} - q_2 \quad \text{(S5)}$$

In eq (S5) y_{10}, y_{20} are the mole fractions of the feed mixture during the adsorption cycle. In the derivation of eq (S5), it is assumed that the concentration "fronts" traversed the column in the form of shock waves during the desorption cycle. The Adsorbed Solution Theory (IAST) of Myers and Prausnitz using the unary isotherm fits as data inputs.[3] The physical significance of Δq_1 is the maximum productivity of pure C_3H_6(2) that is achievable in PSA operations.

Transient breakthrough simulations

The performance of industrial fixed bed adsorbers is dictated by a combination of adsorption selectivity and uptake capacity. Transient breakthrough simulations were carried out for 10/90 and 1/99 C_3H_4(1)/C_3H_6(2) mixtures operating at a total pressure of 100 kPa and 298 K, using the methodology described in earlier publications.[2] In these simulations, intra-crystalline diffusion influences are ignored.

For comparing the separation performance of MOFs, we carried out simulations of transient desorption in which we choose: length of packed bed, L = 0.3 m; superficial gas velocity at the entrance to the bed, $u_0 = 0.04$ m s^{-1}; voidage of the packed bed, ε = 0.4. We choose the mass of the adsorbent in the bed $m_{ads} = 180$ kg,

cross-sectional area, A = 1 m^2; superficial gas velocity at the bed inlet, u_0 = 0.04 m s^{-1}; voidage of the packed bed, = 0.4. The interstitial gas velocity $v = \frac{u}{\varepsilon}$. If the total length of the bed is L m, the total volume of the bed is $V_{bed} = LA$. The volume of zeolite or MOF used in the simulations is $V_{ads} = LA(1-\varepsilon)$. It is important to note that the volume of adsorbent, V_{ads}, includes the pore volume of the adsorbent material. If ρ is the framework density, the mass of the adsorbent in the bed is $m_{ads} = (1-\varepsilon) \times (L \text{ m}) \times (A \text{ m}^2) \times (\rho \text{ kg m}^{-3})$ kg.

For presenting the breakthrough simulation results, we may use the dimensionless time, $\tau = \frac{tu}{L\varepsilon}$, obtained by dividing the actual time, t, by the characteristic time, $\frac{L}{v} = \frac{\varepsilon L}{u_0}$, where L is the length of adsorber, v is the interstitial gas velocity.

For comparison of breakthrough simulations with breakthrough experiments, it is most convenient to use $\frac{Q_0 t}{m_{ads}}$ as the x-axis when presenting the breakthrough simulation data

$$\frac{\left(Q_0 = \text{flow rate mL min}^{-1} \text{ at STP}\right) \times \left(\text{time in min}\right)}{\left(\text{g MOF packed in tube}\right)} = \frac{Q_0 t}{m_{ads}} = \text{mL g}^{-1} \tag{S6}$$

[1] Krishna, R. Screening Metal-Organic Frameworks for Mixture Separations in Fixed-Bed Adsorbers using a Combined Selectivity/Capacity Metric. *RSC Adv*. **7**, 35724−35737 (2017).

[2] Krishna, R. Metrics for Evaluation and Screening of Metal-Organic Frameworks for Applications in Mixture Separations. *ACS Omega* **5**, 16987-17004 (2020).

[3] Myers, A. L. Prausnitz, J. M. Thermodynamics of Mixed Gas Adsorption. *A.I.Ch.E.J.* **11**, 121-130 (1965).

Breakthrough experiments

The breakthrough experiments were carried out in the dynamic gas breakthrough equipment HP-MC41. The experiments were conducted using a stainless steel column (4.6 mm inner diameter × 50 mm length). The weight of ZNU-2-Si and ZNU-2-Ti packed in the columns was 0.49 g and 0.51 g respectively. The column packed with sample was first purged with a Ar flow (5 mL min^{-1}) for 18 h at 393 K. The mixed gas of C_3H_4/C_3H_6 (v/v, 50:50, 10:90, 1:99) was then introduced. Outlet gas from the column was monitored using gas chromatography (GC-9860-5CNJ) with the thermal conductivity detector TCD. After the breakthrough experiment, the sample was regenerated with a Ar purge or under vaccum. All the flowrates are calibrated using self-made soap film flowmeter.

The illustration of the gas breakthrough equipment working mechanism is showing as below: A-B) under work; C) under purge; D) under vacuum.

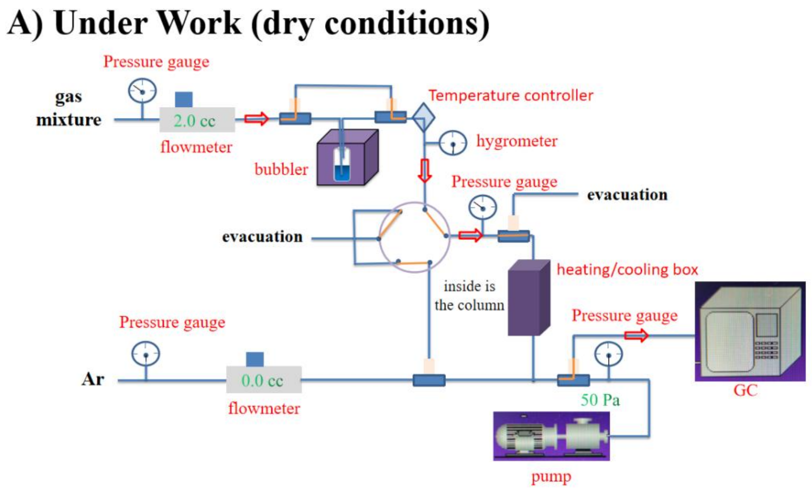

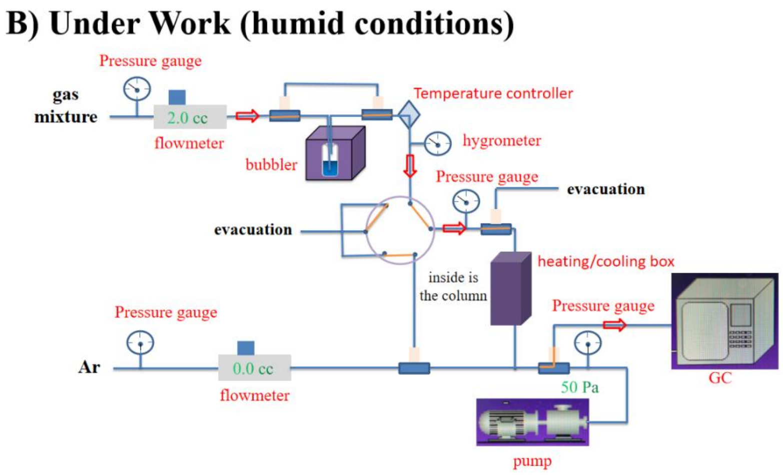

C) Under Purge

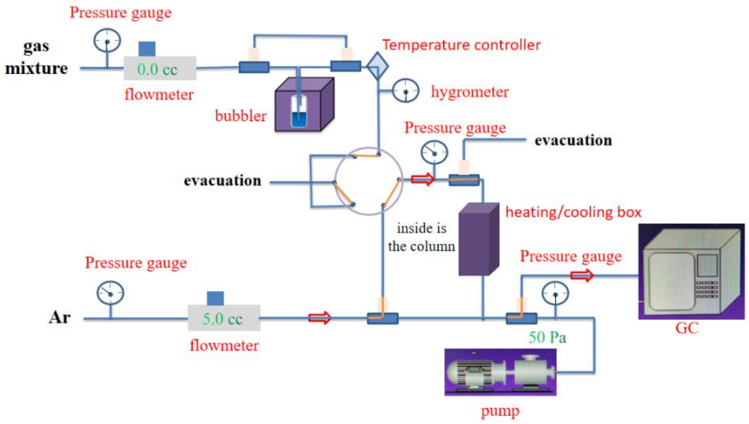

D) Under Vacuum

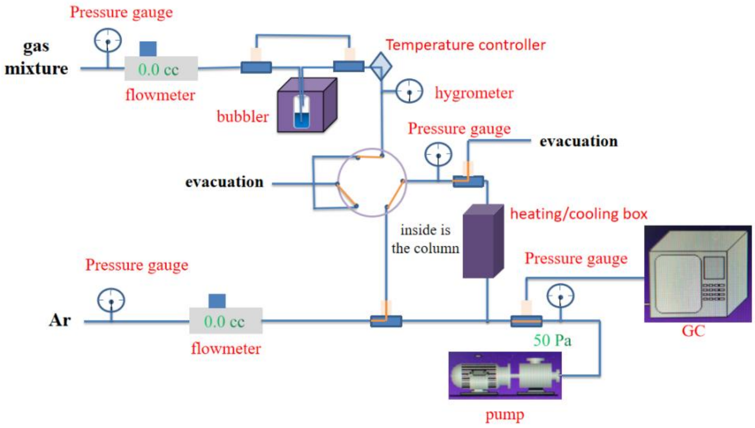

Fig. S1 The illustration of the gas breakthrough equipment working mechanism containing gas pipelines, pressure gauge, flowmeter, hygrometer, GC, bubbler and pump: A) under work in dry conditions; B) under work in humid conditions; C) under purge; D) under vacuum.

Calculation of separation factor (α)

The amount of gas adsorbed i (q_i) is calculated from the breakthrough curve using the following:

$$q_i = \frac{V_T P_i \Delta T}{m}$$

Here, V_T is the total flow rate of gas (cm^3/min), P_i is the partial pressure of gas i (atm), ΔT is the time for initial breakthrough of gas i to occur (mins) and m is the mass of the sorbent (g). The separation factor (α) of the breakthrough experiment is determined as

$$\alpha = \frac{q_1 y_2}{q_2 y_1}$$

Where, y_i is the partial pressure of gas i in the gas mixture.

Molecular simulation

The structures of ZNU-2-Si and ZNU-2-Ti were firstly optimized *via* DFT geometry optimization. The atoms on the framework are assumed to be frozen in their crystallographic positions and the partial point charges of the framework are distributed by QEq method (*Mol. Phys.* **1996**, *87,* 1117–1157). Based on DFT calculations, the ESP charge of the atoms in C_3H_4/C_3H_6 molecules has been calculated. For the framework, the LJ parameters are taken from the UFF force field (*J. Am. Chem. Soc.* **1992**, *114*, 10024–10035). And the LJ parameters for C_3H_4 and C_3H_6 molecules were taken from the optimized OPLS-AA force field by Rego *et. al* (*Fluid Phase Equilibria*, **2022**, *554*, 113314).

Grand canonical Monte Carlo (GCMC) simulations consider four different types of trials: translation, rotation, regrowth, and swap of a molecule adopted the locate task, Metropolis method. During the simulation, the framework was considered to be rigid during the simulations and the interaction energy between the adsorbed molecules and the framework were computed through the Coulomb and Lennard-Jones 12-6 (LJ) potentials. The number of MOF units in the simulation box was 2×2×2 to ensure that the simulation unit was extended, and periodic boundary condition was applied. The cutoff radius was chosen 15.5 Å for Van der Walls interaction and the long-range electrostatic interactions were handled using the Ewald summation method. GCMC simulations of 2×10^7 steps were performed to simulate the favorable adsorption sites and adsorption uptakes at a fixed pressure, with the first 1×10^7 steps used for equilibration and the remaining steps for production. Fugacity was calculated *via* Peng-Robinson equation.

Molecular dynamics (MD) simulations of the canonical ensemble were carried out at 298 K according to the molecular loading results obtained by GCMC simulations,. Each MD was simulated for 5×10^6 steps (i.e., 5 ns) in a time step of 1 fs and then balanced for 5×10^6 steps (i.e., 5 ns). Nosé–Hoover chain (*Mol. Phys.* **1996**, *87,* 1117–1157) thermostat was used to maintain constant temperature conditions, and velocity Verlet algorithm was used to integrate Newton's equation of motion. The framework except Cu atoms is considered flexible during MD simulations. The self-diffusion coefficient can be obtained by averaging 10 independent trajectories. In order to ensure the statistical accuracy of MD simulation, the simulation box was expanded for ZNU-2-Si to increase the number of C_3H_4 and C_3H_6 molecules. From the slope of the mean square displacement (MSD) of the C_3H_4 and C_3H_6, the self-diffusion coefficient of the molecules were calculated. All simulations were performed using Sorption/Forcite module in the Material Studio software version 2017R2.

Potential of mean forces (PMFs) calculation

The PMF values of C_3H_4 and C_3H_6 along the direction from the narrow channel interior to cage-like pores were calculated according to the umbrella sampling approach in Gromacs software package (Figure S74). The framework is considered completely rigid during PMF calculation. The pulling distances (d) to the binding site in channel interior were restrained at a reference distance (d_0) with a harmonic force, $F = k \times (d - d_0)$, where k was the force constant. The spacing of the sampling windows was 0.05 nm. At each d_0, the system was equilibrated for 2 ns, followed by a 10 ns productive run. The free energy profiles were acquired by the g_wham tool that implements the Weighted Histogram Analysis Method.

Density functional theory (DFT) calculations

The static binding energy was calculated using the combination of first principle DFT and plane-wave ultrasoft pseudopotential implemented by CASTEP module in the Materials Studio software version 2017R2. For van der Waals interactions, a semi-empirical addition of dispersive forces was included in the calculation. Calculations were performed under the generalized gradient approximation (GGA) with Perdew–Burke–Ernzerhof (PBE) exchange correlation. A cutoff energy of 520 eV and a 2×2×2 k-point mesh were found to be enough for the total energy to converge within 1×10^{-5} eV $atom^{-1}$. ZNU-2-Si/ZNU-2-Ti crystal structure and an isolated gas molecule in a super cell (with the same cell dimensions as the ZNU-2-Si/ZNU-2-Ti single crystal) were optimized and relaxed. Various guest gas molecules were then introduced to different locations of the channel pore, followed by a full structural relaxation. The equation for the calculation of binding energy (ΔE) is defined as: $\Delta E = E(MOF) + E(gas) - E(MOF+gas)$.

II Characterization (SCXRD, PXRD, TGA)

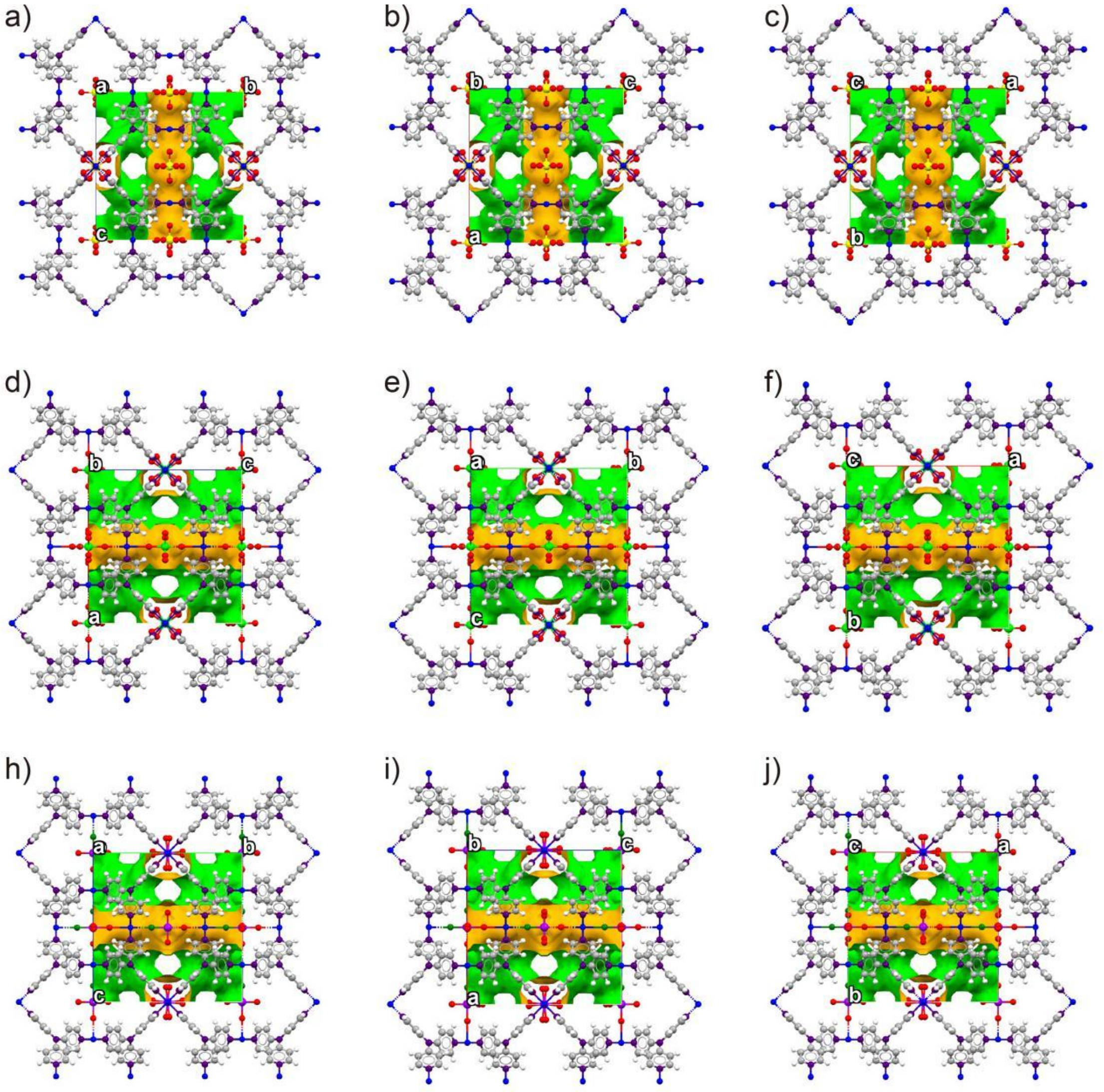

Fig. S2 1 × 1 × 1 packing diagrams of ZNU-2 family viewed down the crystallographic *a*-, *b*-, *c*-axis (a, b, c) in ball-stick mode with pore surface in green representing the inside and yellow the outside determined using a probe with a radius of 1.2 Å by PLATON, (ZNU-2-Si: a-c; ZNU-2-Ti: d-f; ZNU-2-Nb: h-i).

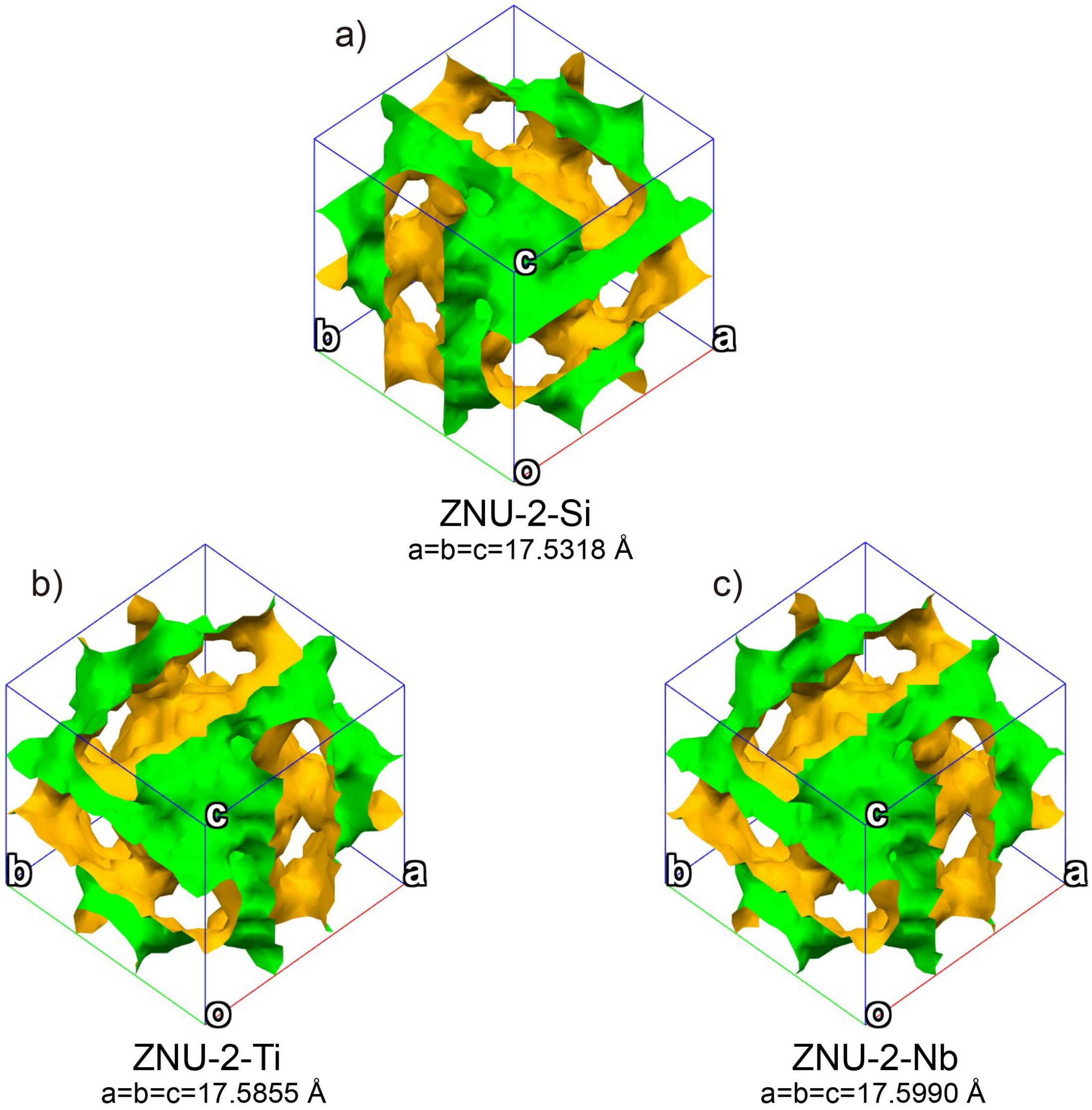

Fig. S3 Void surface of ZNU-2 family (Outside colour: yellow; Inside colour: green).

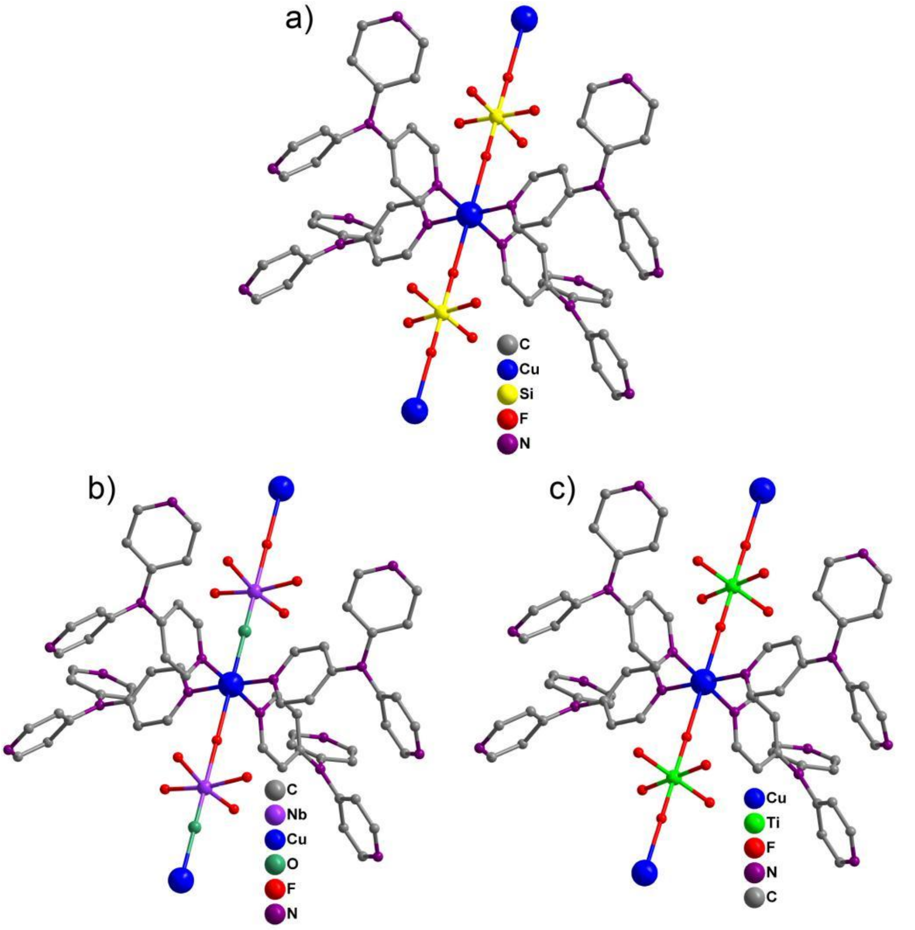

Fig. S4 The Cu(II) coordination environment of the ZNU-2 family (a: ZNU-2-Si; b; ZNU-2-Nb; c: ZNU-2-Ti).

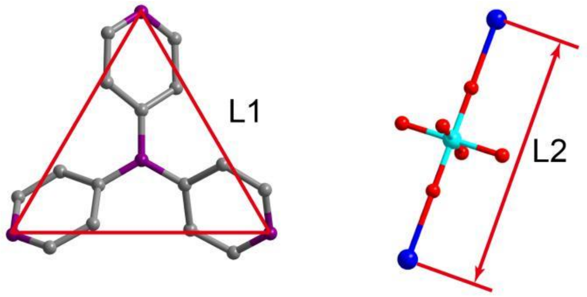

	ZNU-2-Si	ZNU-2-Ti	ZNU-2-Nb
L1 (Å)	7.255	7.269	7.273
L2 (Å)	8.766	8.793	8.800

Fig. S5 The dimensions of tridentate ligands and Cu-Cu distances of the ZNU-2 family .

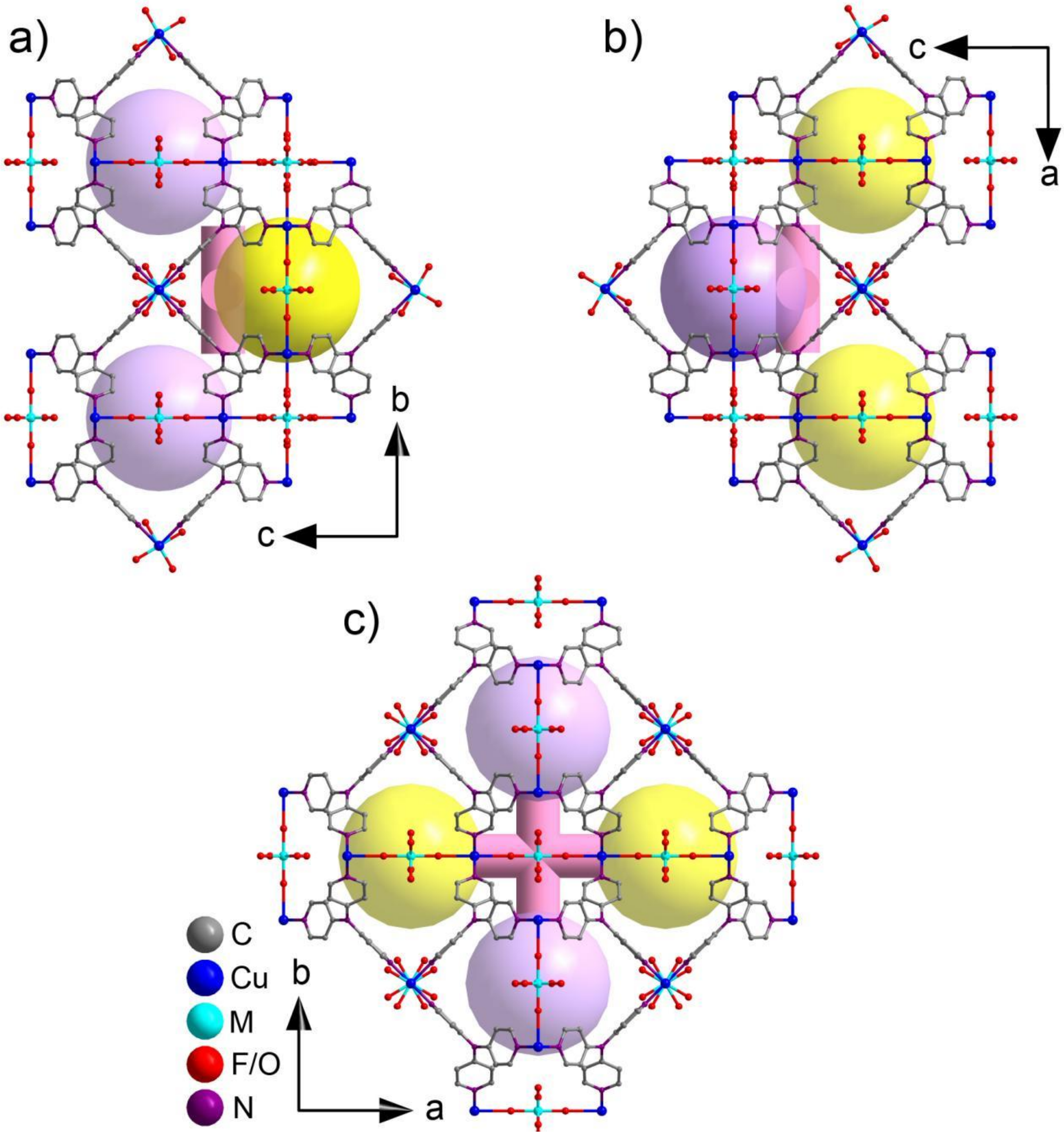

Fig. S6 Structure of the ZNU-2 family with cage-like pores and interlaced channel. One interlaced channel connects four cage-like pores.

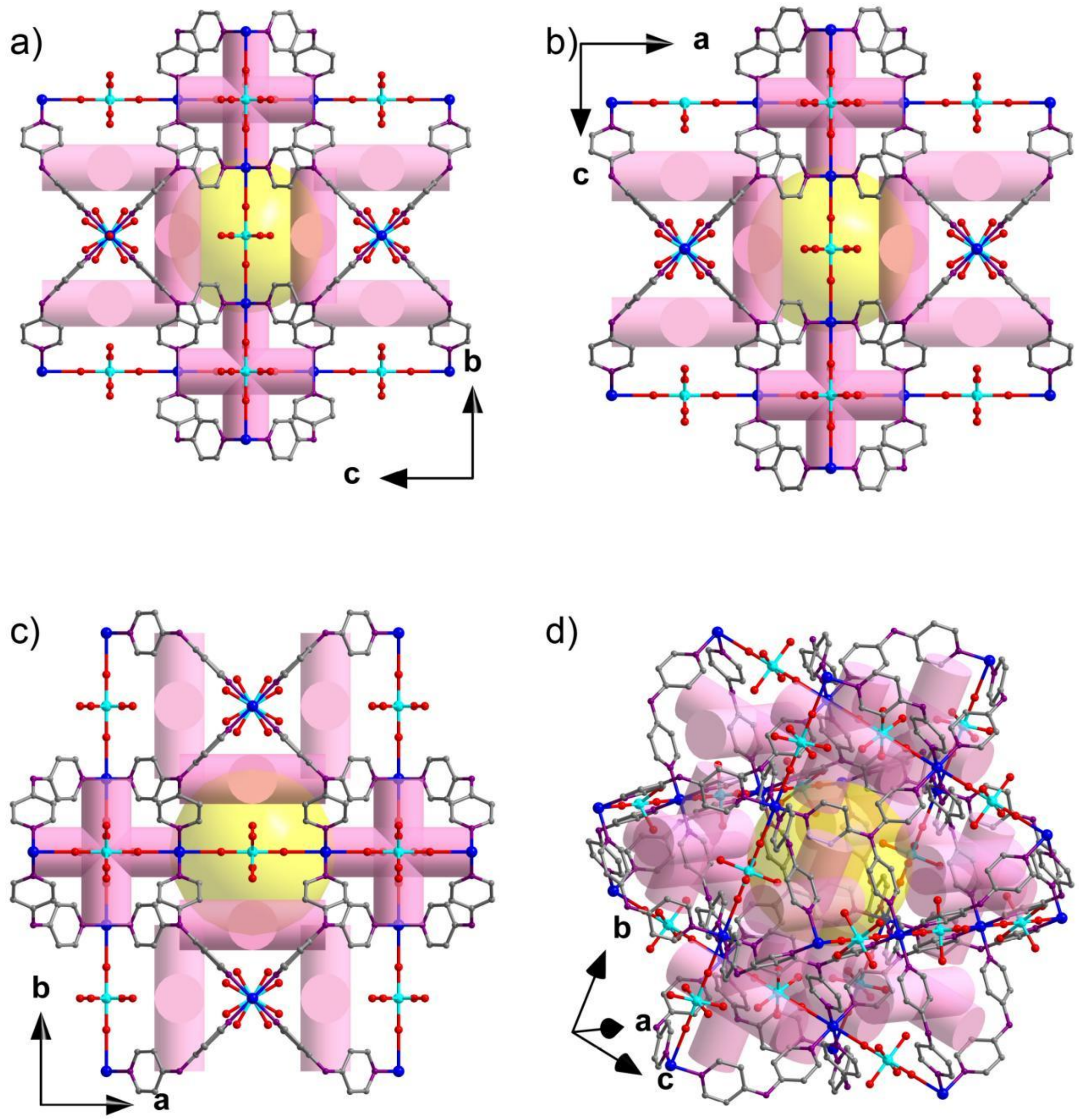

Fig. S7 Structure of the ZNU-2 family with cage-like pore and interlaced channels. One cage-like pore is surrounded with twelve interlaced channels.

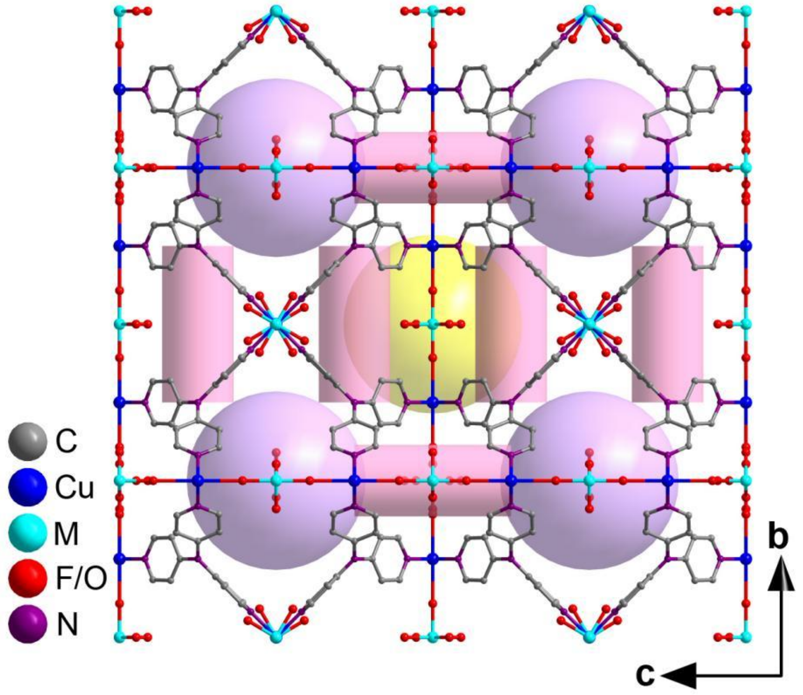

Fig. S8 Structure of the ZNU-2 family viewed from a axis..

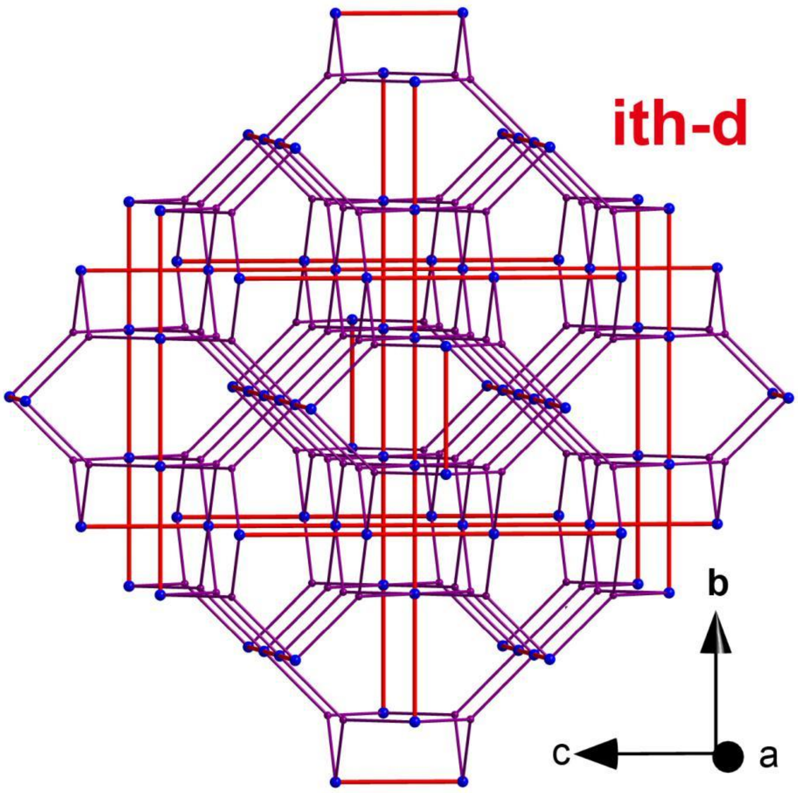

Fig. S9 The ith-d topology of the ZNU-2 family .

Table S1 Crystallographic parameters of the ZNU-2 family .

Materials	ZNU-2-Si	ZNU-2-Ti	ZNU-2-Nb
Cell	a=17.5318(3)	a=17.5855(9)	a=17.5990(5)
	b=17.5318(3)	b=17.5855(9)	b=17.5990(5)
	c=17.5318(3)	c=17.5855(9)	c=17.5990(5)
	α=90	α=90	α=90
	β=90	β=90	β=90
	γ=90	γ=90	γ=90
Temperature	293 K	173 K	184 K
Volume (Å^3)	5388.6(3)	5438.3(8)	5450.8(5)
Space group	Pm-3n	Pm-3n	Pm-3n
Hall group	-P 4n 2 3	-P 4n 2 3	-P 4n 2 3
formula	$C_{20}H_{16}CuF_6N_{5.33}Si$	$C_{20}H_{16}CuF_6N_{5.33}Ti$	$C_{60}H_{48}Cu_3F_{15}N_{16}Nb_3O_3$
MW	536.69	556.49	1795.46
density	0.992	1.020	1.094
Z	6	6	2
R	0.0530(887)	0.0586(989)	0.0487(797)
wR2	0.1813(1142)	0.1890(1137)	0.1458(924)
S	1.133	1.148	1.101
CCDC Nos.	2190368	2142633	2190367/2190959

Table S2 Crystallographic parameters of ZNU-2-Si.

Materials	ZNU-2-Si (as synthesized)	ZNU-2-Si (after activation)	ZNU-2-Si 24 C_3H_4 @ MOF	ZNU-2-Si 18 C_3H_6 @ MOF
Cell	a=17.5318(3)	a=17.5051(3)	a=17.5254(5)	a=17.4998(5)
	b=17.5318(3)	b=17.5051(3)	b=17.5254(5)	b=17.4998(5)
	c=17.5318(3)	c=17.5051(3)	c=17.5254(5)	c=17.4998(5)
	α=90	α=90	α=90	α=90
	β=90	β=90	β=90	β=90
	γ=90	γ=90	γ=90	γ=90
Temperature	293 K	102 K	100 K	101 K
Volume (Å^3)	5388.6(3)	5364.1(3)	5382.8(5)	5359.2(5)
Space group	Pm-3n	Pm-3n	Pm-3n	Pm-3n
Hall group	-P 4n 2 3	-P 4n 2 3	-P 4n 2 3	-P 4n 2 3
formula	$C_{20}H_{16}CuF_6N_{5.33}Si$	$C_{20}H_{16}CuF_6N_{5.33}Si$	$C_{32}H_{32}CuF_6N_{5.33}Si$	$C_{29}H_{34}CuF_6N_{5.33}Si$
MW	536.69	536.69	696.94	662.92
density	0.992	0.997	1.290	1.232
Z	6	6	6	6
R	0.0530 (887)	0.0362 (937)	0.0509 (874)	0.0926 (916)
wR2	0.1813 (1142)	0.1174 (1135)	0.1966 (915)	0.3134 (1135)
S	1.133	1.240	1.176	1.106
CCDC. Nos	2190368	2190370	2190371	2190372

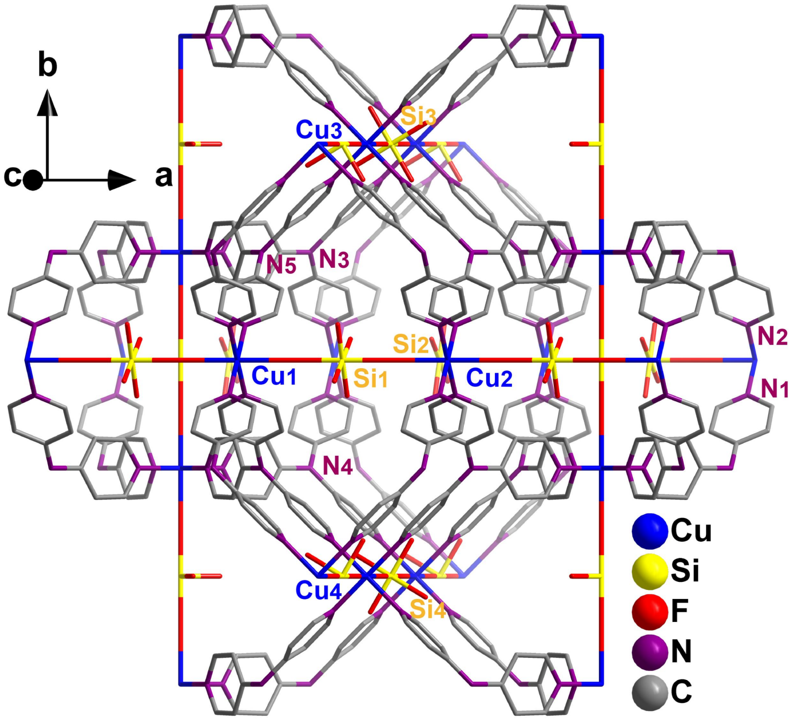

Table S3 Selected bond length in the structures of ZNU-2-Si.

	ZNU-2-Si (as synthesized)	ZNU-2-Si (after activation)	ZNU-2-Si C_3H_4 @ MOF	ZNU-2-Si C_3H_6 @ MOF
Cu1···Cu2	8.766	8.753	8.763	8.750
Cu3···Cu4	17.532	17.505	17.525	17.500
N1···N2	2.842	2.826	2.832	2.821
N3···N4	8.766	8.753	8.763	8.750
N3···N5	8.766	8.753	8.763	8.750
Si1···Si2	17.532	17.505	17.525	17.500
Si3···Si4	17.532	17.505	17.525	17.500

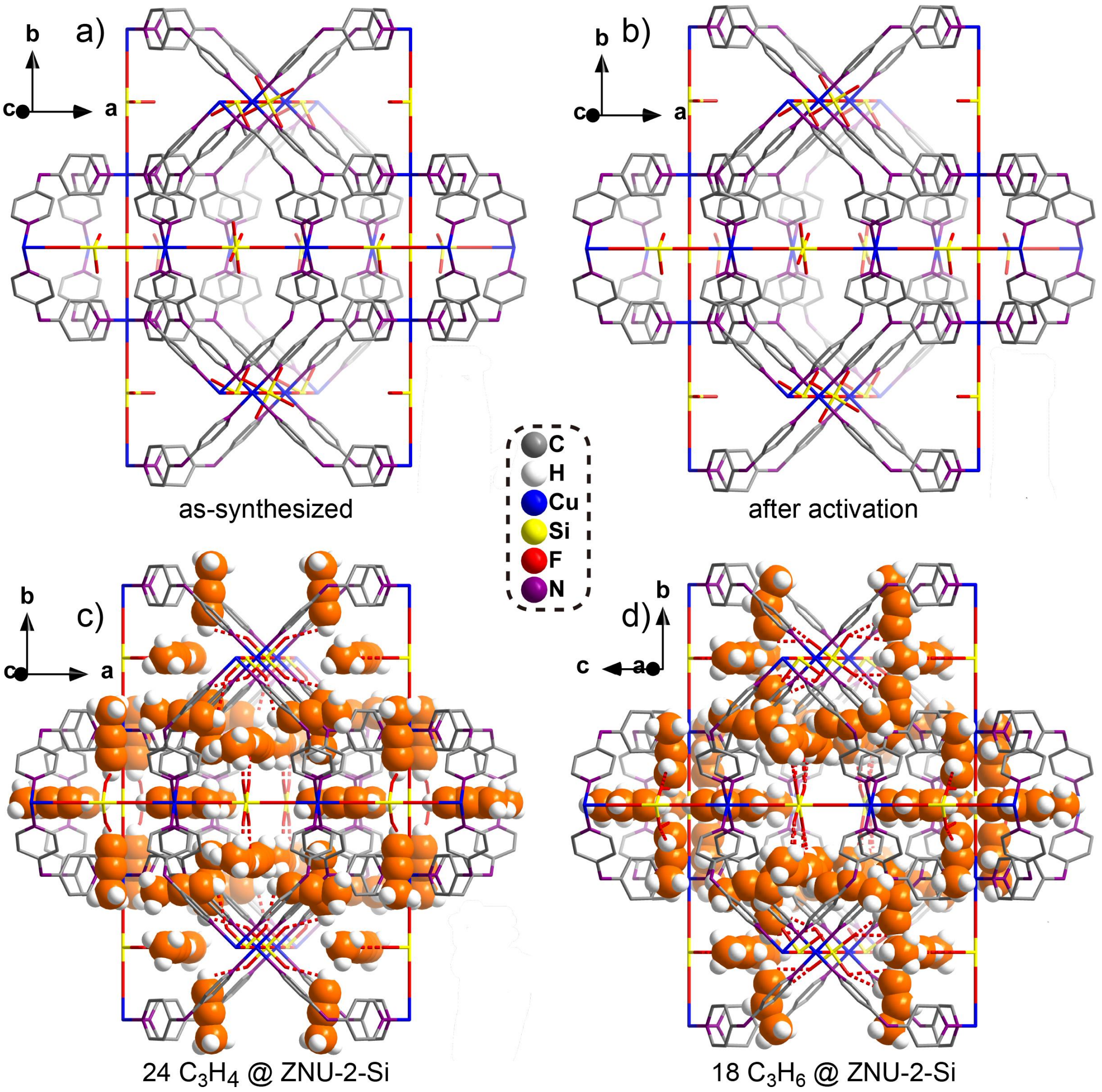

Fig. S10 Single crystal structure of (a) as-synthesized ZNU-2-Si. (b) activated ZNU-2-Si. (c) C_3H_4 loaded ZNU-2-Si. (d) C_3H_6 loaded ZNU-2-Si.

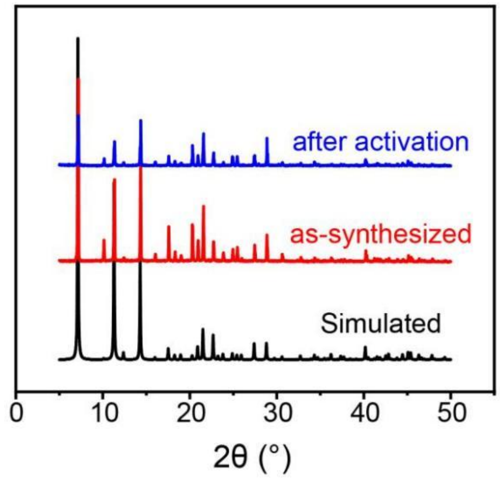

Fig. S11 PXRD patterns of ZNU-2-Si

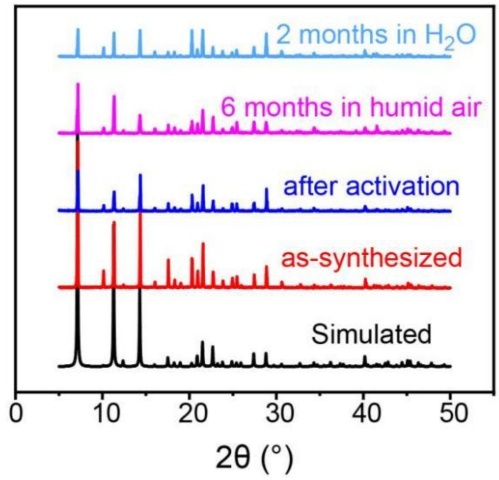

Fig. S12 PXRD patterns of ZNU-2-Si after different treatments.

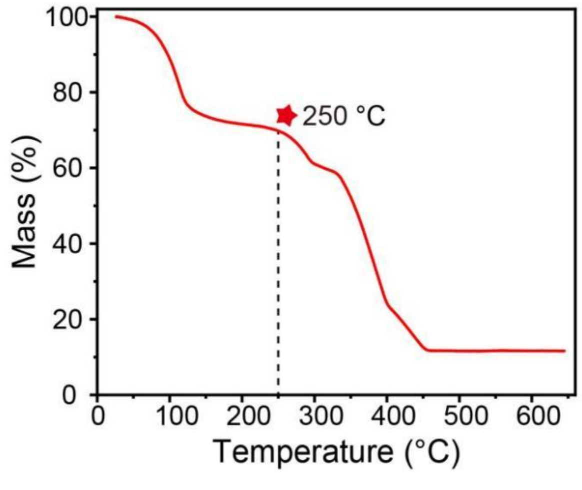

Fig. S13 TGA curve of ZNU-2-Si. The weight loss between 20-110 ºC is because of the loss of MeOH and water from the sample. The weight keeps consistent until ~250 ºC.

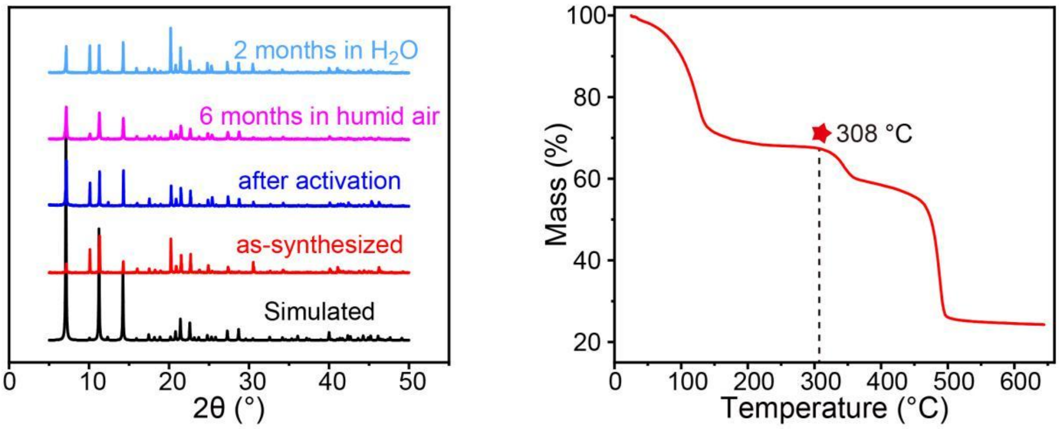

Fig. S14 PXRD patterns of ZNU-2-Ti after different treatments (Left). TGA curve of ZNU-2-Ti (Right). The weight loss between 30-110 ºC is because of the loss of MeOH and water from the sample. The weight keeps consistent until ~308 ºC.

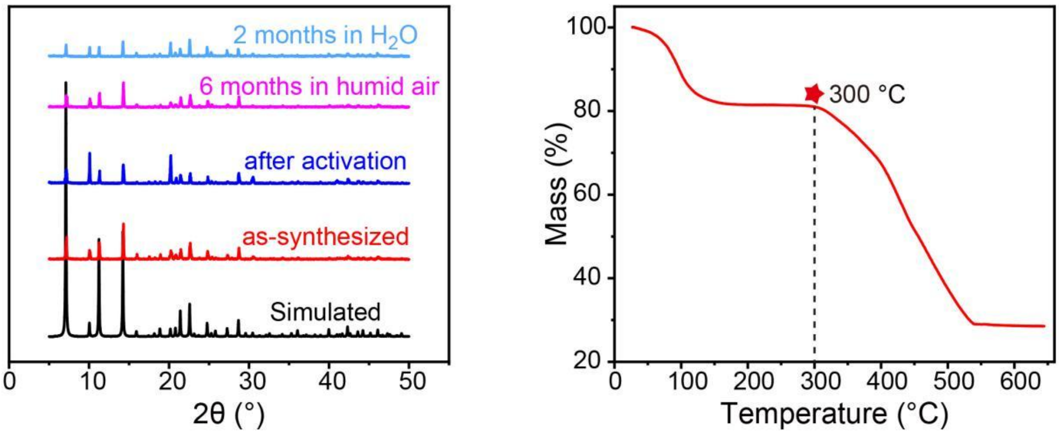

Fig. S15 PXRD patterns of ZNU-2-Nb after different treatments (Left). TGA curve of ZNU-2-Nb (Right). The weight loss between 30-110 ºC is because of the loss of MeOH and water from the sample. The weight keeps consistent until ~300 ºC.

III Adsorption data, IAST selectivity and *Qst*

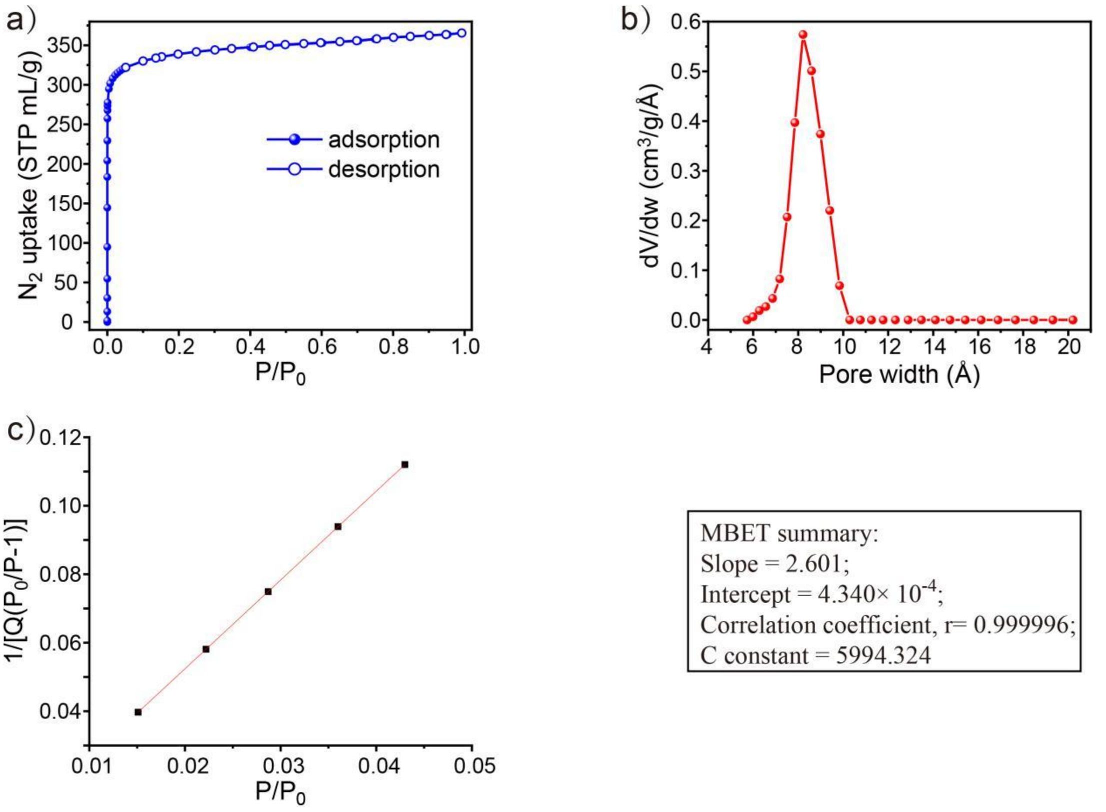

Fig. S16 (A) The adsorption and desorption isotherm of N_2 on ZNU-2-Si at 77 K. (B) The calculated pore size distribution of ZNU-2-Si. (C) Plot for the calculation of the BET surface area.

The BET surface area calculated from the N_2 adsorption isotherms under the pressure range of P/P_0 = 0.01-0.05 (for micropores) is 1339 m^2/g.

The total pore volume calculated from the N_2 adsorption isotherms is 0.565 cm^3/g.

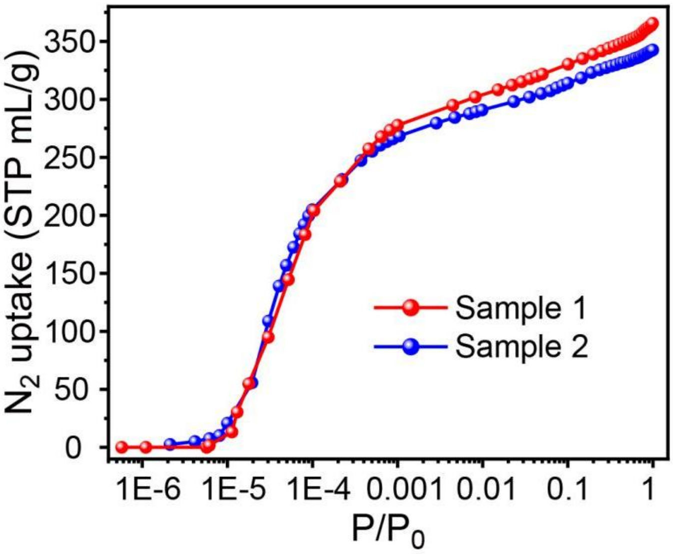

Fig. S17 The adsorption isotherms of N_2 on two batches of ZNU-2-Si samples at 77 K.

The adsorption curve of 77 K N_2 for sample 2 is approximately consistent with that for sample 2. This means that the synthesis of ZNU-2-Si is repeatable.

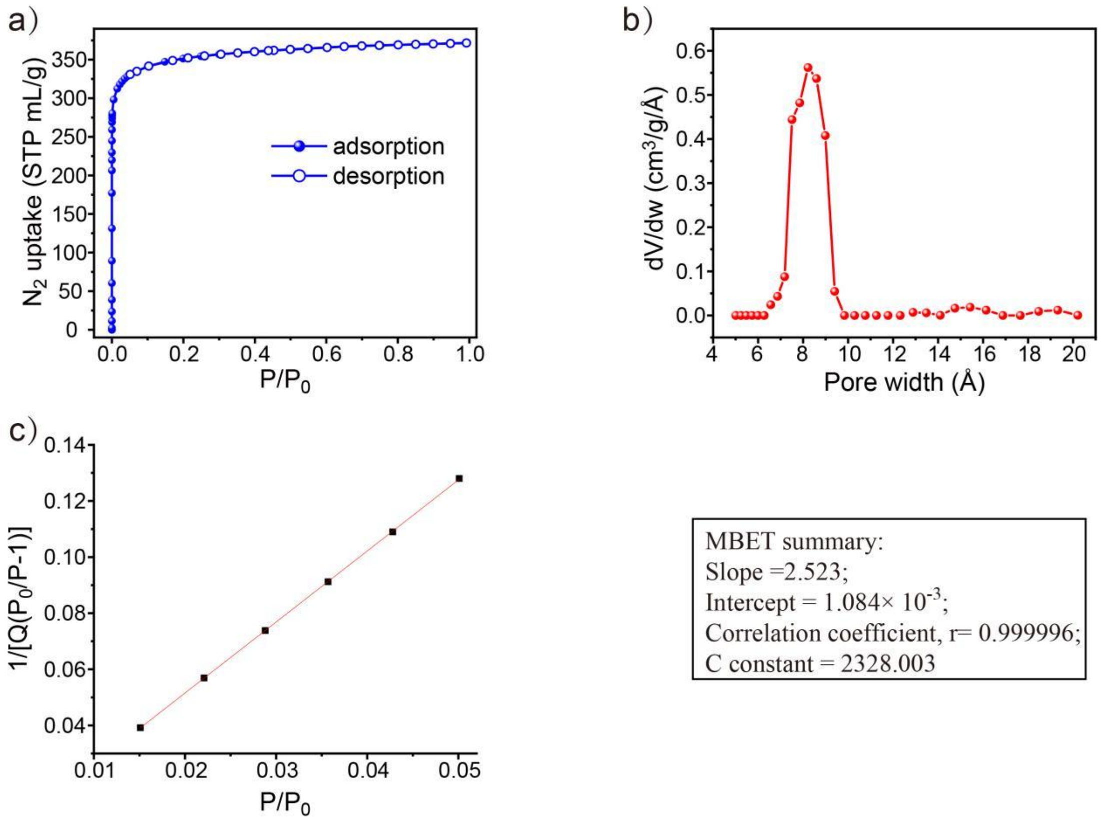

Fig. S18 (A) The adsorption and desorption isotherm of N_2 on ZNU-2-Ti at 77 K. (B) The calculated pore size distribution of ZNU-2-Ti. (C) Plot for the calculation of the BET surface area.

The BET surface area calculated from the N_2 adsorption isotherms under the pressure range of P/P_0 = 0.01-0.05 (for micropores) is 1380 m^2/g.

The total pore volume calculated from the N_2 adsorption isotherms is 0.575 cm^3/g.

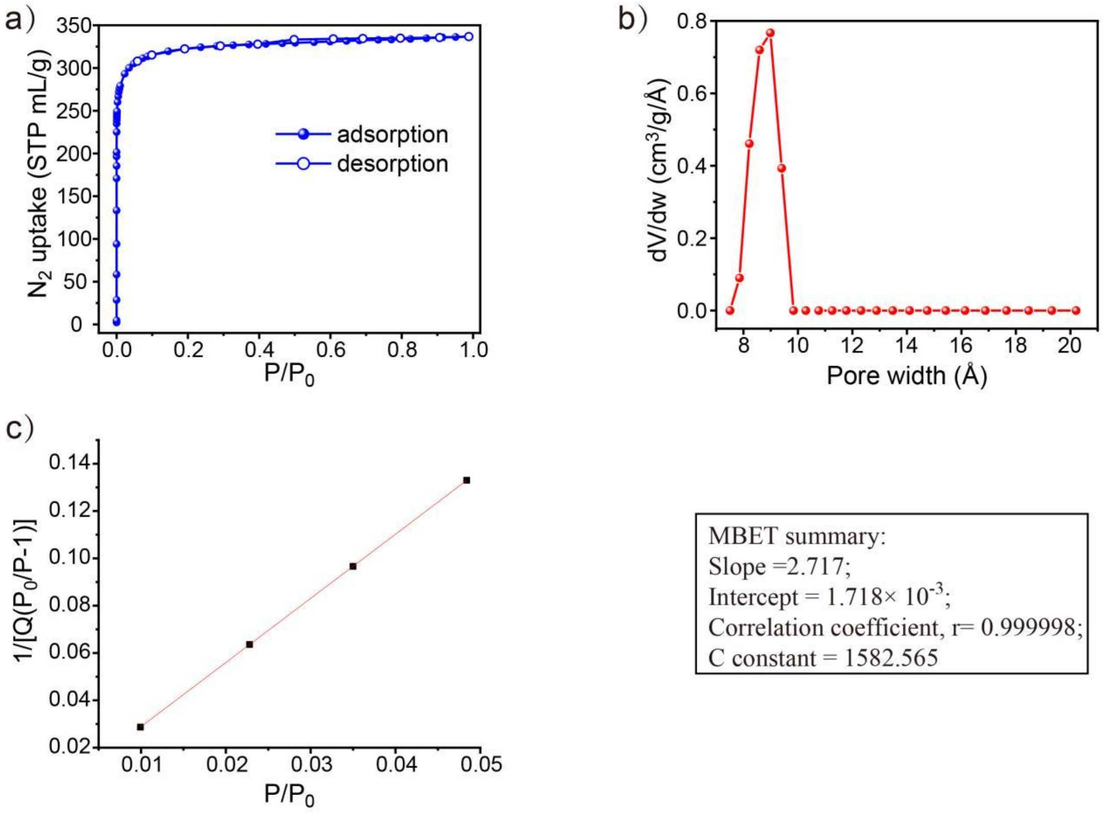

Fig. S19 (A) The adsorption and desorption isotherm of N_2 on ZNU-2-Nb at 77 K. (B) The calculated pore size distribution of ZNU-2-Nb. (C) Plot for the calculation of the BET surface area.

The BET surface area calculated from the N_2 adsorption isotherms under the pressure range of P/P_0 = 0.01-0.05 (for micropores) is 1281 m^2/g.

The total pore volume calculated from the N_2 adsorption isotherms is 0.521 cm^3/g.

Table S4 Comparison of C_3H_4, C_3H_6, C_2H_2, C_2H_4 and CO_2.

Gas molecules	Kinetic Diameter (Å)	Molecular size (Å^3)	Boiling point (K)	Polarizability ($\times10^{-25}$ cm^3)
C_3H_4	4.2	4.16 x 4.01 x 6.51	249.95	55.5
C_3H_6	4.6	4.65 x 4.16 x 6.44	225.45	62.6
C_2H_2	3.3	3.32 x 3.34 x 5.70	189.3	33.3-39.3
C_2H_4	4.2	3.28 x 4.18 x 4.84	169.5	42.5
CO_2	3.3	3.18 x 3.33 x 5.36	194.7	25.93

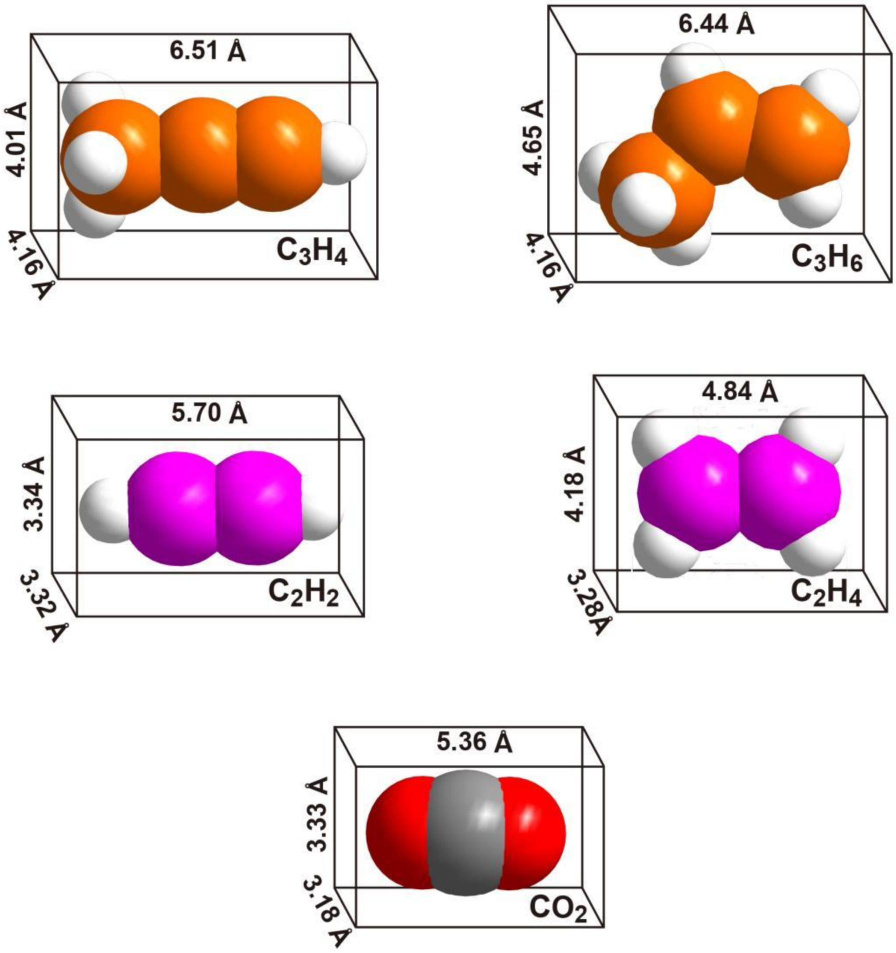

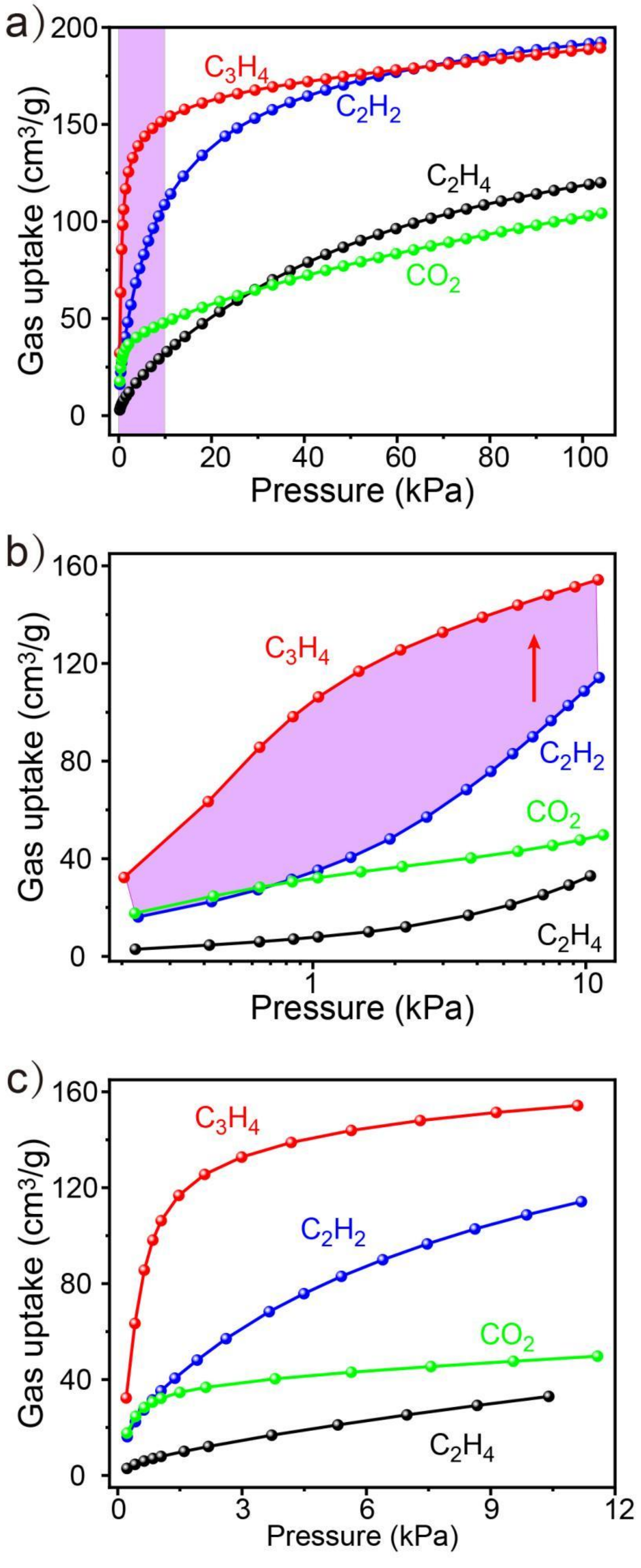

Fig. S20 Comparison of the C_3H_4, C_2H_2, CO_2, C_2H_4 adsorption isotherms on ZNU-2-Si (a: between 1-100 kPa, b, c: between 1-10 kPa).

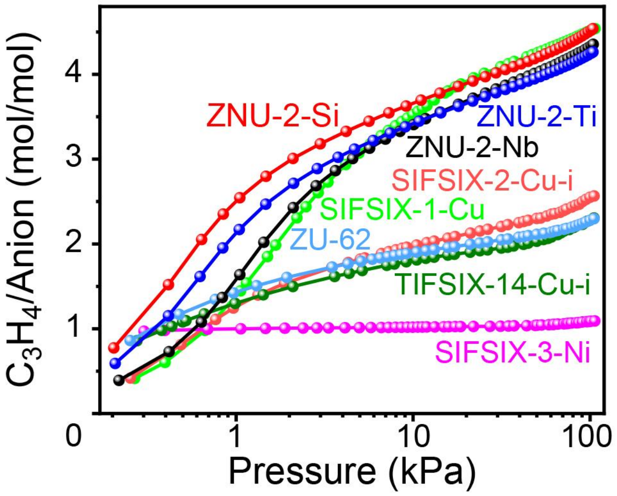

Fig. S21 Comparison of the C_3H_4 adsorption isotherms of the ZNU-2 family with fluorinated anion hybrid ultramicroporous materials.

Table S5 Comparison of the C_3H_4 adsorption isotherms of ZNU-2 family with fluorinated anion hybrid ultramicroporous materials.

	C_3H_4/Anion (mol/mol)			
	1 kPa	10 kPa	100 kPa	
**ZNU-2-Si**	**2.54**	**3.66**	**4.54**	**This work**
**ZNU-2-Ti**	**2.17**	**3.42**	**4.26**	**This work/[15]**
**ZNU-2-Nb**	**1.64**	**3.41**	**4.35**	**This work**
SIFSIX-1-Cu	1.45	3.53	4.52	This work/[5]
SIFSIX-2-Cu-i	1.25	1.97	2.57	This work/[5,13,14]
TIFSIX-14-Cu-i	1.30	1.80	2.31	This work/[13]
ZU-62	1.47	1.89	2.30	This work/[14]
SIFSIX-3-Ni	1.00	1.02	1.09	This work/[5]

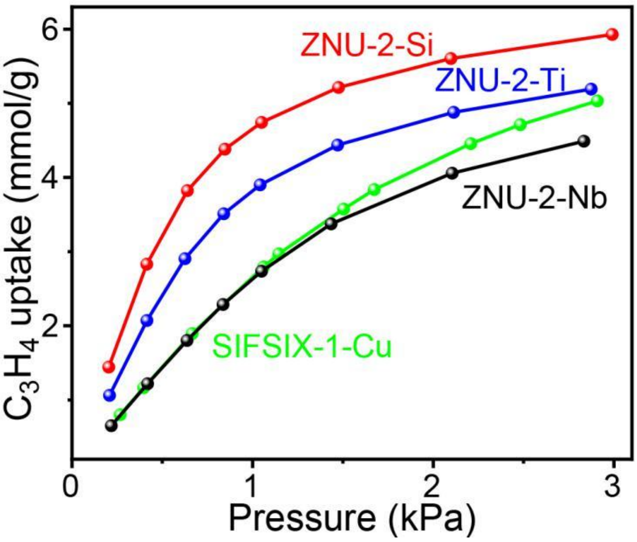

Fig. S22 Comparison of the C_3H_4 adsorption isotherms of the ZNU-2 family with SIFSIX-1-Cu between 1-3 kPa.

Table S6 Comparison of the C_3H_4 storage density of ZNU-2 family and liquid C_3H_4.

	storage density (g/cm^3)
ZNU-2-Si (298 K)	0.60
ZNU-2-Ti (298 K)	0.53
ZNU-2-Nb (298 K)	0.56
ZNU-2-Si (278 K)	0.65
C_3H_4 (l)	0.67

Storage density= Q (adsorption capacity, mmol/g) × M (relative molecular mass, mg/mmol) / V (pore volume, cm^3/g)

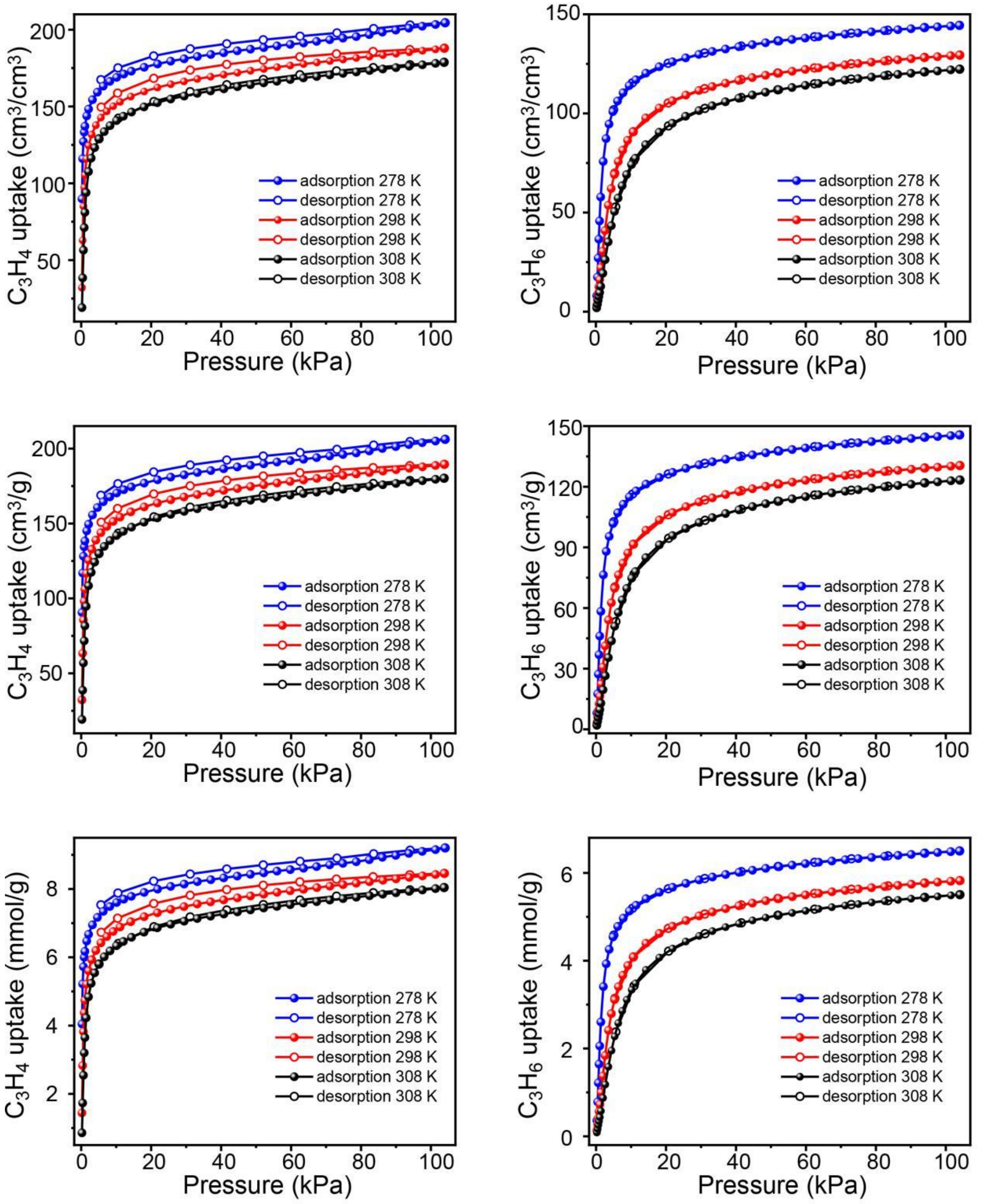

Fig. S23 The sorption isotherms of C_3H_4 and C_3H_6 on ZNU-2-Si at 278, 298, and 308 K in units of cm^3/cm^3, cm^3/g and mmol/g.

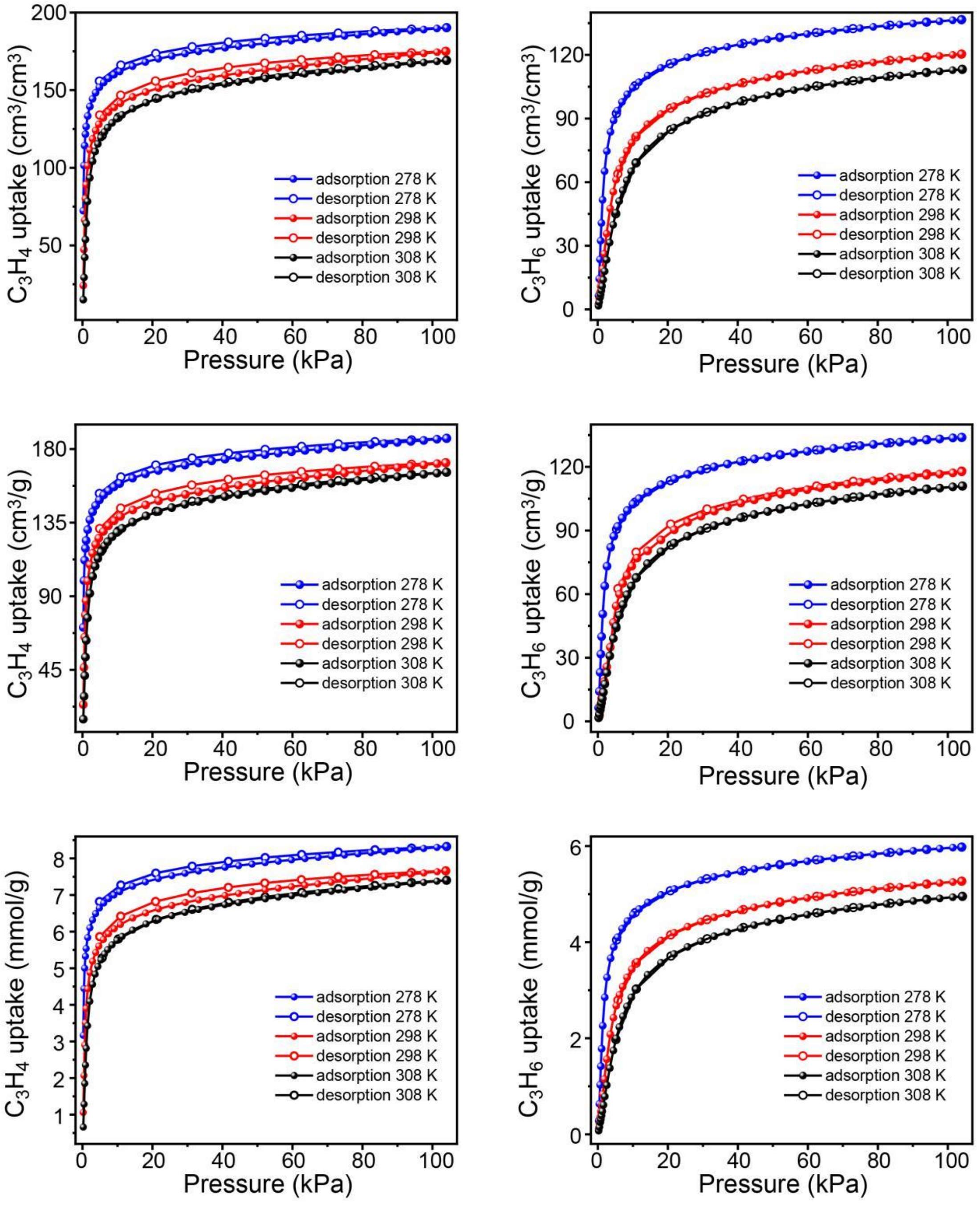

Fig. S24 The sorption isotherms of C_3H_4 and C_3H_6 on ZNU-2-Ti at 278, 298, and 308 K in units of cm^3/cm^3, cm^3/g and mmol/g.

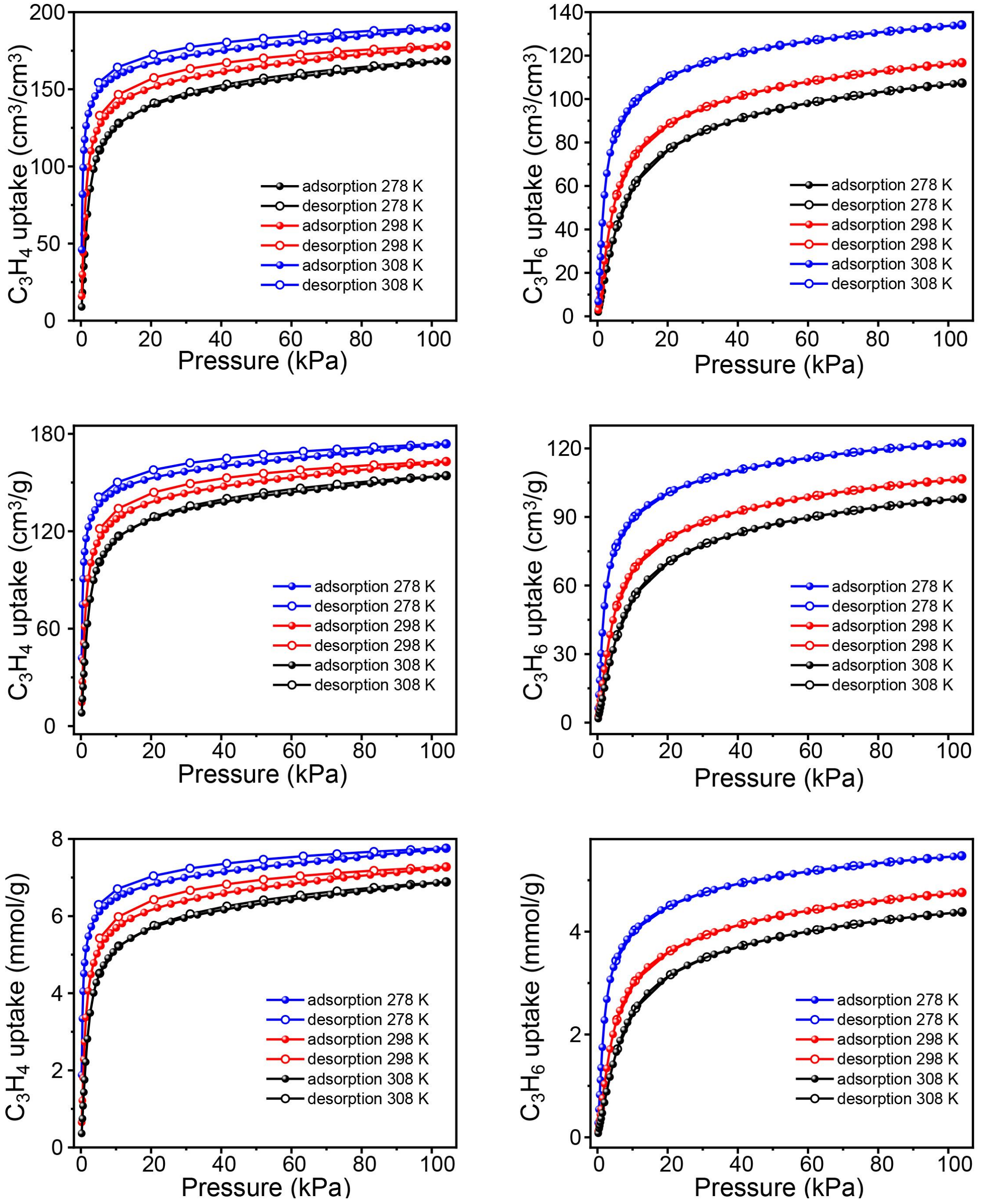

Fig. S25 The sorption isotherms of C_3H_4 and C_3H_6 on ZNU-2-Nb at 278, 298, and 308 K in units of cm^3/cm^3, cm^3/g and mmol/g.

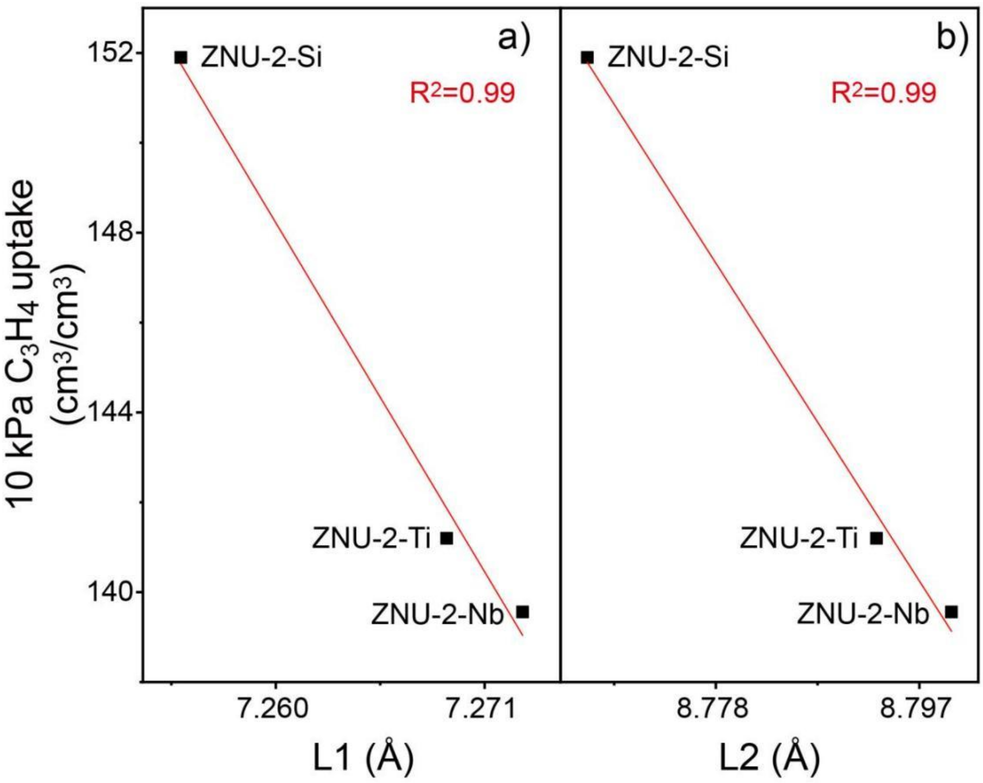

a)	y=-706.12 x + 5274.65	R^2=0.99
b)	y=-372.25 x + 3414.97	R^2=0.99

Fig. S26 Plots of the C_3H_4 uptake at 10 kPa on ZNU-2 and the dimensions of tridentate ligands (L1) and Cu-Cu distances (L2) of ZNU-2.

Table S7 Dual-site Langmuir-Freundlich fits for C_3H_4, and C_3H_6 in ZNU-2-Si.

	Site A				Site B			
	$q_{A,sat}$ mol kg^{-1}	b_{A0} Pa$^{-\nu A}$	E_A kJ mol^{-1}	νA	$q_{B,sat}$ mol kg^{-1}	b_{B0} Pa$^{-\nu B}$	E_B kJ mol^{-1}	νB
C_3H_4	6.42	6.616E-14	55.4	1.28	2.8	4.036E-13	44	1
C_3H_6	5.2	4.575E-13	44.3	1.26	1.65	2.132E-15	54.2	1

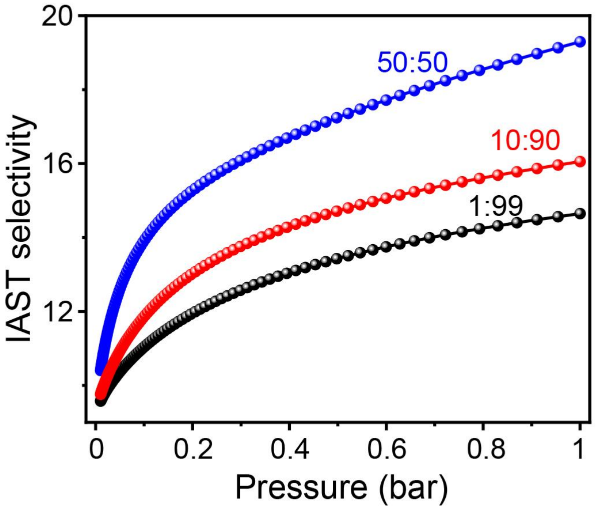

Fig. S27 IAST selectivity of ZNU-2-Si towards gas mixtures of C_3H_4/C_3H_6 (50/50 10/90 and 1/99) at 298 K.

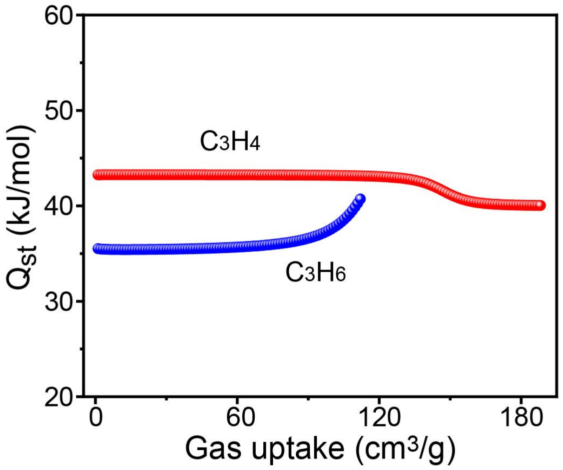

Fig. S28 The isosteric heat of adsorption, Q_{st}, for C_3H_4 and C_3H_6 on ZNU-2-Si.

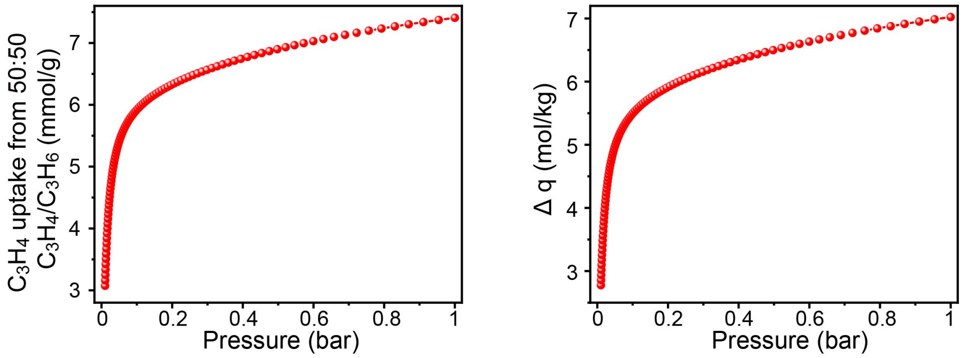

Fig. S29 Left: the adsorption isotherm of C_3H_4 from C_3H_4/C_3H_6 (50/50) mixture on ZNU-2-Si. Right: IAST based separation potential for C_3H_4/C_3H_6 (50/50) mixtures.

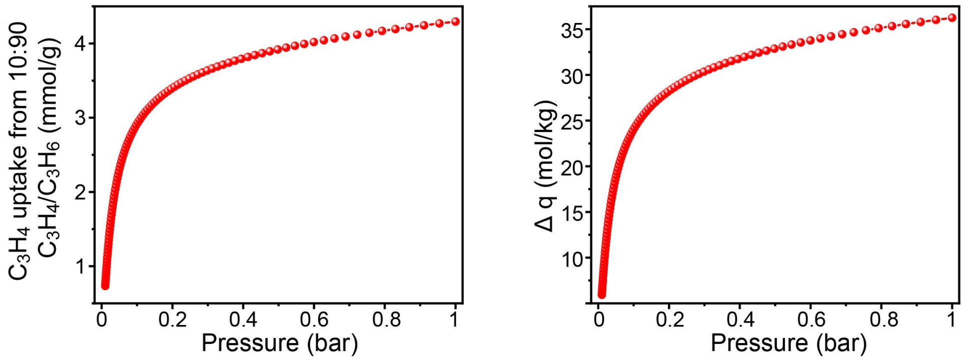

Fig. S30 Left: the adsorption isotherm of C_3H_4 from C_3H_4/C_3H_6 (10/90) mixture on ZNU-2-Si. Right: IAST based separation potential for C_3H_4/C_3H_6 (10/90) mixtures.

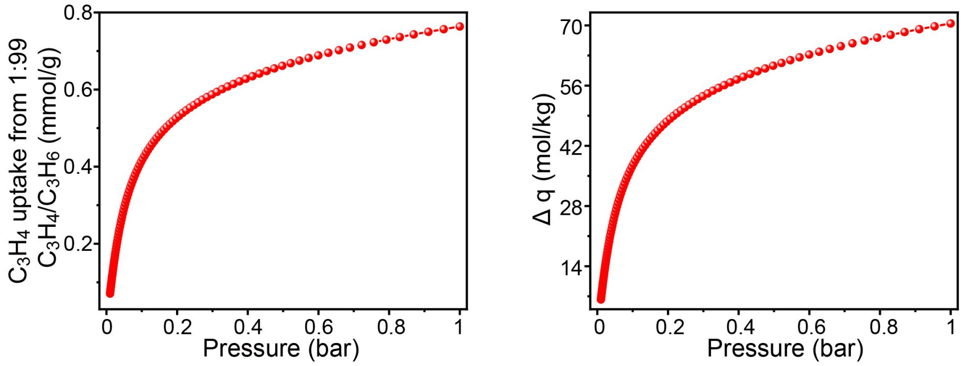

Fig. S31 Left: the adsorption isotherm of C_3H_4 from C_3H_4/C_3H_6 (1/99) mixture on ZNU-2-Si. Right: IAST based separation potential for C_3H_4/C_3H_6 (1/99) mixtures.

Table S8 Dual-site Langmuir-Freundlich fits for C_3H_4, and C_3H_6 in ZNU-2-Ti.

	Site A				Site B			
	$q_{A,sat}$ mol/kg	b_{A0} Pa^{-1}	E_A kJ/mol	νA	$q_{B,sat}$ mol/kg	b_{B0} Pa^{-1}	E_B kJ/mol	νB
C_3H_4	6	1.387E-13	53.3	1.24	2.5	3.443E-13	44.2	1
C_3H_6	4.4	3.417E-13	44.4	1.3	2.1	6.722E-14	46	1

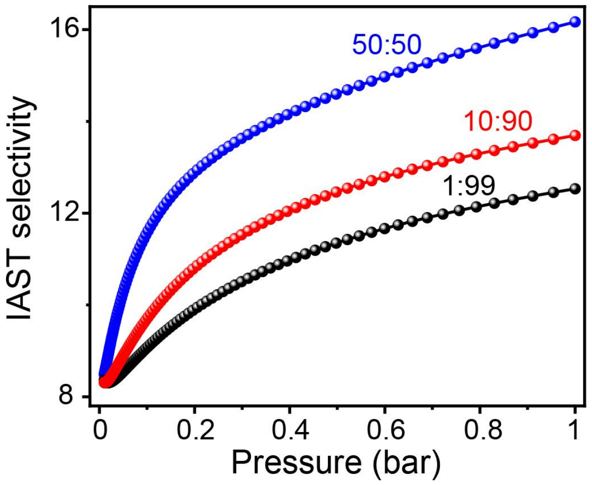

Fig. S32 IAST selectivity of ZNU-2-Ti towards gas mixtures of C_3H_4/C_3H_6 (50/50 10/90 and 1/99) at 298 K.

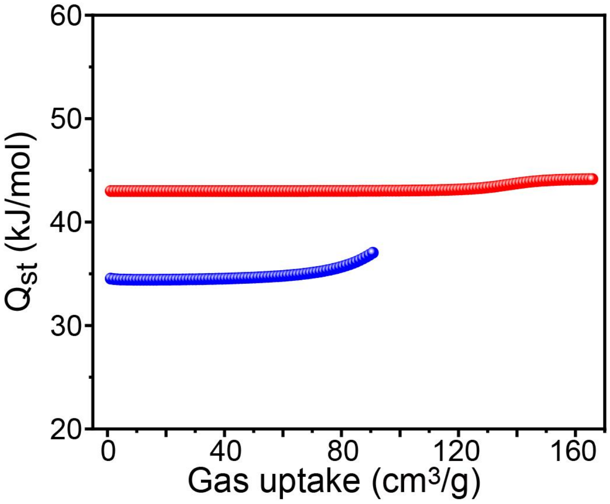

Fig. S33 The isosteric heat of adsorption, Q_{st}, for C_3H_4 and C_3H_6 on ZNU-2-Ti.

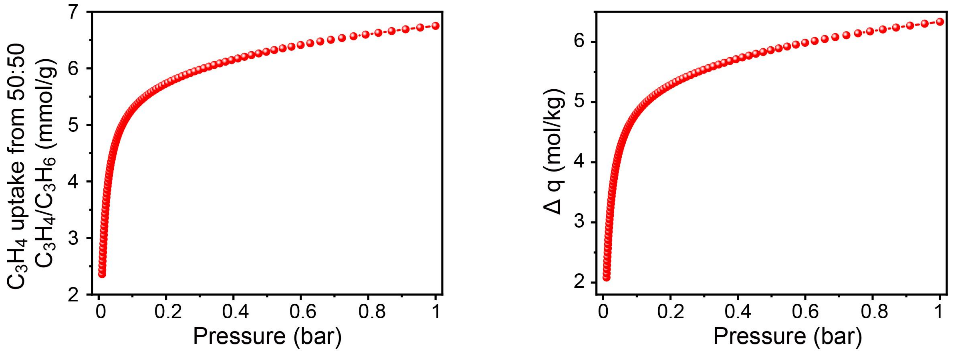

Fig. S34 Left: the adsorption isotherm of C_3H_4 from C_3H_4/C_3H_6 (50/50) mixture on ZNU-2-Ti. Right: IAST based separation potential for C_3H_4/C_3H_6 (50/50) mixtures.

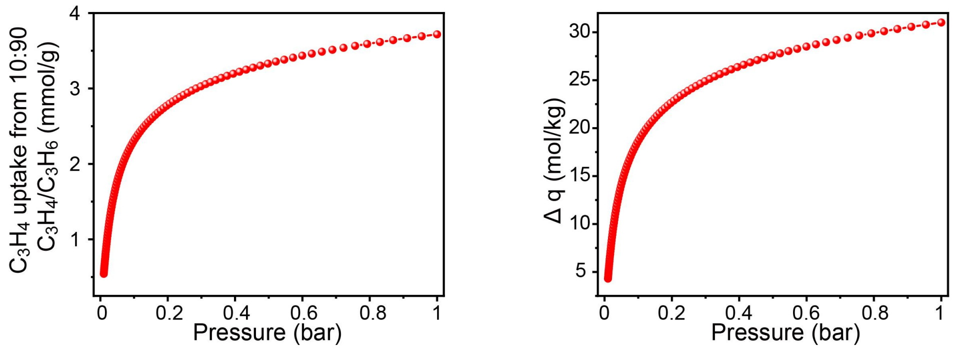

Fig. S35 Left: the adsorption isotherm of C_3H_4 from C_3H_4/C_3H_6 (10/90) mixture on ZNU-2-Ti. Right: IAST based separation potential for C_3H_4/C_3H_6 (10/90) mixtures.

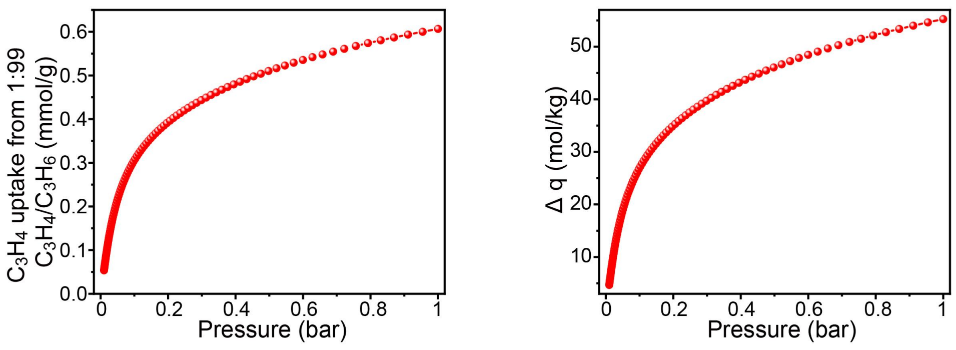

Fig. S36 Left: the adsorption isotherm of C_3H_4 from C_3H_4/C_3H_6 (1/99) mixture on ZNU-2-Ti. Right: IAST based separation potential for C_3H_4/C_3H_6 (1/99) mixtures.

Table S9 Dual-site Langmuir-Freundlich fits for C_3H_4, and C_3H_6 in ZNU-2-Nb.

	Site A				Site B			
	$q_{A,sat}$ mol kg^{-1}	b_{A0} Pa$^{-\nu A}$	E_A kJ mol^{-1}	νA	$q_{B,sat}$ mol kg^{-1}	b_{B0} Pa$^{-\nu B}$	E_B kJ mol^{-1}	νB
C_3H_4	5.9	3.368E-13	50	1.2	2.2	1.716E-12	39.2	1
C_3H_6	4.2	2.960E-11	36	1.13	1.6	1.972E-16	60	1

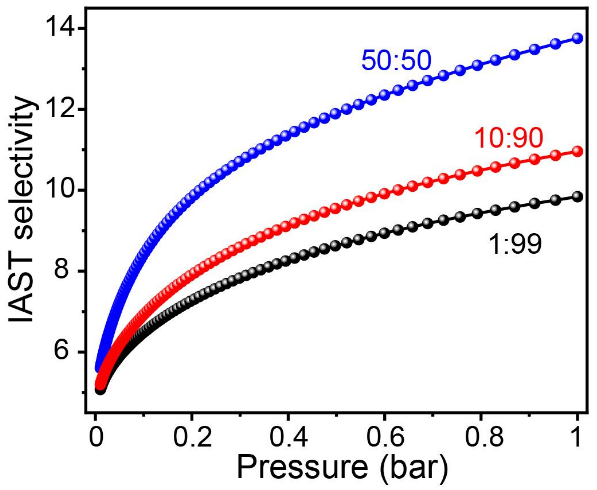

Fig. S37 IAST selectivity of ZNU-2-Nb towards gas mixtures of C_3H_4/C_3H_6 (50/50 10/90 and 1/99) at 298 K.

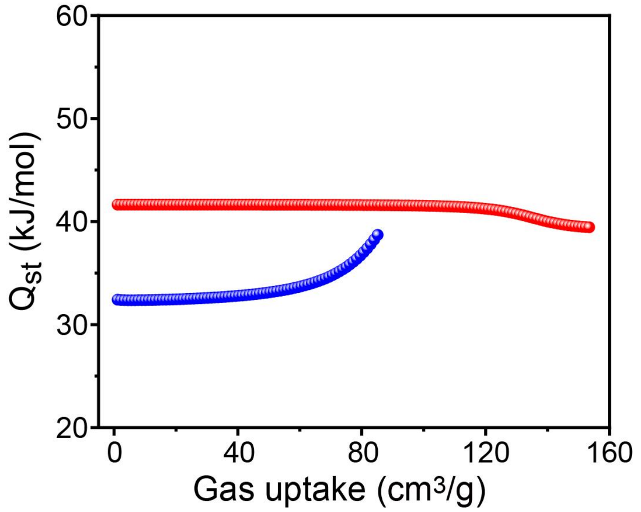

Fig. S38 The isosteric heat of adsorption, Q_{st}, for C_3H_4 and C_3H_6 on ZNU-2-Nb.

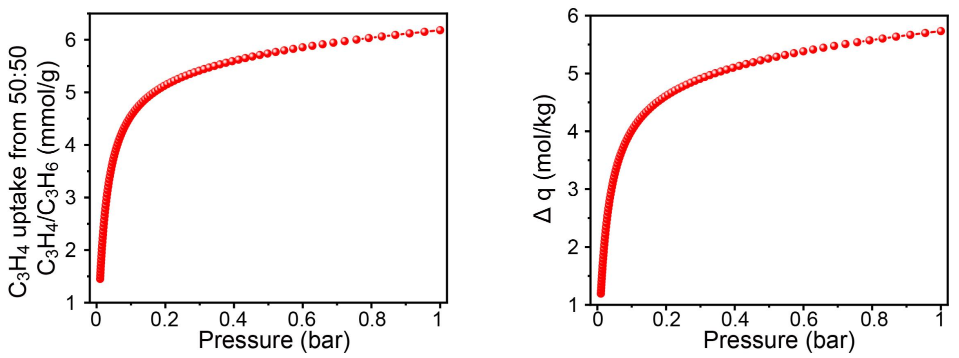

Fig. S39 Left: the adsorption isotherm of C_3H_4 from C_3H_4/C_3H_6 (50/50) mixture on ZNU-2-Nb. Right: IAST based separation potential for C_3H_4/C_3H_6 (50/50) mixtures.

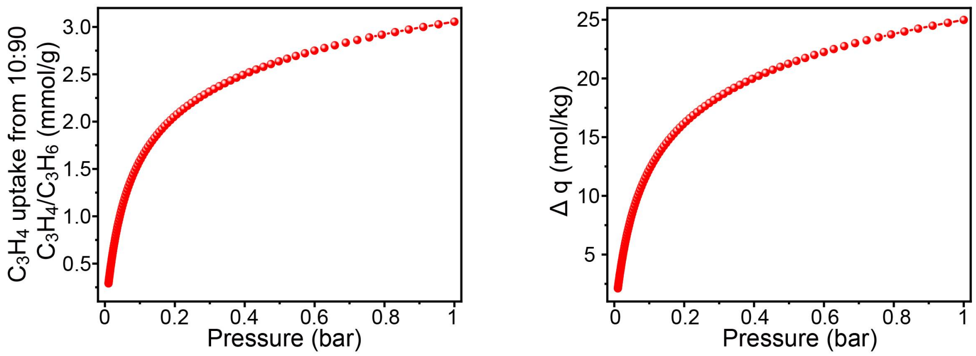

Fig. S40 Left: the adsorption isotherm of C_3H_4 from C_3H_4/C_3H_6 (10/90) mixture on ZNU-2-Nb. Right: IAST based separation potential for C_3H_4/C_3H_6 (10/90) mixtures.

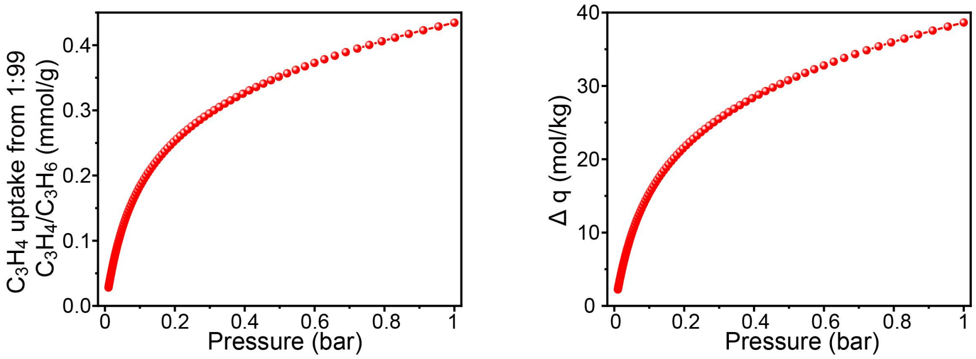

Fig. S41 Left: the adsorption isotherm of C_3H_4 from C_3H_4/C_3H_6 (1/99) mixture on ZNU-2-Nb. Right: IAST based separation potential for C_3H_4/C_3H_6 (1/99) mixtures.

Fig. S10 Unary isotherm fit parameters for C_3H_4 and C_3H_6 in SIFSIX-1-Cu at 298 K.

	Site A			Site B		
	$q_{A,sat}$ mol kg^{-1}	b_A $Pa^{-\nu A}$	ν_A dimensionless	$q_{B,sat}$ mol kg^{-1}	b_B $Pa^{-\nu B}$	ν_B dimensionless
C_3H_4	8	5.815E-07	1	8.4	4.451E-04	1
C_3H_6	2.4	7.168E-10	2.45	4	6.642E-05	1

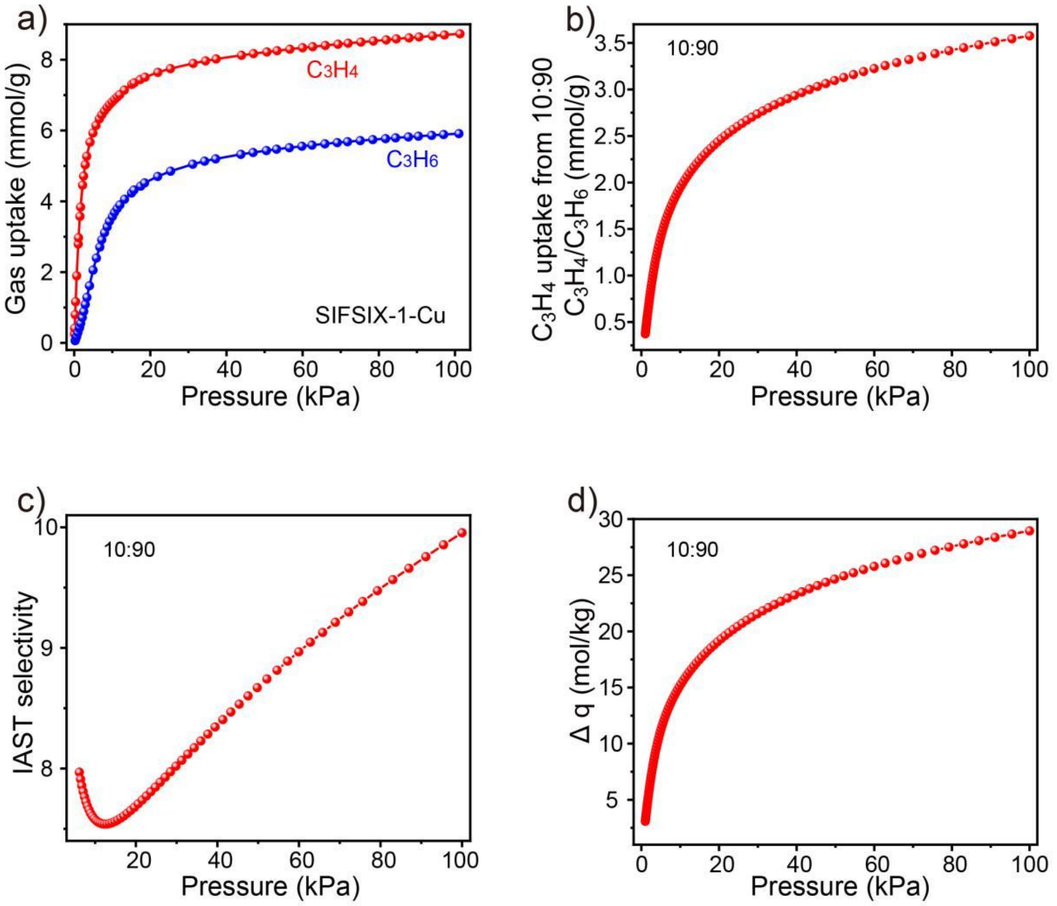

Fig. S42 (a) C_3H_4 and C_3H_6 adsorption isotherms for SIFSIX-1-Cu at 298 K. (b) IAST selectivity of SIFSIX-1-Cu towards gas mixtures of C_3H_4/C_3H_6 (10/90). (c) The adsorption isotherm of C_3H_4 from C_3H_4/C_3H_6 (10/90) mixture on SIFSIX-1-Cu. (d) IAST based separation potential for C_3H_4/C_3H_6 (10/90) mixtures.

Table S11 Unary isotherm fit parameters for C_3H_4 and C_3H_6 in SIFSIX-2-Cu-i at 298 K.

	Site A			Site B		
	$q_{A,sat}$ mol kg^{-1}	b_A $Pa^{-\nu A}$	ν_A dimensionless	$q_{B,sat}$ mol kg^{-1}	b_B $Pa^{-\nu B}$	ν_B dimensionless
C_3H_4	8.2	1.544E-06	1	3.5	1.390E-03	1
C_3H_6	1.1	1.248E-04	1	2.3	3.052E-05	1

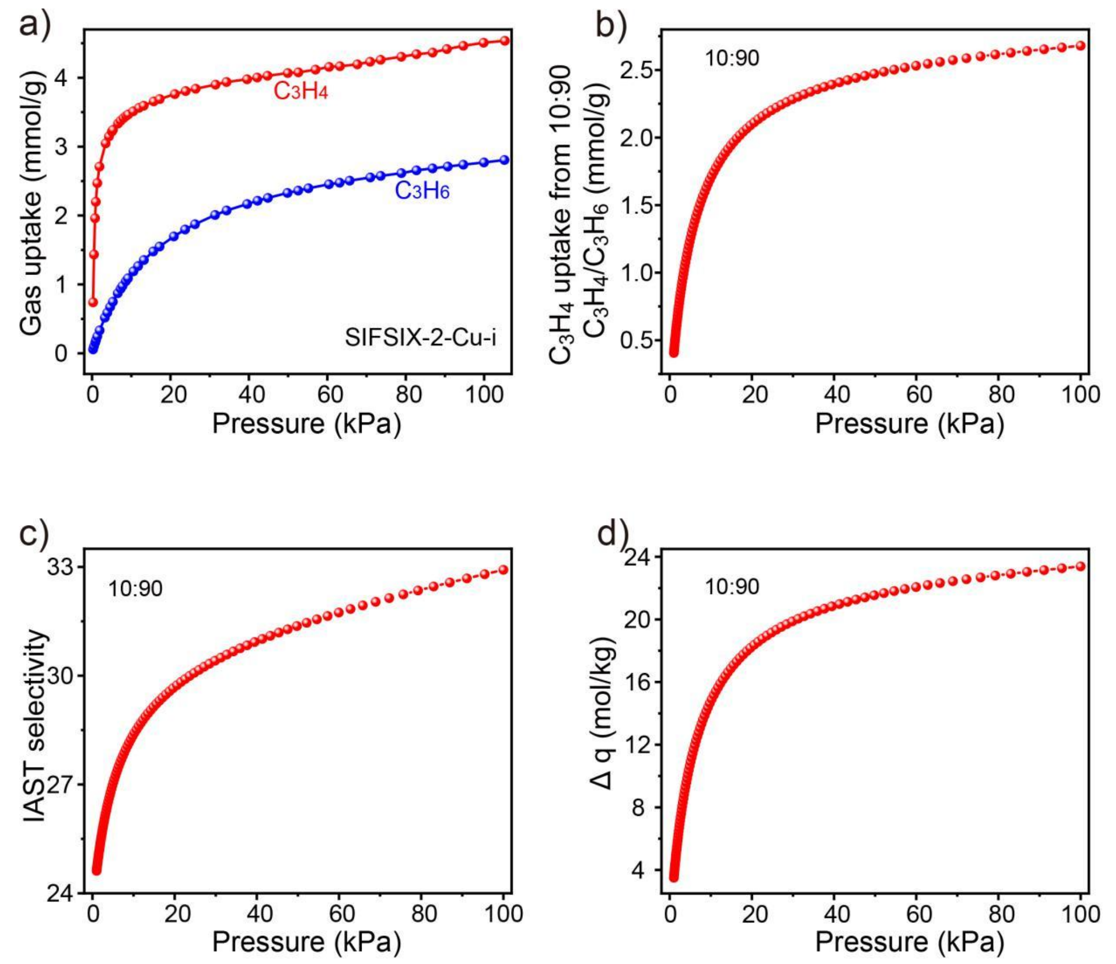

Fig. S43 (a) C_3H_4 and C_3H_6 adsorption isotherms for SIFSIX-2-Cu-i at 298 K. b) IAST selectivity of SIFSIX-2-Cu-i towards gas mixtures of C_3H_4/C_3H_6 (10/90). (c) The adsorption isotherm of C_3H_4 from C_3H_4/C_3H_6 (10/90) mixture on SIFSIX-2-Cu-i. (d) IAST based separation potential for C_3H_4/C_3H_6 (10/90) mixtures.

Table S12 Unary isotherm fit parameters for C_3H_4 and C_3H_6 in SIFSIX-3-Ni at 298 K.

	Site A			Site B		
	$q_{A,sat}$ mol kg^{-1}	b_A $Pa^{-\nu A}$	ν_A dimensionless	$q_{B,sat}$ mol kg^{-1}	b_B $Pa^{-\nu B}$	ν_B dimensionless
C_3H_4	0.6	6.453E-03	0.42	2.65	7.240E-04	2
C_3H_6	2.8	1.152E-05	1.23			

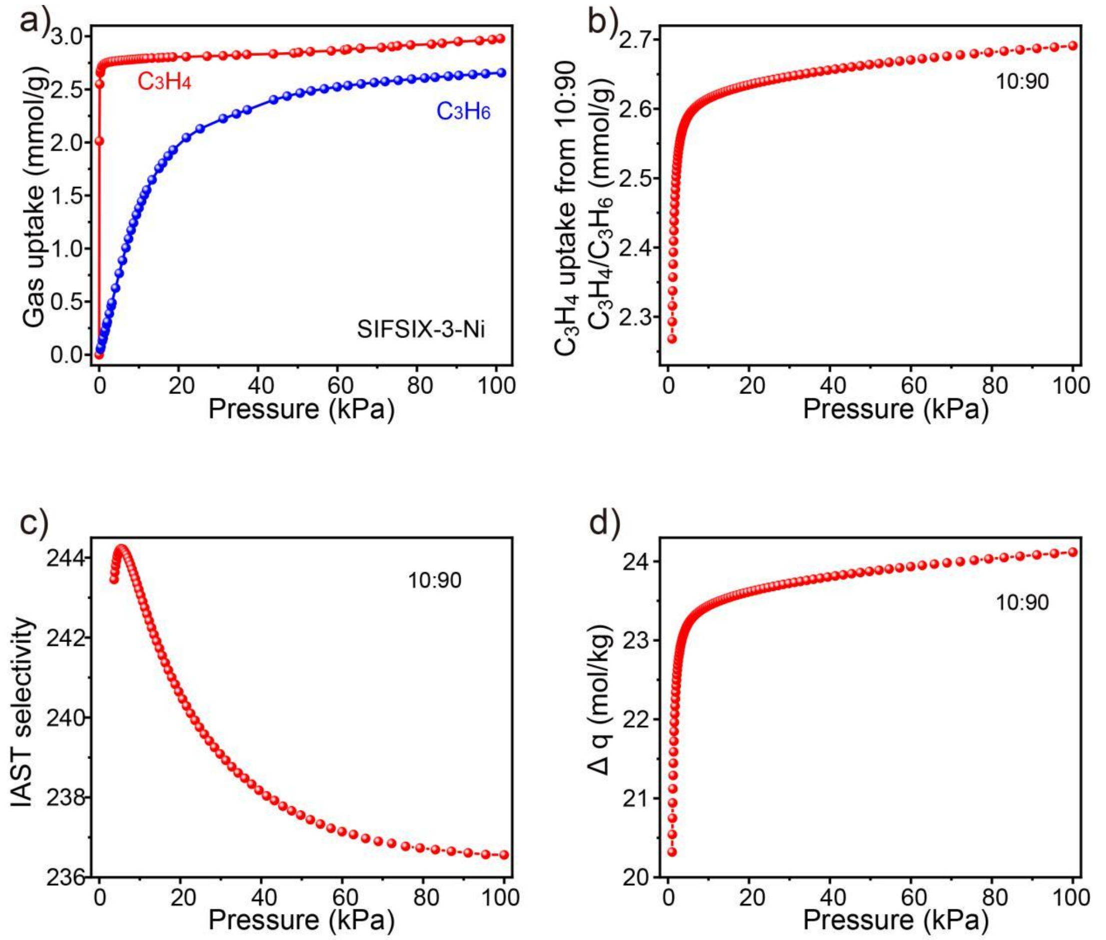

Fig. S44 (a) C_3H_4 and C_3H_6 adsorption isotherms for SIFSIX-3-Ni at 298 K. (b) IAST selectivity of SIFSIX-3-Ni towards gas mixtures of C_3H_4/C_3H_6 (10/90). (c) The adsorption isotherm of C_3H_4 from C_3H_4/C_3H_6 (10/90) mixture on SIFSIX-3-Ni. (d) IAST based separation potential for C_3H_4/C_3H_6 (10/90) mixtures.

Table S13 Unary isotherm fit parameters for C_3H_4, and C_3H_6 in ZU-62 at 298 K.

	Site A			Site B		
	$q_{A,sat}$ mol kg^{-1}	b_A $Pa^{-\nu A}$	ν_A dimensionless	$q_{B,sat}$ mol kg^{-1}	b_B $Pa^{-\nu B}$	ν_B dimensionless
C_3H_4	8	4.965E-04	0.47	2.8	3.564E-03	1
C_3H_6	0.8	3.313E-17	4.6	2.3	4.092E-05	1

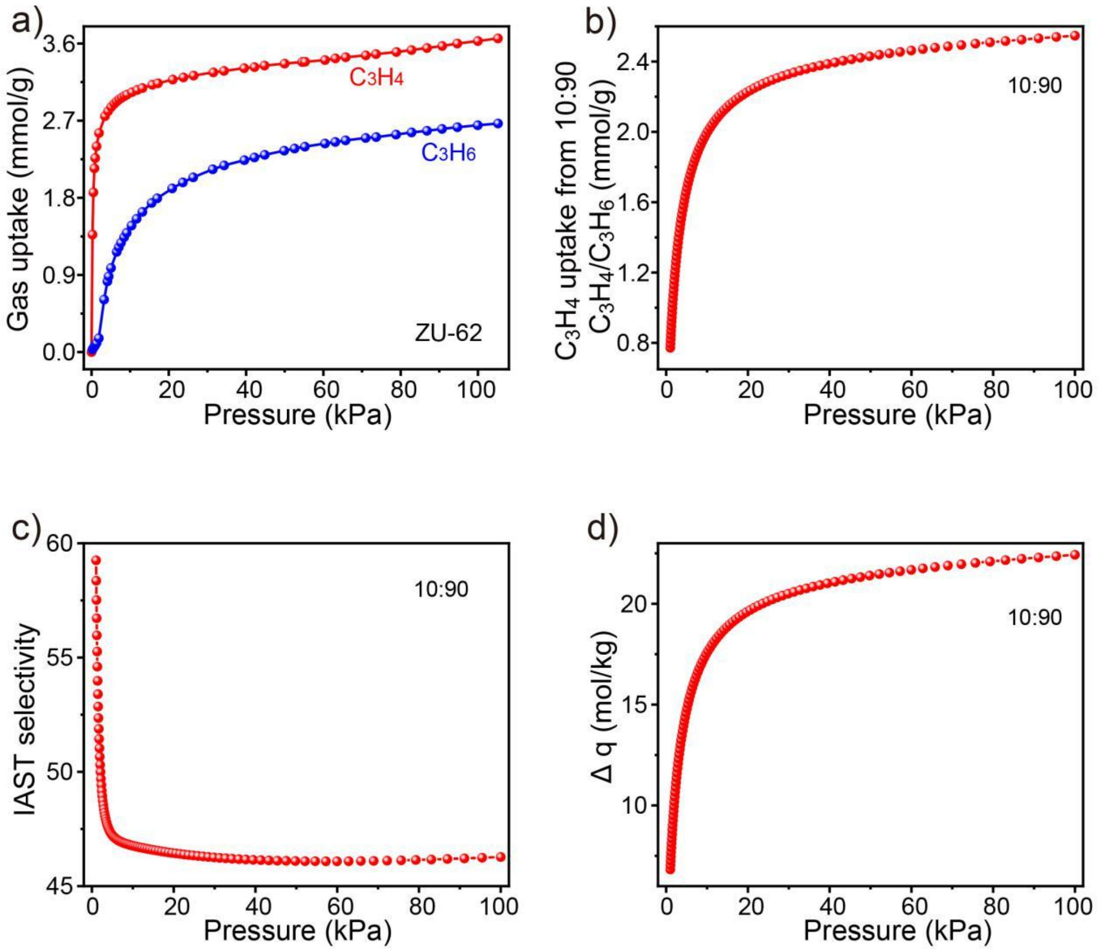

Fig. S45 (a) C_3H_4 and C_3H_6 adsorption isotherms for ZU-62 at 298 K. (b) IAST selectivity of ZU-62 towards gas mixtures of C_3H_4/C_3H_6 (10/90). (c) The adsorption isotherm of C_3H_4 from C_3H_4/C_3H_6 (10/90) mixture on ZU-62. (d) IAST based separation potential for C_3H_4/C_3H_6 (10/90) mixtures.

Table S14 Unary isotherm fit parameters for C_3H_4, and C_3H_6 in SIFSIX-14-Cu-i at 298 K.

	Site A			Site B		
	$q_{A,sat}$ mol kg^{-1}	b_A $Pa^{-\nu A}$	ν_A dimensionless	$q_{B,sat}$ mol kg^{-1}	b_B $Pa^{-\nu B}$	ν_B dimensionless
C_3H_4	1.7	1.879E-03	0.64	2.2	3.746E-18	6.35
C_3H_6	1.15	2.672E-81	18	20	9.099E-06	0.67

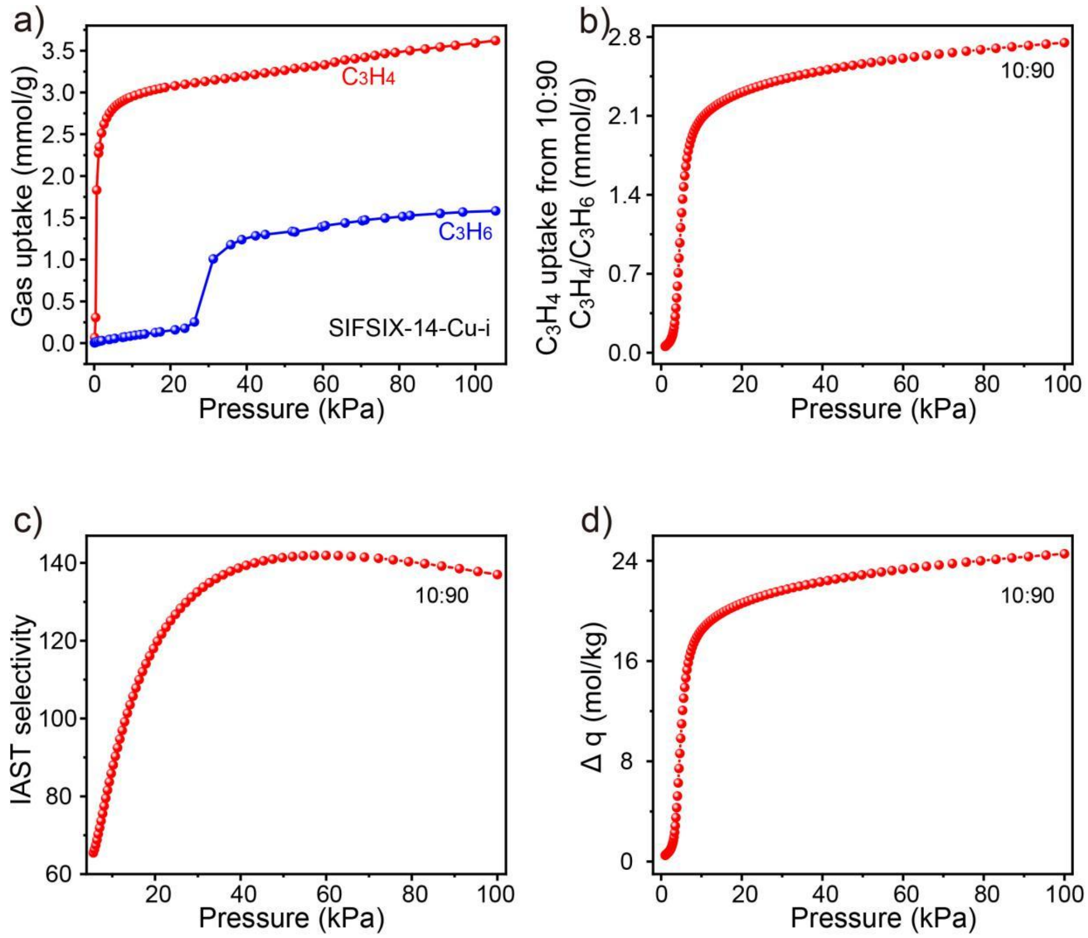

Fig. S46 (a) C_3H_4 and C_3H_6 adsorption isotherms for SIFSIX-14-Cu-i at 298 K. (b) IAST selectivity of SIFSIX-14-Cu-i towards gas mixtures of C_3H_4/C_3H_6 (10/90). (c) The adsorption isotherm of C_3H_4 from C_3H_4/C_3H_6 (10/90) mixture on SIFSIX-14-Cu-i. (d) IAST based separation potential for C_3H_4/C_3H_6 (10/90) mixtures.

Table S15 Unary isotherm fit parameters for C_3H_4, and C_3H_6 in GeFSIX-14-Cu-i at 298 K.

	Site A			Site B		
	$q_{A,sat}$ mol kg^{-1}	b_A Pa$^{-\nu A}$	ν_A dimensionless	$q_{B,sat}$ mol kg^{-1}	b_B Pa$^{-\nu B}$	ν_B dimensionless
C_3H_4	1.4	3.778E-04	1	1.9	1.316E-10	4.08
C_3H_6	1.12	1.183E-45	10	4	5.019E-05	0.65

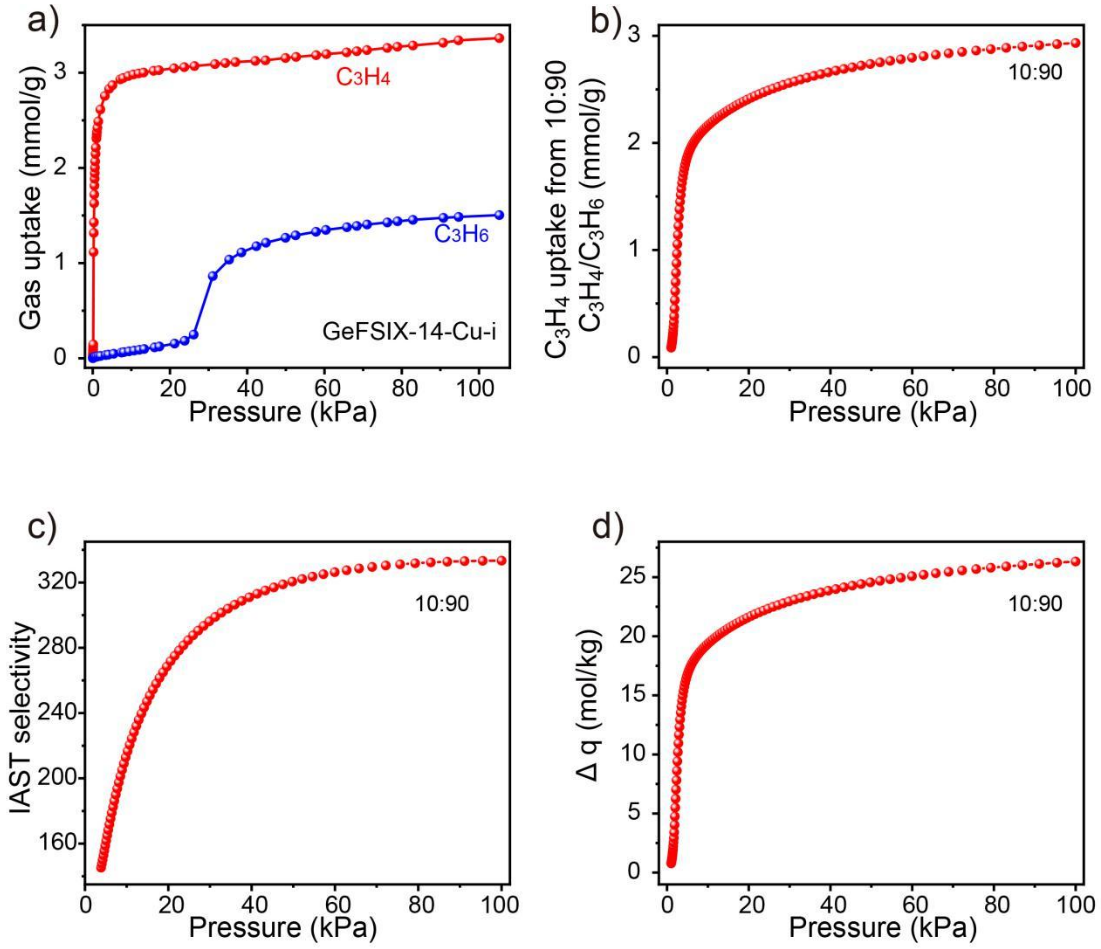

Fig. S47 (a) C_3H_4 and C_3H_6 adsorption isotherms for GeFSIX-14-Cu-i at 298 K. (b) IAST selectivity of GeFSIX-14-Cu-i towards gas mixtures of C_3H_4/C_3H_6 (10/90). (c) The adsorption isotherm of C_3H_4 from C_3H_4/C_3H_6 (10/90) mixture on GeFSIX-14-Cu-i. (d) IAST based separation potential for C_3H_4/C_3H_6 (10/90) mixtures.

Table S16 Unary isotherm fit parameters for C_3H_4, and C_3H_6 in TIFSIX-14-Cu-i at 298 K.

	Site A			Site B		
	$q_{A,sat}$ mol kg^{-1}	b_A $Pa^{-\nu A}$	ν_A dimensionless	$q_{B,sat}$ mol kg^{-1}	b_B $Pa^{-\nu B}$	ν_B dimensionless
C_3H_4	2	3.869E-04	1	1.5	8.723E-07	3
C_3H_6	1.77	4.129E-06	1.2			

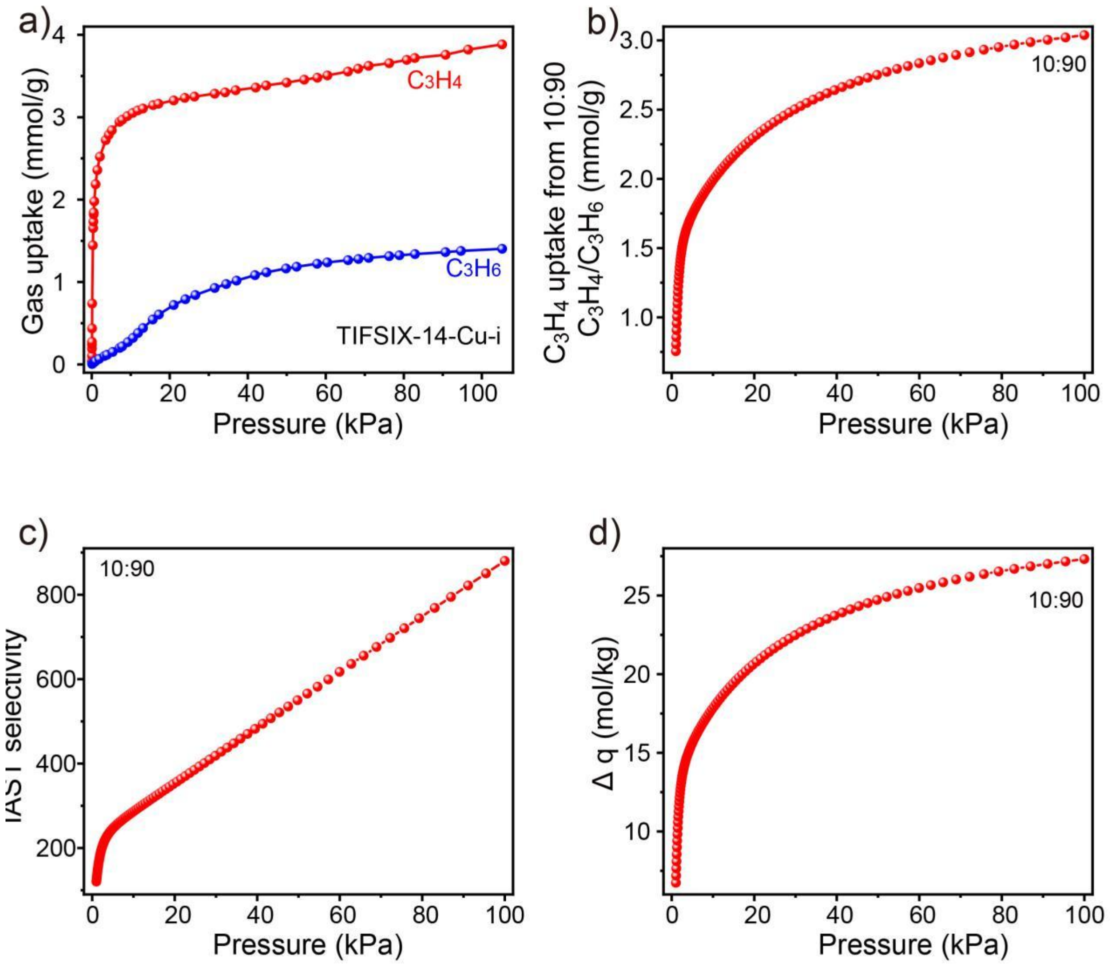

Fig. S48 (a) C_3H_4 and C_3H_6 adsorption isotherms for TIFSIX-14-Cu-i at 298 K. (b) IAST selectivity of TIFSIX-14-Cu-i towards gas mixtures of C_3H_4/C_3H_6 (10/90). (c) The adsorption isotherm of C_3H_4 from C_3H_4/C_3H_6 (10/90) mixture on TIFSIX-14-Cu-i. (d) IAST based separation potential for C_3H_4/C_3H_6 (10/90) mixtures.

Table S17 Comparison of the reported materials on C_3H_4 adsorption capacity at 1 kPa, 10 kPa and 100 kPa, and IAST selectivity towards C_3H_4/C_3H_6.

	C_3H_4 uptake (mmol/g)			Selectivity	Ref
	1 kPa	10kPa	100kPa	1:99	
ELM-12	1.83	2.54	2.74	84	[1]
ZJUT-1	0.35	1.07	2.28	70	[2]
NKMOF-11	1.78	2.12	3.10	1074	[3]
JXNU-6	0.36	2.59	5.07	3.1	[4]
NbOFFIVE-1-Ni	1.70	1.72	1.89	882	[5]
UTSA-200	2.99	3.30	3.62	20000	[6]
NKMOF-1-Ni	1.85	2.38	3.50	630.4[a]	[7]
NKMOF-1-Cu	2.03	2.35	3.33	610.5[a]	[7]
GeFSIX-dps-Cu	0.41	3.1	3.73	39.24[b]	[8]
HOF-30	1.15	1.79	2.67	7.7	[9]
Co-gallate	1.21	2.23	3.20	152	[10]
Mg-gallate	1.15	2.70	3.74	65	[10]
Ni-gallate	0.82	1.82	2.64	113	[10]

Continued

Ca-based MOF	2.60	2.79	3.01	38^{c}	[11]
MIL-100 (Cr)	1.52	4.98	14.52	4.5	[6]
ZIF-8	0.13	1.44	6.28	1.9	[6]
Cu-BTC	1.47	8.17	10.48	3.2	[6]
SIFSIX-3-Zn	2.05	2.11	2.26	115	[5]
ZU-16-Co	2.45	2.47	2.58	248	[12]
TIFSIX-3-Ni	1.86	1.91	2.11	>10^{6}	[12]
FJI-W1	2.75	5.80	7.09	2.2	[35]
SIFSIX-1-Cu	2.79	6.82	8.72	8.97	[5]/This work
SIFSIX-2-Cu-i	2.21	3.48	4.51	30.58	[5,13,14]/This work
SIFSIX-3-Ni	2.73	2.79	2.97	242.06	[5]/This work
ZU-62	2.28	3.02	3.63	46.31	[14]/This work
SIFSIX-14-Cu-i	2.27	2.95	3.59	112.86	This work
TIFSIX-14-Cu-i	2.19	3.04	3.86	306.12	[13]/This work
GeFSIX-14-Cu-i	2.34	2.97	3.36	240.14	[13]/This work
**ZNU-2-Si**	**4.74**	**6.83**	**8.46**	**14.6/16.1^{b}/19.3^{d}**	**This work**
**ZNU-2-Ti**	**3.9**	**6.18**	**7.66**	**12.5/13.7^{b}/16.2^{d}**	**[15]/This work**
**ZNU-2-Nb**	**2.74**	**5.70**	**7.28**	**9.8/11.0^{b}/13.8^{d}**	**This work**
propyne/propylene: 0.5/99 ^{a}; 10/90 ^{b}; 0.5/99.5 ^{c}; 50/50^{d}					

UTSA-200 and SIFSIX-14-Cu-i feature the same crystal structures. However, as Li et al[6] claimed, the preparation of UTSA-200 needs careful control of the reaction condition and a small amount of impurities are easily produced during the production of UTSA-200, which would greatly affect the separation performance. Thus, for clarity, UTSA-200 refers to the material with slightly better performance reported by Li[8] and SIFSIX-14-Cu-i refers to our synthesized material in this work which is also true in the main text.

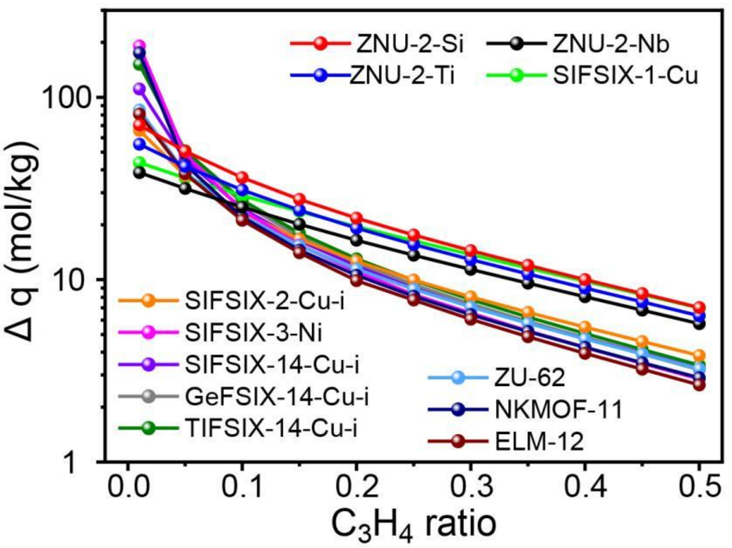

Fig. S49 Comparison of the IAST based separation potential for C_3H_4/C_3H_6 mixtures in different proportions in ZNU-2 and reported top performing MOFs.

Table S18 Comparison of the reported materials on C_3H_4 adsorption enthalpy (Q_{st}).

	Q_{st} (kJ/mol)	Ref
ELM-12	60.6	[1]
ZU-62	121.9/71.0	[14, 11]
SIFSIX-2-Cu-i	82.0/46.0	[5,14]
ZJUT-1	33.6	[2]
NKMOF-11	85.0	[3]
JXNU-6	40.0	[4]
SIFSIX-1-Cu	37.2	[5]
SIFSIX-3-Ni	68.0	[5]
UTSA-200	55.3	[6]
NKMOF-1-Ni	65.1	[11]
NKMOF-1-Cu	67.2	[11]
Co-gallate	82.1	[10]
Mg-gallate	66.8	[10]
Ni-gallate	84.4	[10]
Ca-based MOF	55.4	[11]
Cu-BTC	46.0	[16]
FJI-W1	61.7	[35]
ZNU-2-Si	43.3	this work
ZNU-2-Ti	43.0	[1]/this work
ZNU-2-Nb	41.6	this work

IV Crystallography based DFT calculation

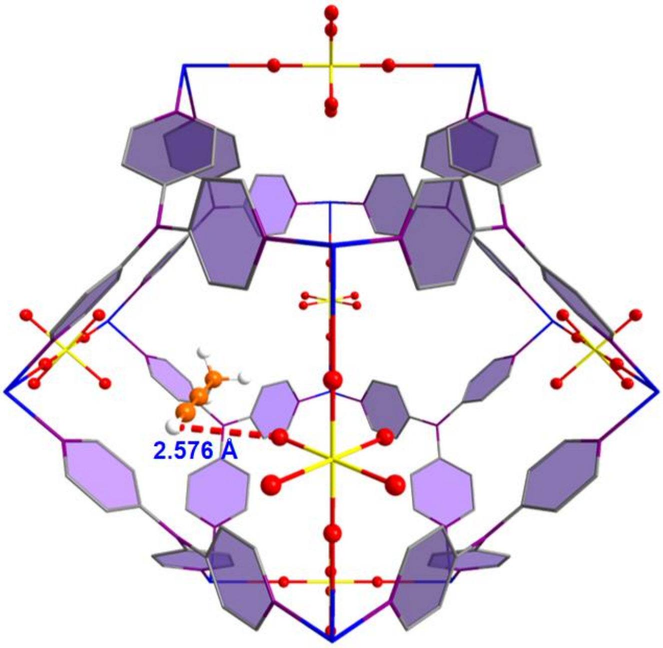

ΔE = -39.35 kJ/mol

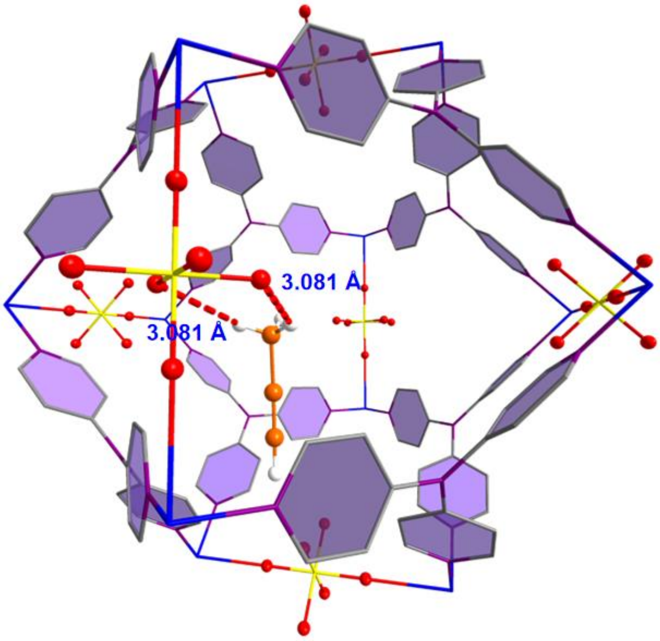

ΔE = -35.87 kJ/mol

Fig. S50 DFT calculated interaction energy of ZNU-2-Si and C_3H_4 with two similar configurations. The one with alkynyl C-H end closer to the interlaced channel (above) display higher binding energy (-39.35 kJ/mol) than the other one (below).

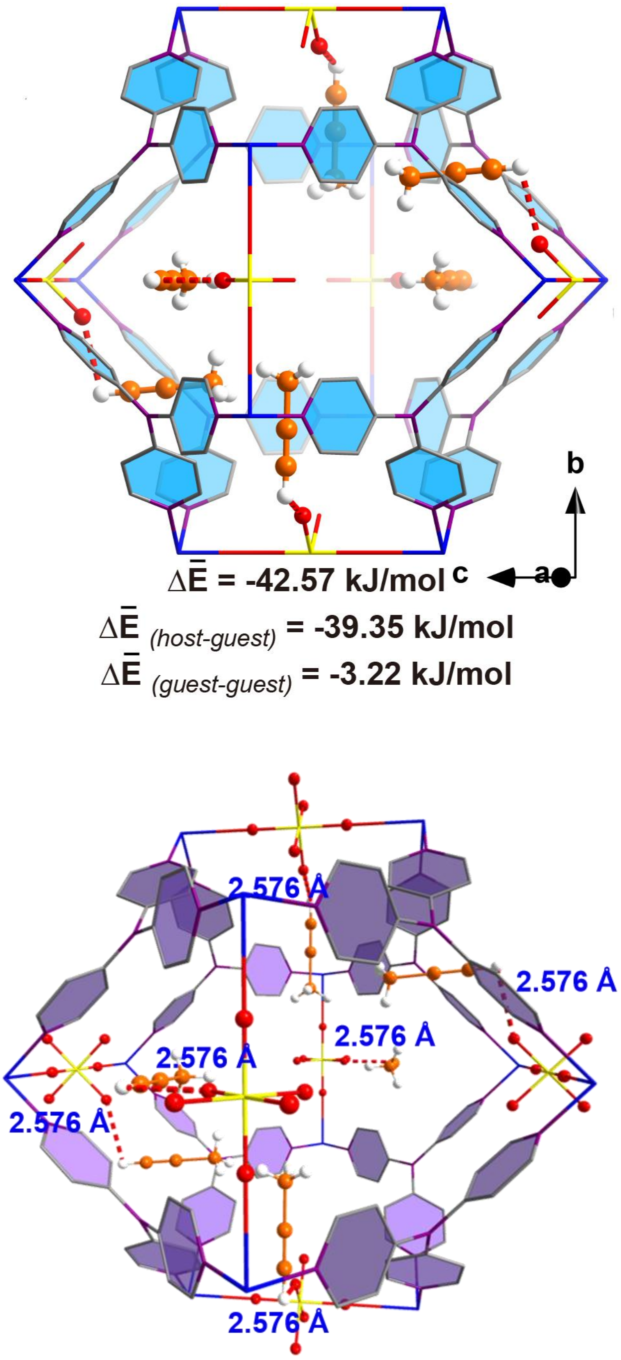

Fig. S51 DFT calculated interaction energy of ZNU-2-Si and gas molecules under the situation that six C_3H_4 located in a cage.

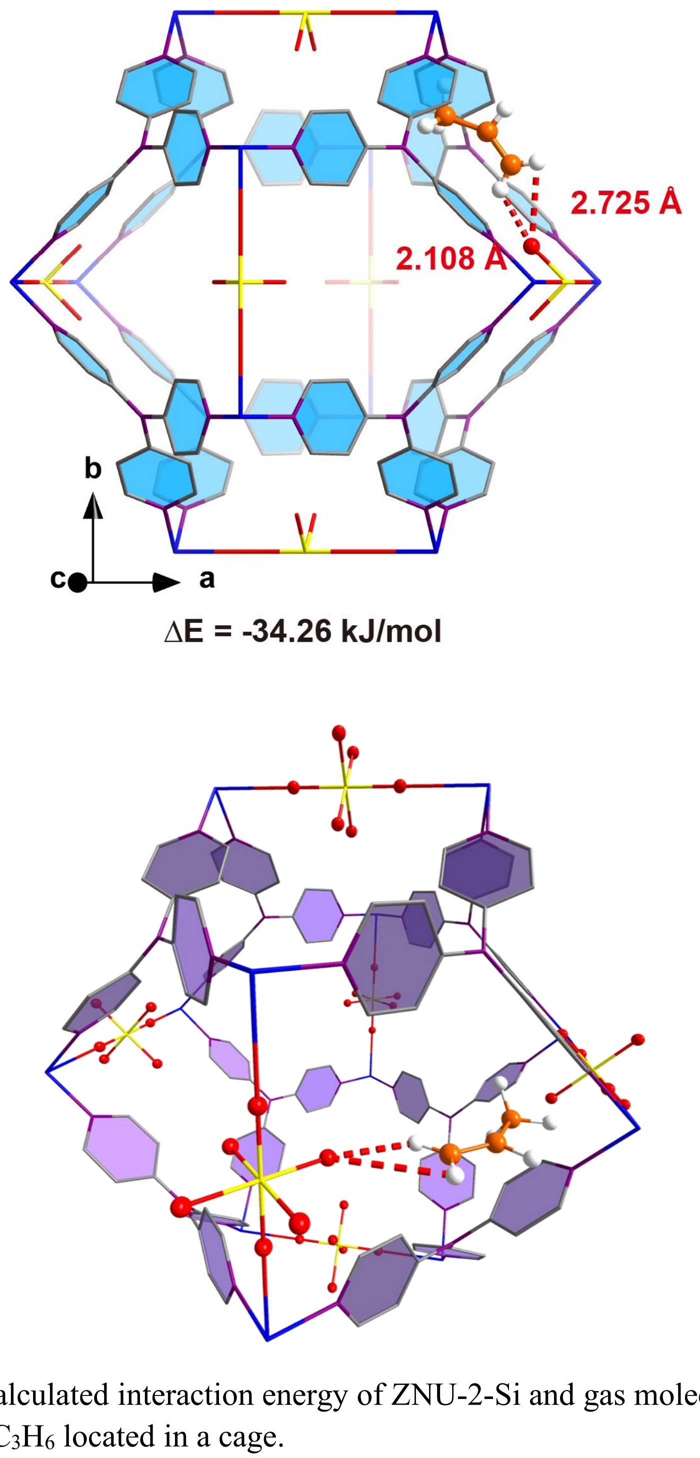

Fig. S52 DFT calculated interaction energy of ZNU-2-Si and gas molecules under the situation that a C_3H_6 located in a cage.

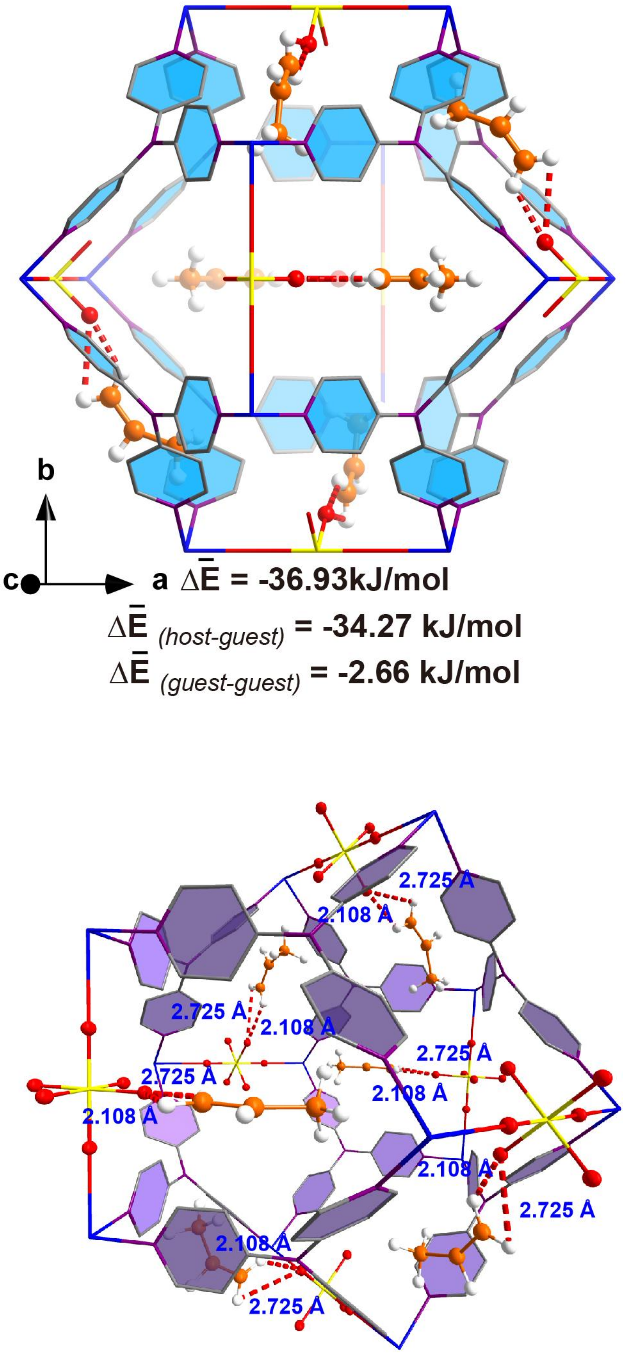

Fig. S53 DFT calculated interaction energy of ZNU-2-Si and gas molecules under the situation that six C_3H_6 located in a cage.

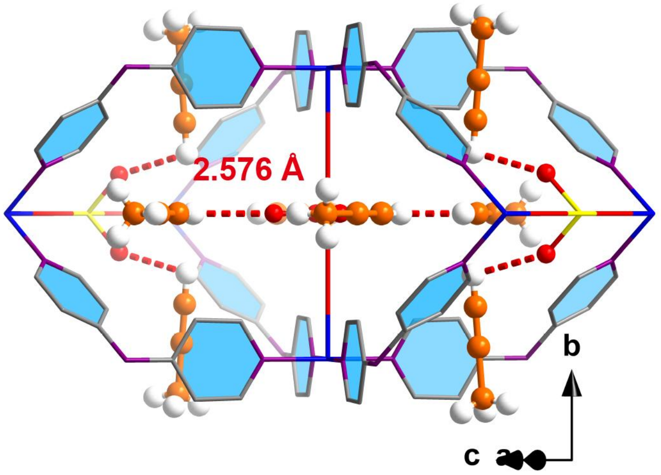

$\Delta\bar{E}$ = -44.25 kJ/mol

$\Delta\bar{E}$ *(host-guest)* = -39.36 kJ/mol

$\Delta\bar{E}$ *(guest-guest)* = -4.89 kJ/mol

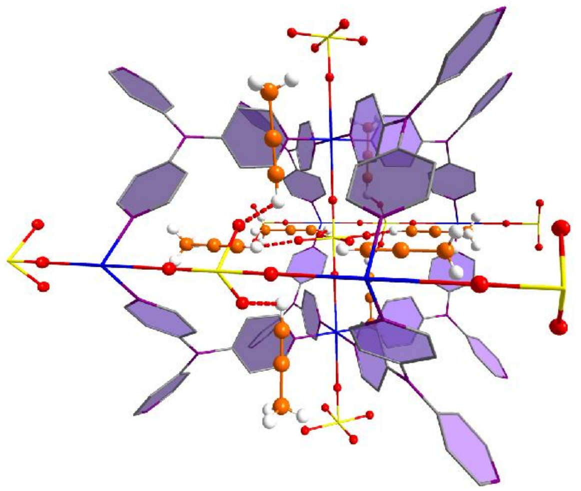

Fig. S54 DFT calculated interaction energy of ZNU-2-Si and gas molecules under the situation that 8 C_3H_4 molecules located near two neighbouring two interlaced channels.

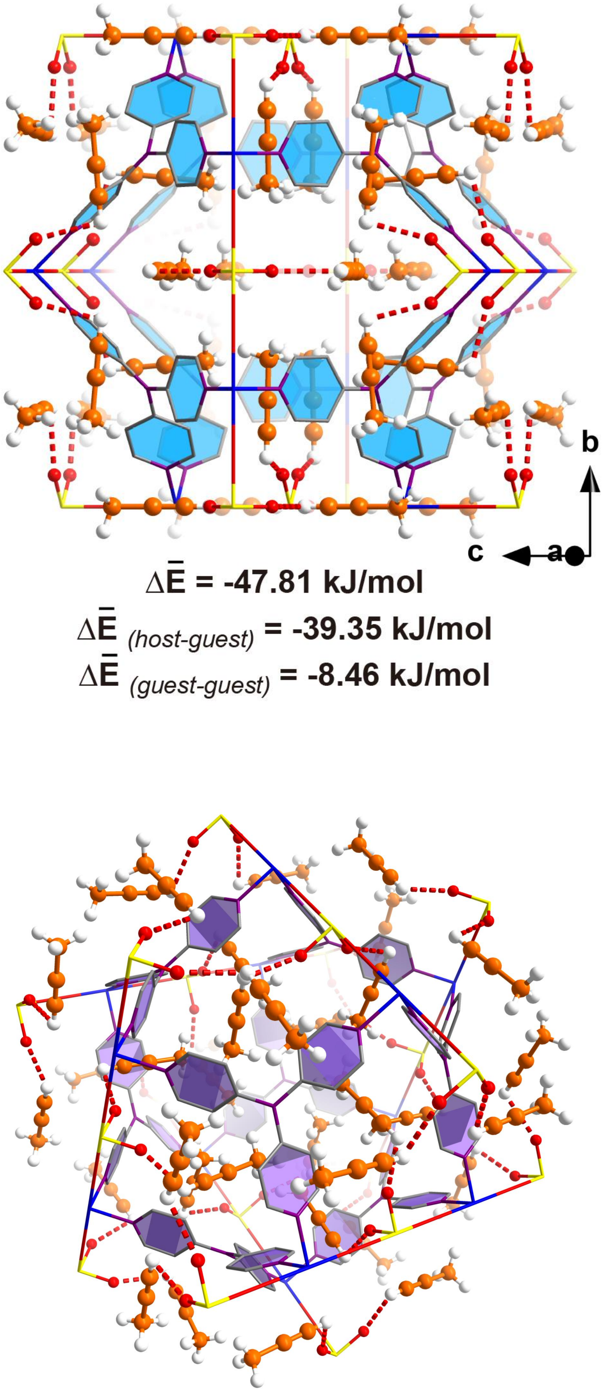

Fig. S55 DFT calculated interaction energy of ZNU-2-Si and gas molecules under the situation that 8 C_3H_4 molecules located near two neighbouring two interlaced channels.

V GCMC simulation based DFT calculation

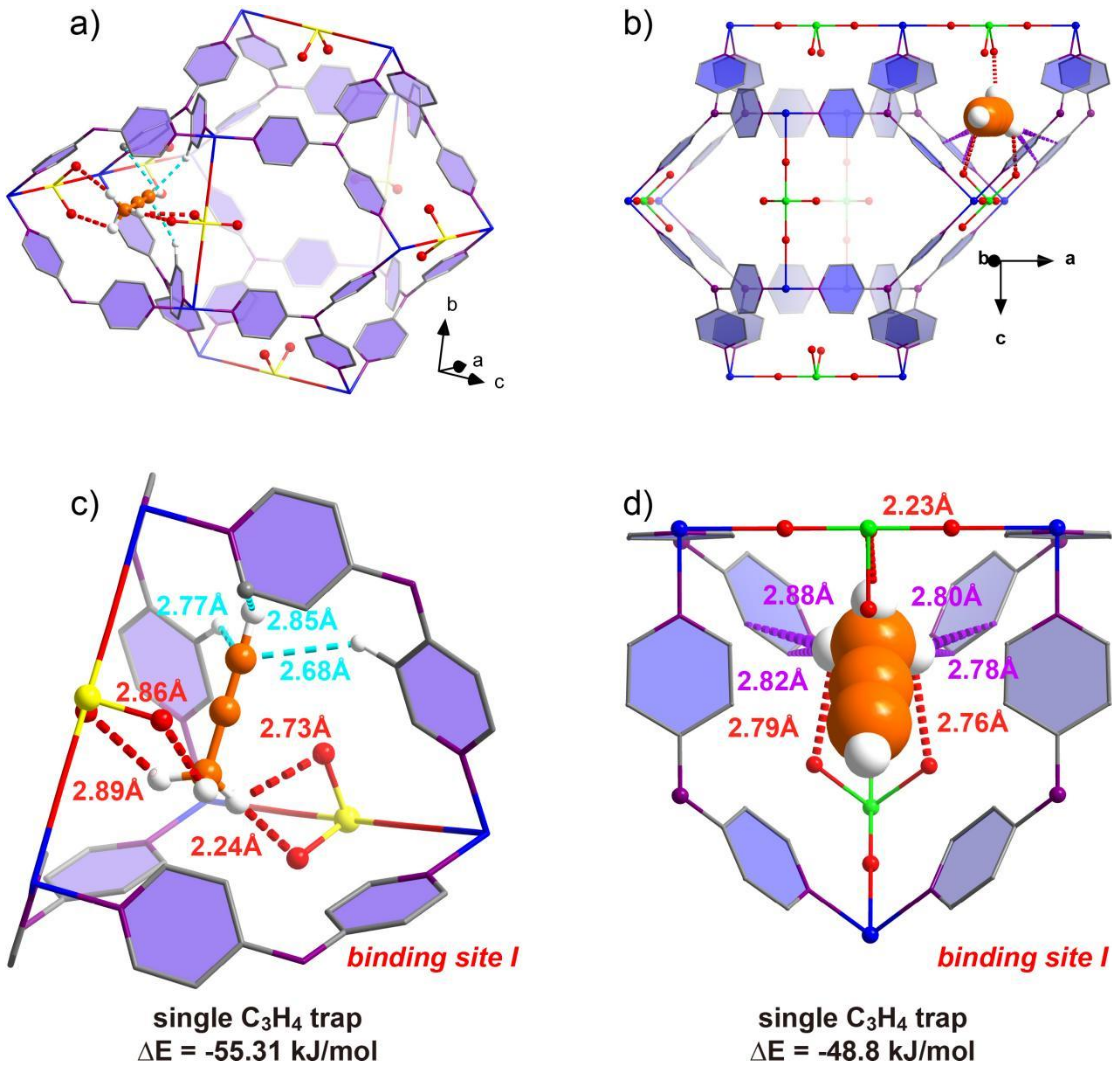

Fig. S56 The DFT-D optimized adsorption configuration of C_3H_4 in the first binding site in the channel among four cages (Left: ZNU-2-Si (a, c); Right: ZNU-2-Ti (b, d)).

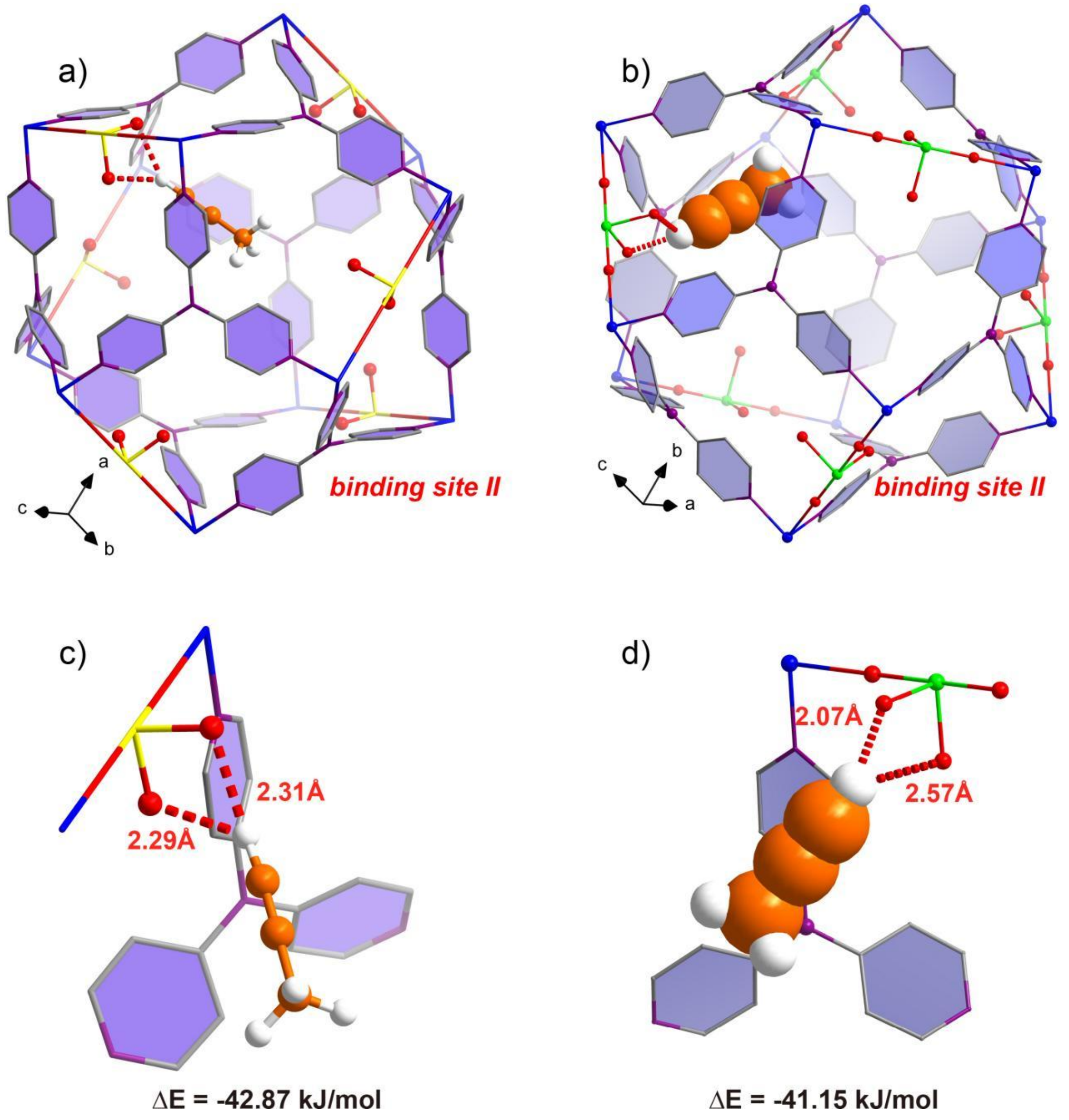

Fig. S57 The DFT-D optimized adsorption configuration of one C_3H_4 in the second binding site in the cage (Left: ZNU-2-Si (a,c); Right: ZNU-2-Ti (b, d)).

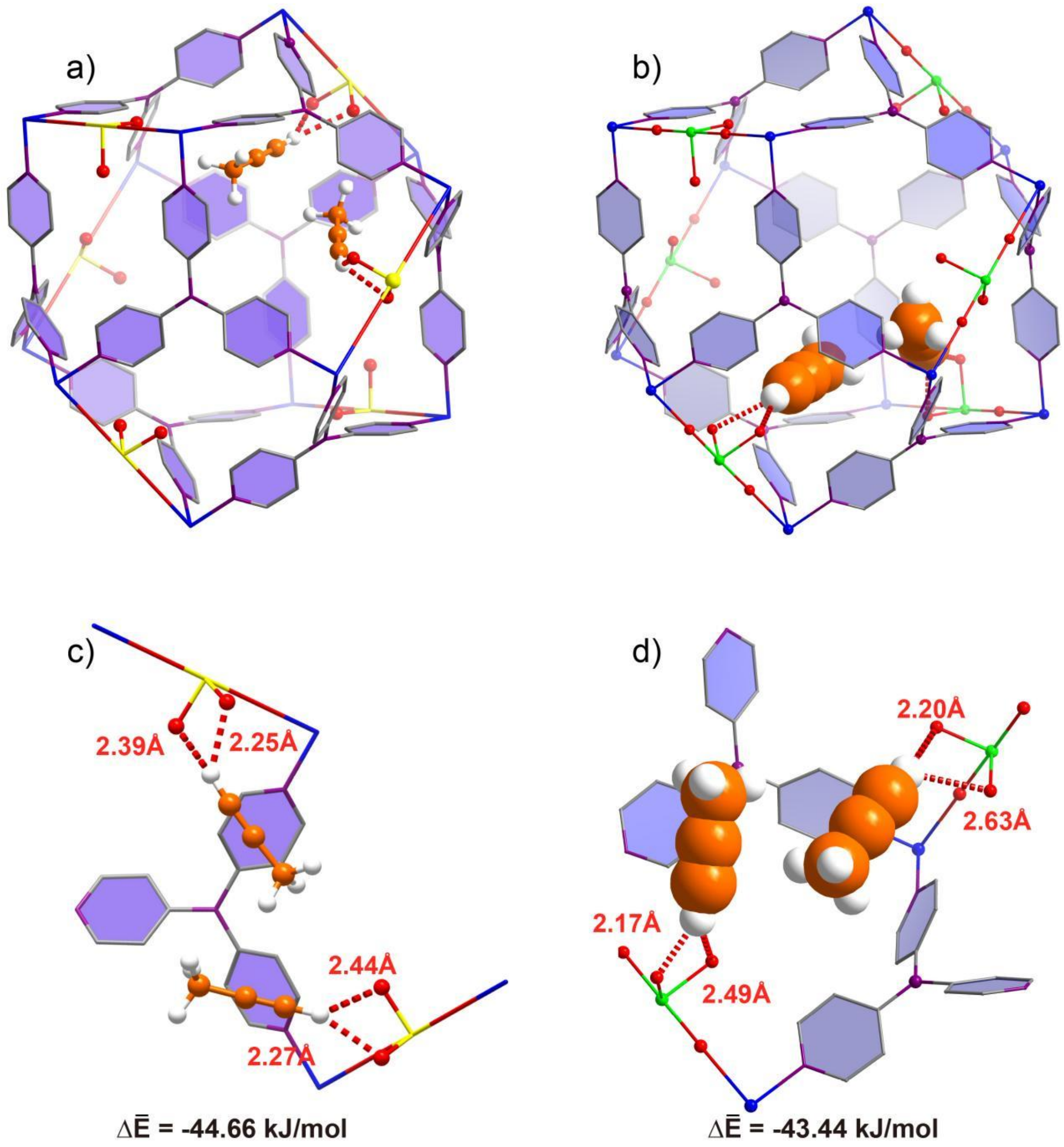

Fig. S58 The DFT-D optimized adsorption configuration of two C_3H_4 molecules in the cage (Left: ZNU-2-Si (a,c); Right: ZNU-2-Ti (b, d)).

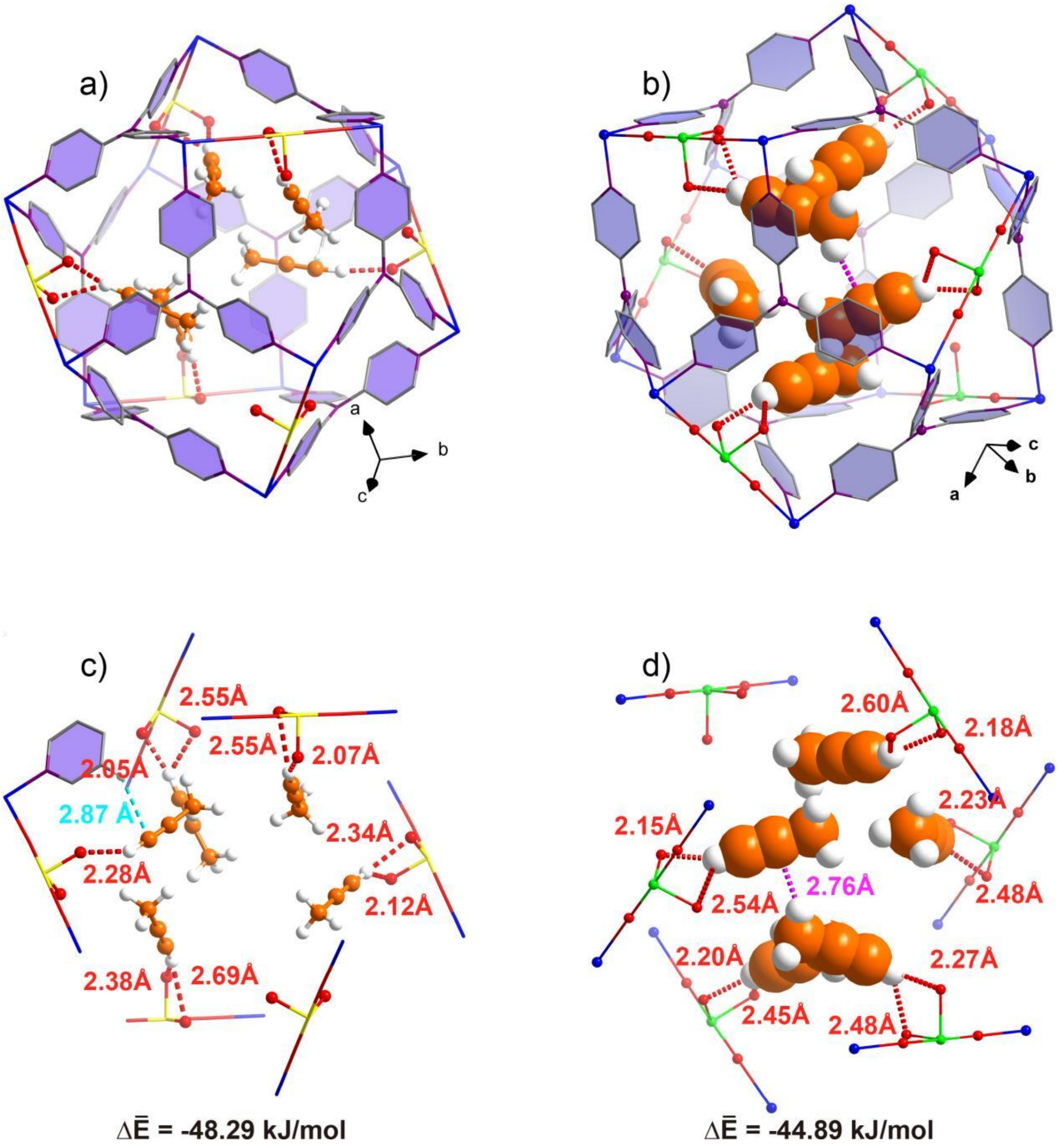

Fig. S59 The DFT-D optimized adsorption configuration of five C_3H_4 molecules in the cage (Left: ZNU-2-Si (a,c); Right: ZNU-2-Ti (b, d)).

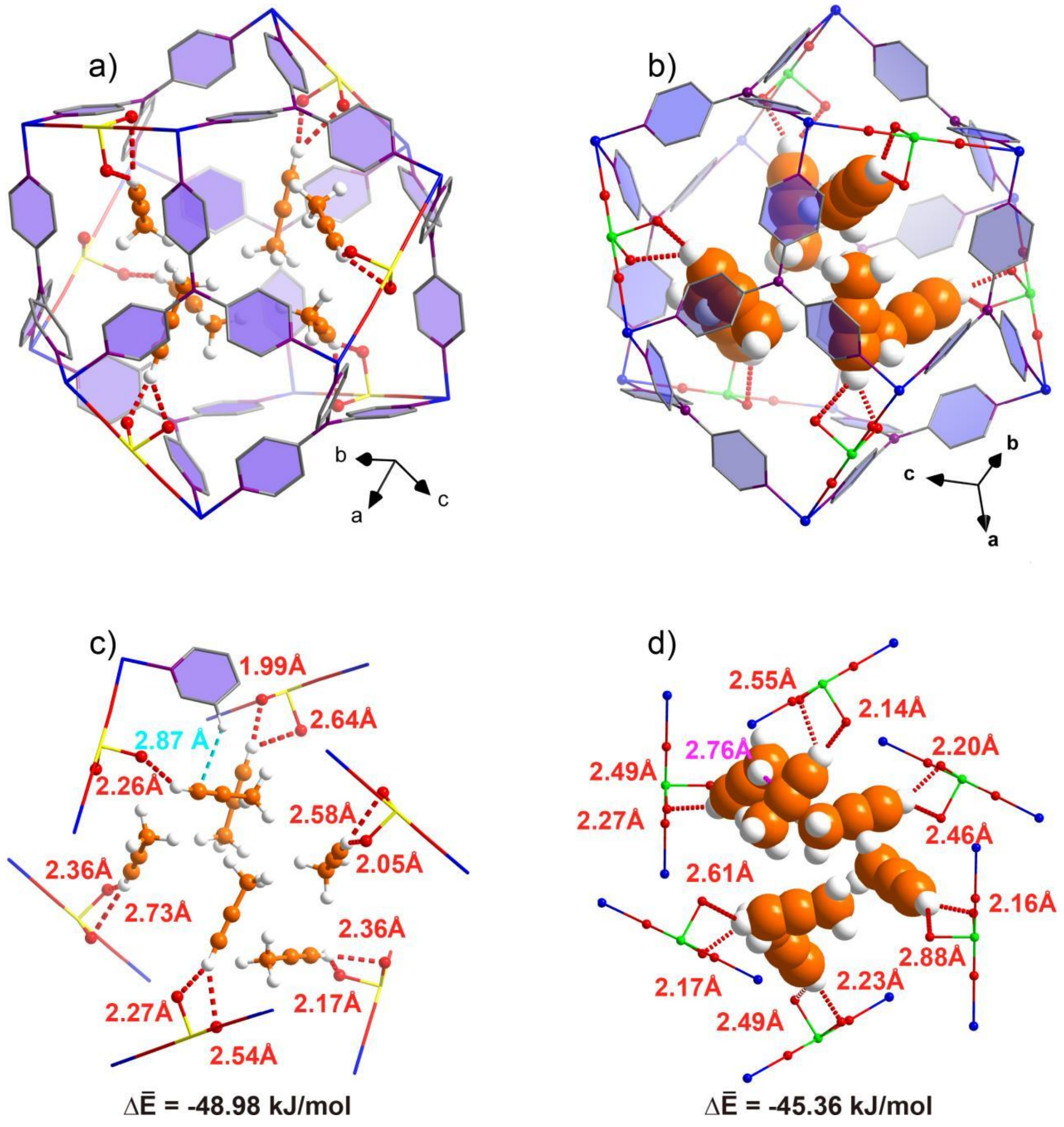

Fig. S60 The DFT-D optimized adsorption configuration of six C_3H_4 molecules in the cage (Left: ZNU-2-Si (a,c); Right: ZNU-2-Ti (b, d)).

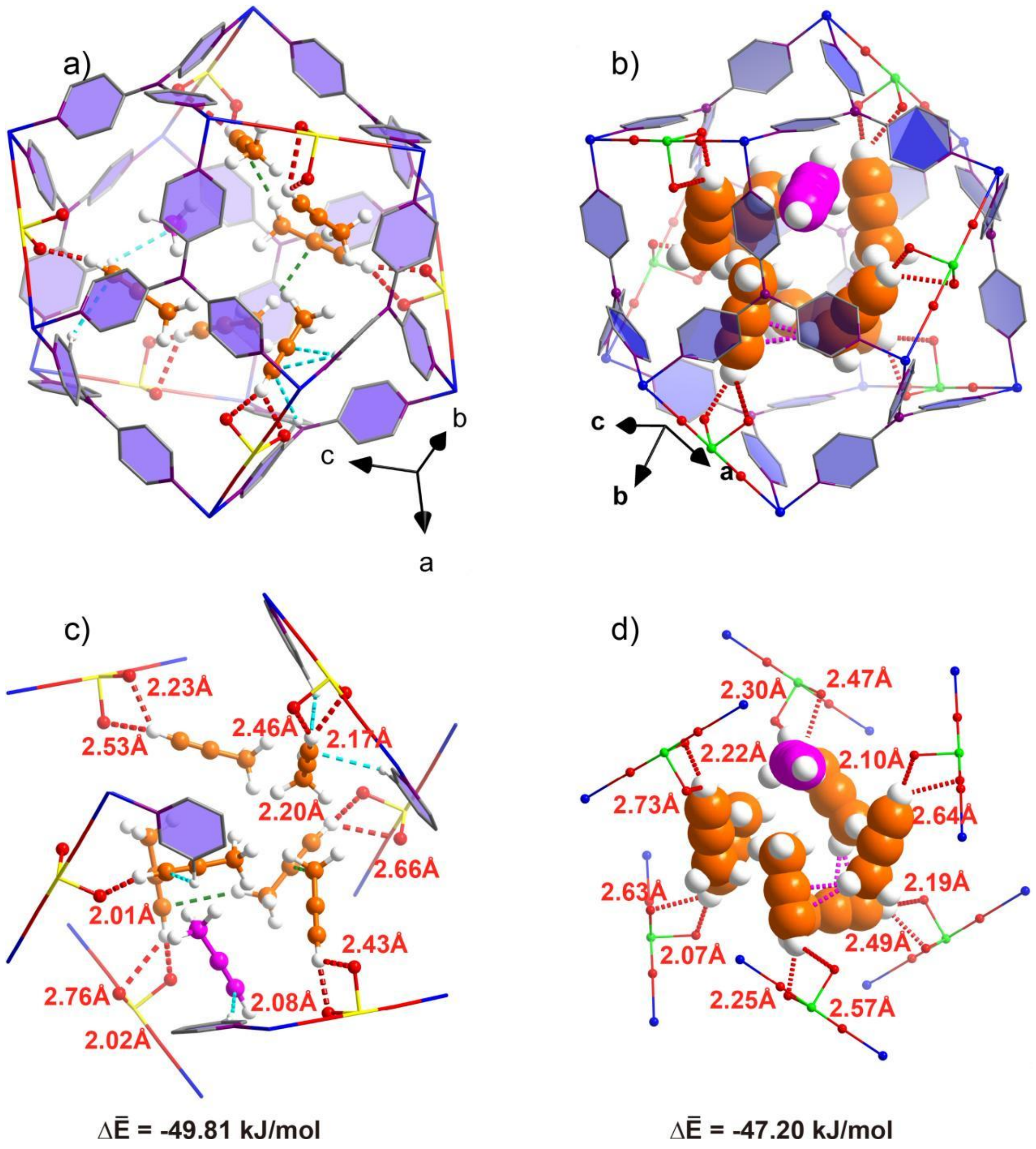

Fig. S61 The DFT-D optimized adsorption configuration of seven C_3H_4 molecules in the cage (Left: ZNU-2-Si (a,c); Right: ZNU-2-Ti (b, d)).

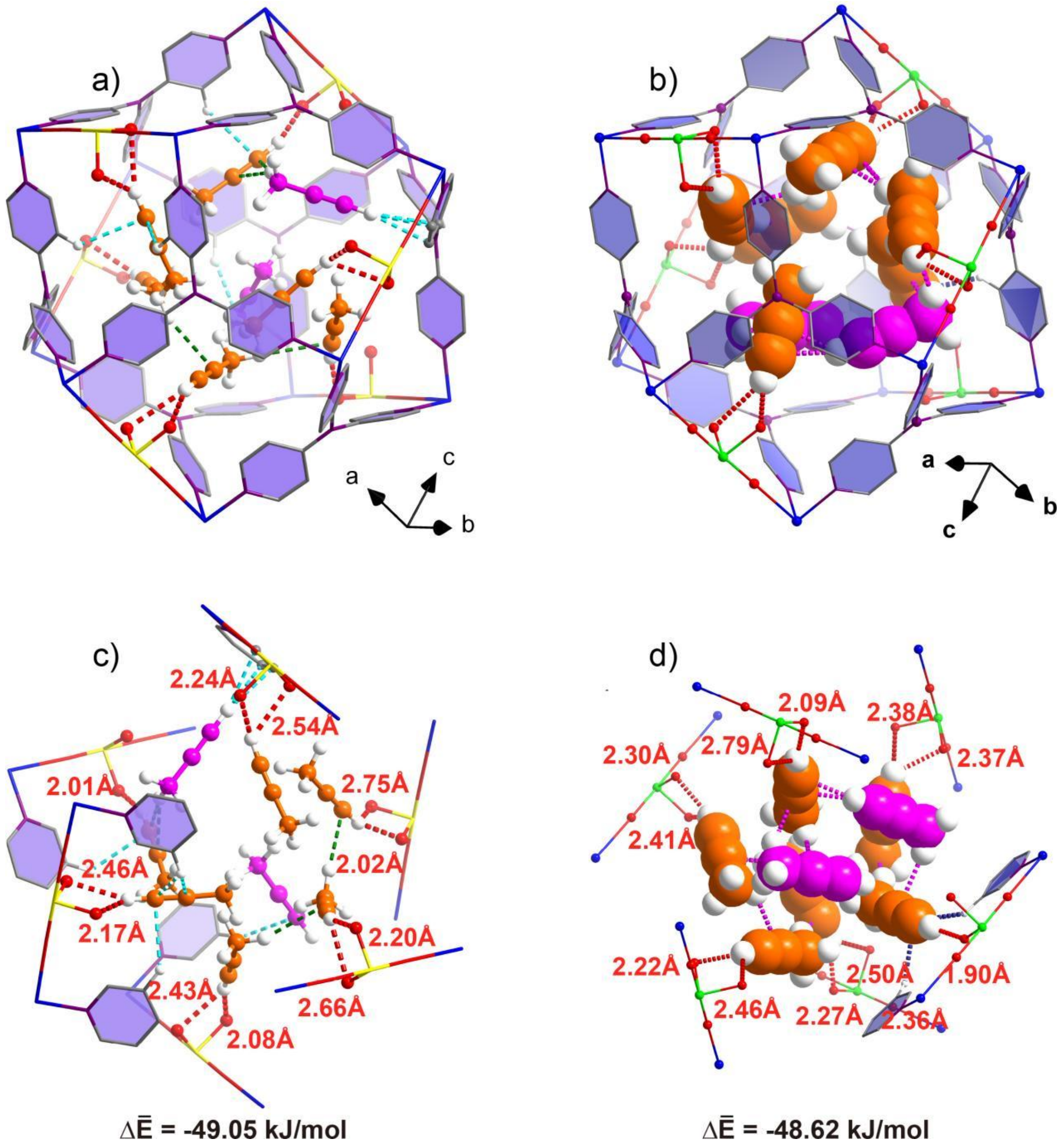

Fig. S62 The DFT-D optimized adsorption configuration of eight C_3H_4 molecules in the cage (Left: ZNU-2-Si (a,c); Right: ZNU-2-Ti (b, d)).

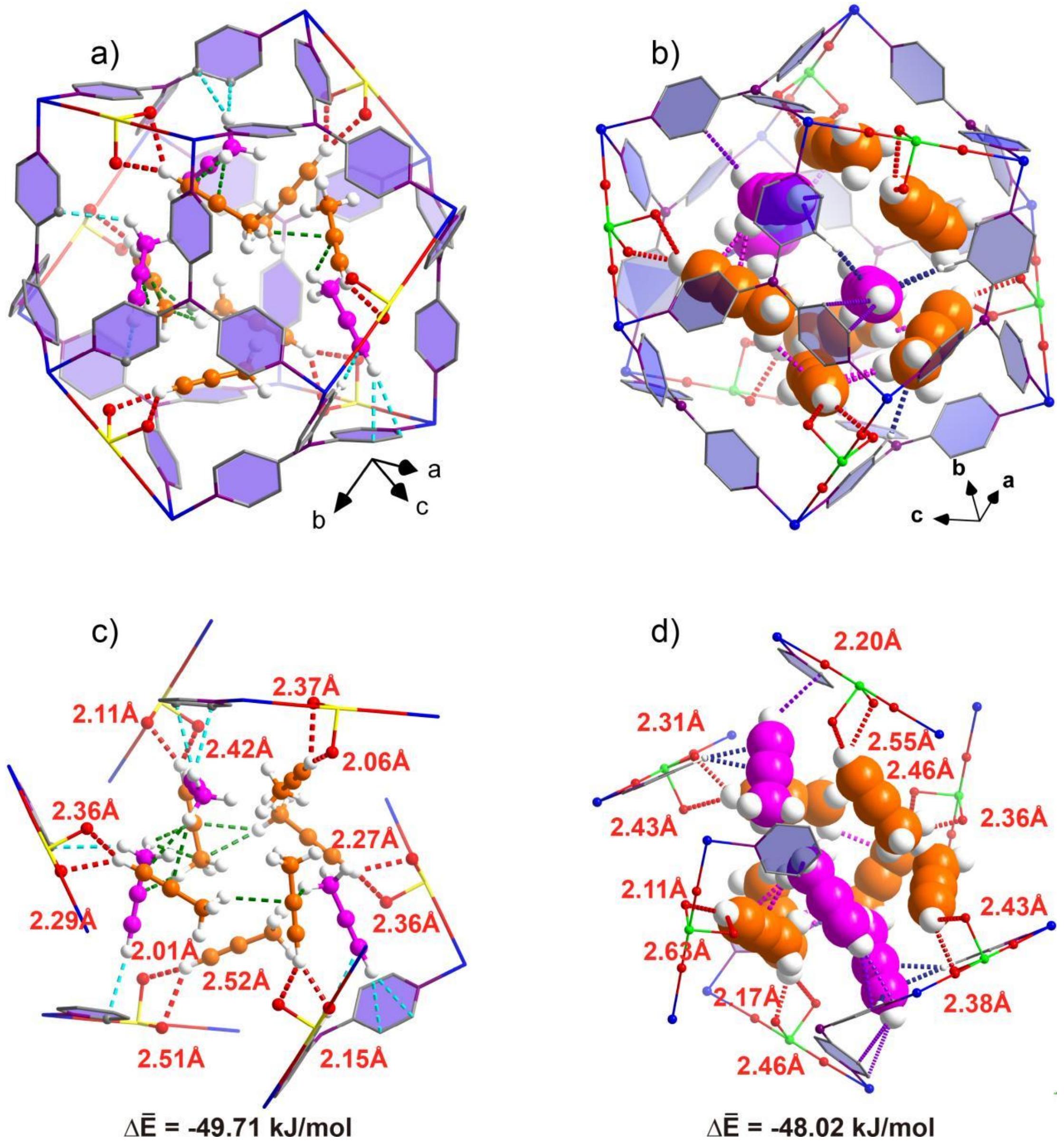

Fig. S63 The DFT-D optimized adsorption configuration of nine C_3H_4 molecules in the cage (Left: ZNU-2-Si (a,c); Right: ZNU-2-Ti (b, d)).

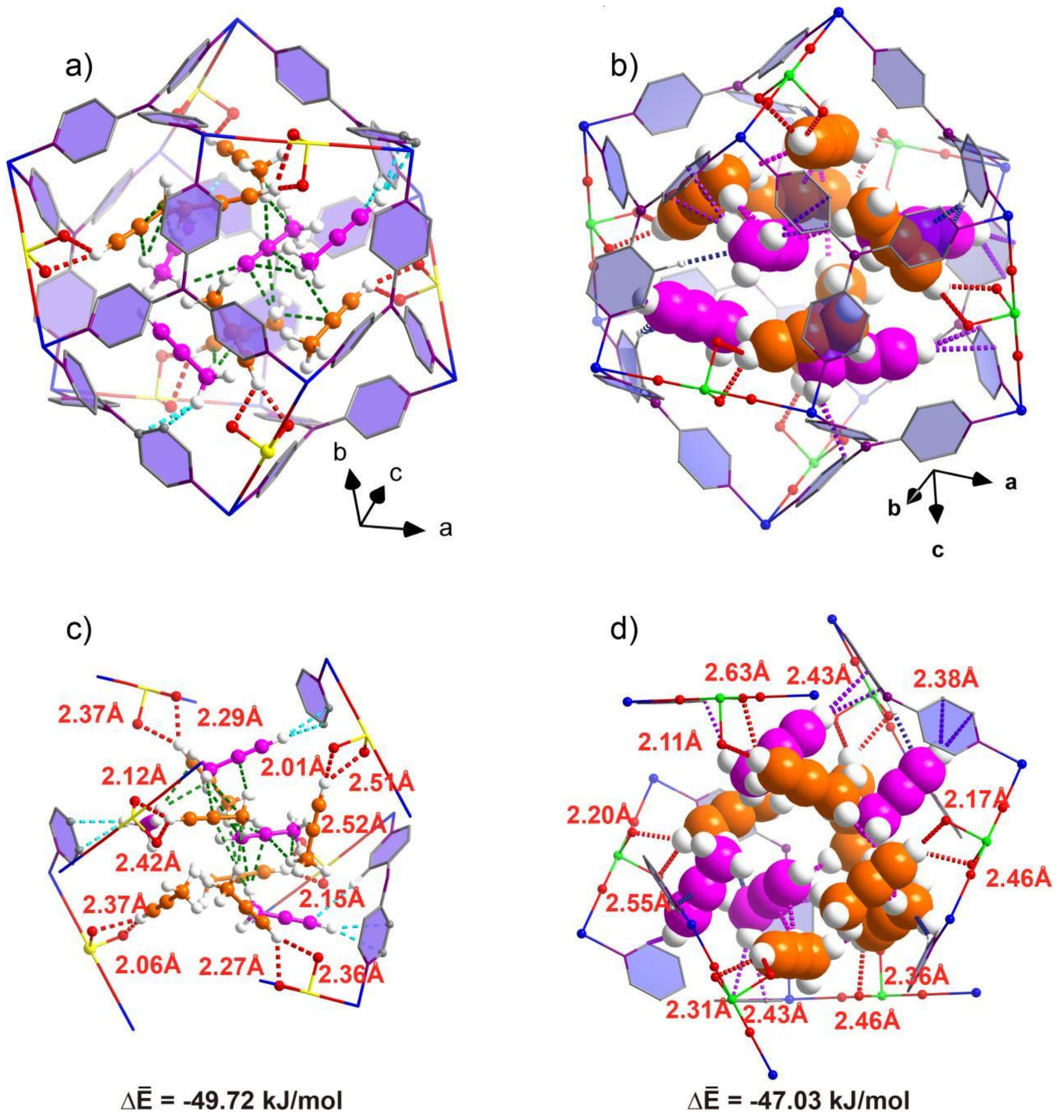

Fig. S64 The DFT-D optimized adsorption configuration of ten C_3H_4 molecules in the cage (Left: ZNU-2-Si (a,c); Right: ZNU-2-Ti (b, d)).

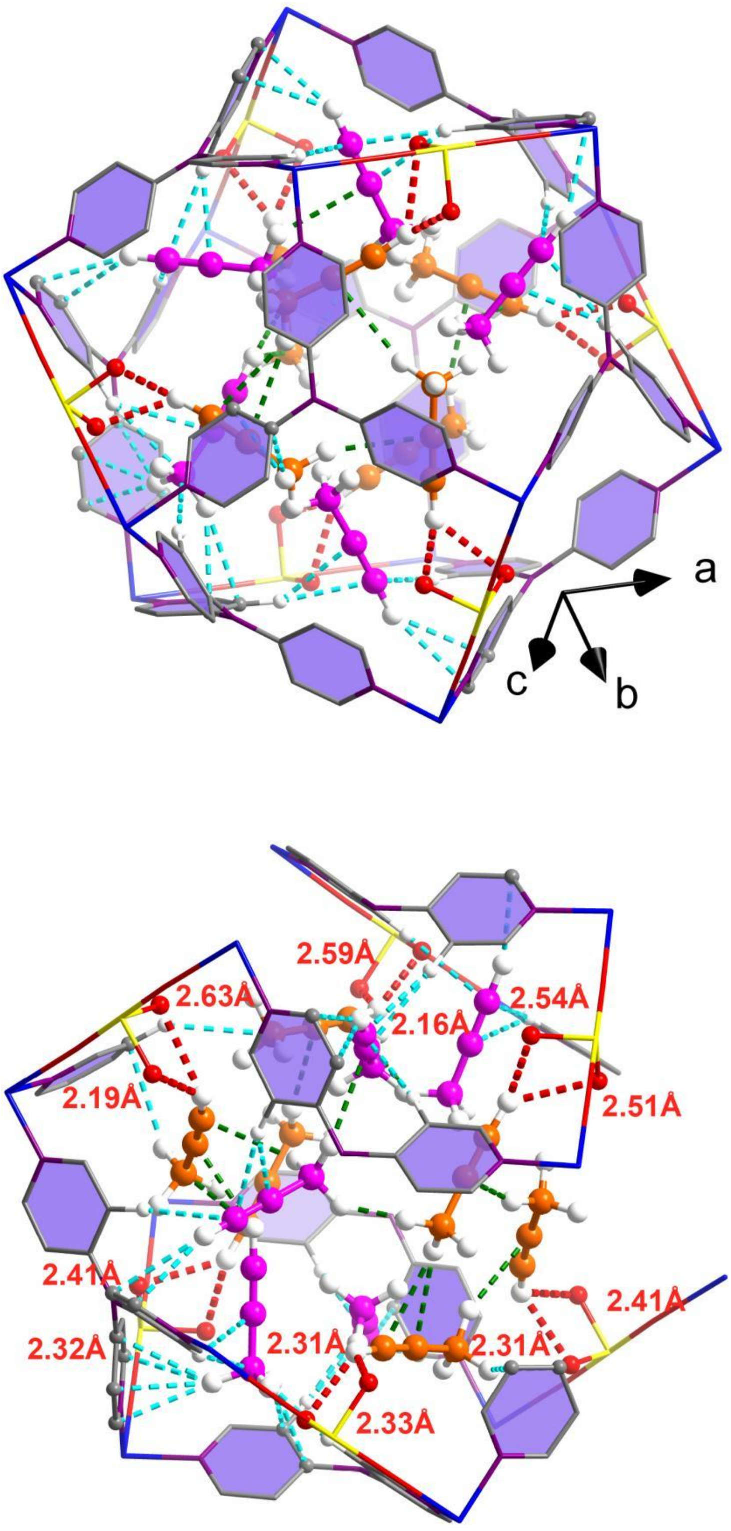

$\Delta\bar{E}$ = -49.63 kJ/mol

Fig. S65 The DFT-D optimized adsorption configuration of eleven C_3H_4 molecules in the cage of ZNU-2-Si.

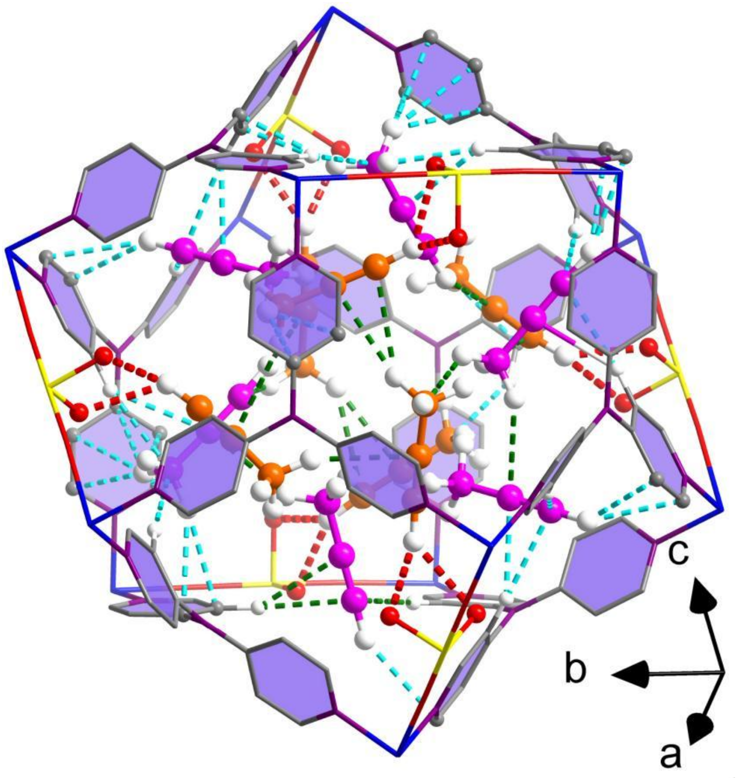

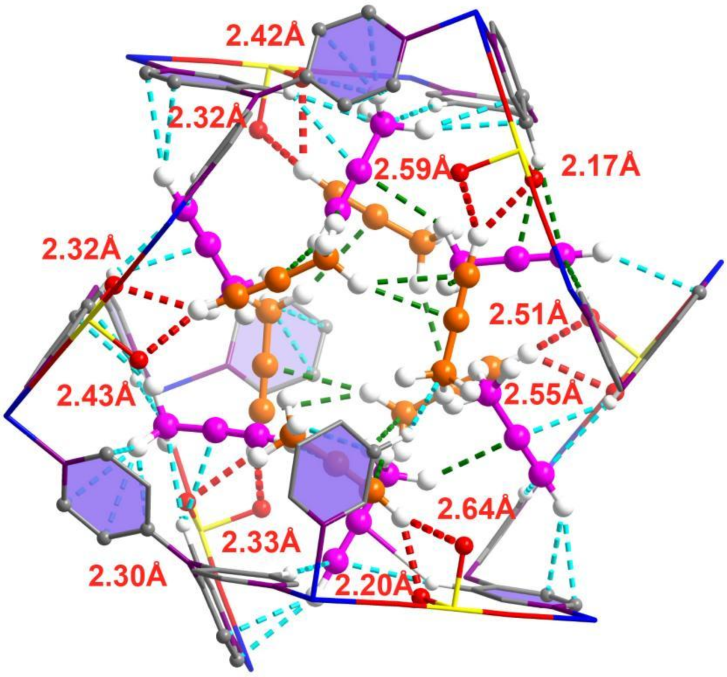

Fig. S66 The DFT-D optimized adsorption configuration of twelve C_3H_4 molecules in the cage of ZNU-2-Si.

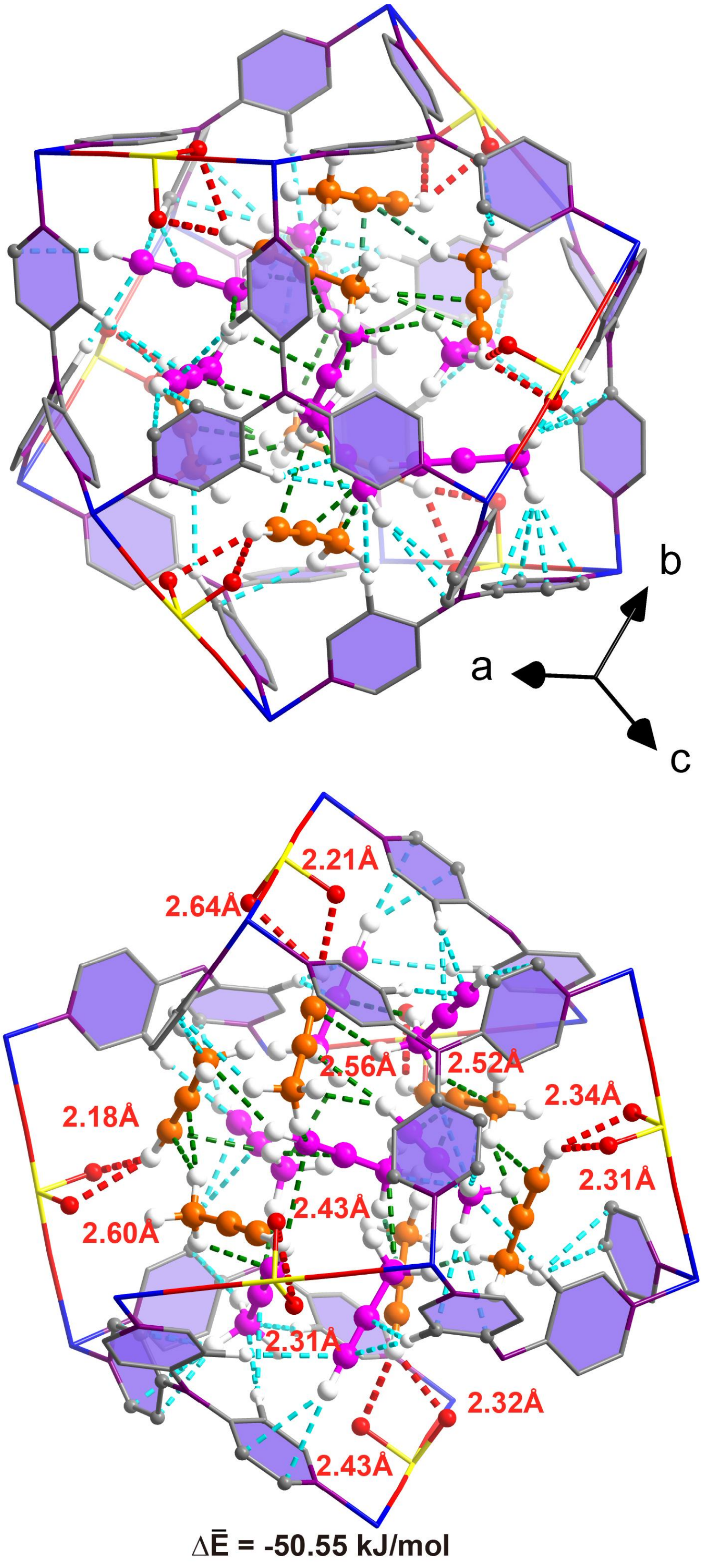

Fig. S67 The DFT-D optimized adsorption configuration of thirteen C_3H_4 molecules in the cage of ZNU-2-Si.

Table S19 Comparison of the average binding energy of C_3H_4 molecules absorbed in ZNU-2-Si and ZNU-2-Ti.

**Number of C_3H_4 molecular**	**Average binding energy (kJ/mol)**	
	**ZNU-2-Si**	**ZNU-2-Ti**
1 (binding site I)	-55.31	-48.78
1 (binding site II)	-42.87	-41.15
2	-44.66	-43.44
5	-48.29	-44.88
6	-48.98	-45.35
7	-49.81	-47.20
8	-49.05	-48.62
9	-49.71	-48.01
10	-49.72	-47.03
11	-49.63	-
12	-49.86	-
13	-50.55	-

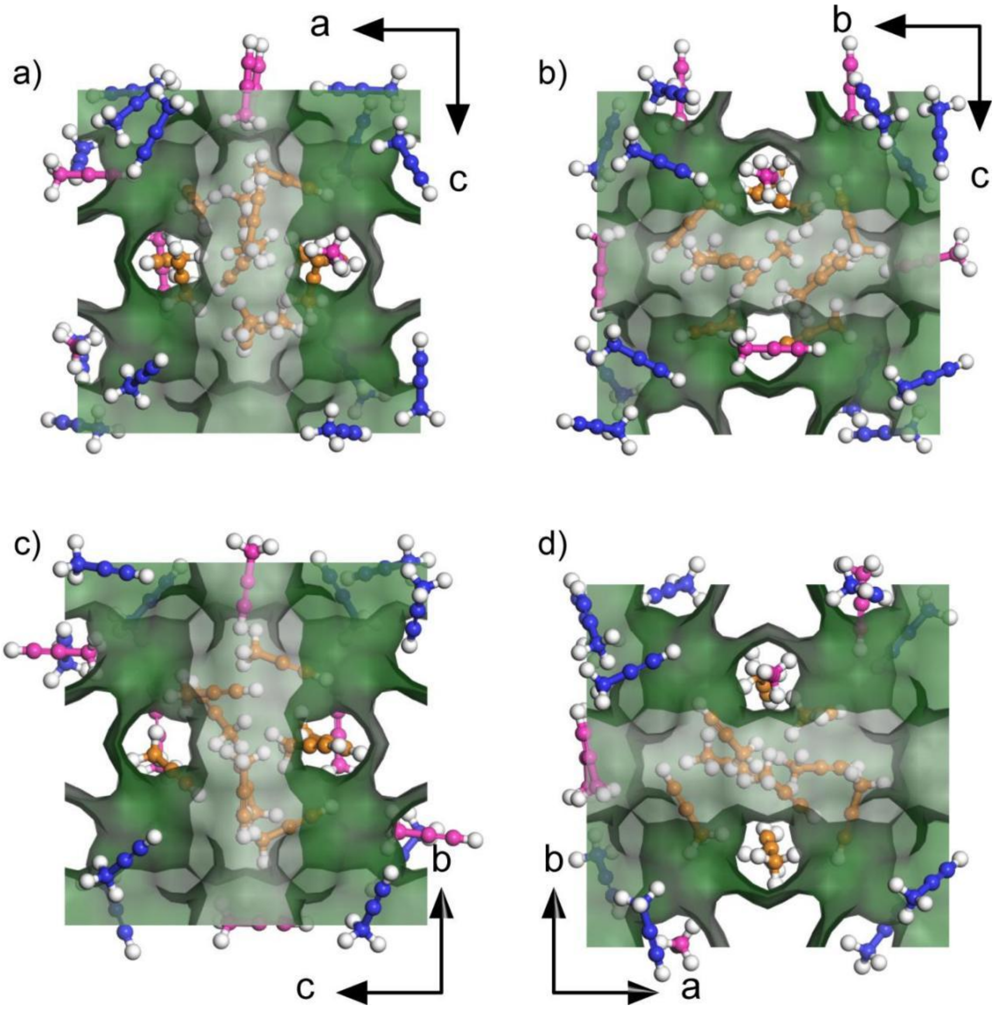

Fig. S68 The GCMC optimized adsorption configuration of saturated C_3H_4 molecules in the unit cell of ZNU-2-Si (a, b) and ZNU-2-Ti (c, d)

An unit cell is consisted of six channels and two cages (there is a complete cage in the middle of the cell and eight corners from eight different cages; each corner accounts for one-eighth of a cage). Pink C_3H_4 molecules: located in the channel (the first binding site); Orange C_3H_4 molecules: located in the cage (the second binding site); Blue C_3H_4 molecules: in the other corner-cages (the second binding site).

The simulated uptake capacity of C_3H_4 molecules in an unit cell in ZNU-2-Si is 30, equals to 5 C_3H_4 for each SiF_6^{2-}, while in ZNU-2-Ti is 25, equals to 4.17 C_3H_4 for each TiF_6^{2-}. The results calculated from GCMC are approximate to the experimental C_3H_4/MFSIX ratio values of 4.54/4.26 (Fig. S21).

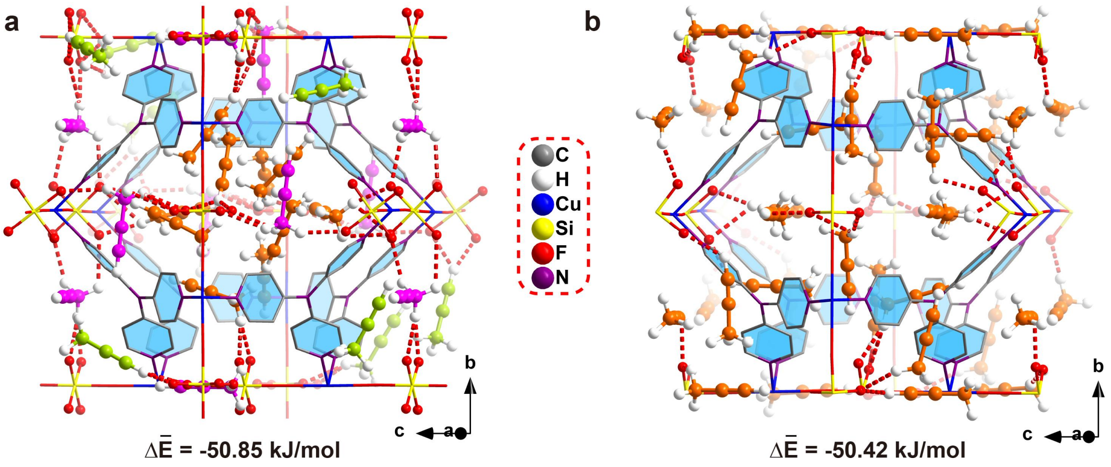

Fig. S69. The GCMC optimized adsorption configuration of 24 C_3H_4 molecules in the unit cell of ZNU-2-Si.(a) a holistic view; (b) Viewed around a cage; (c-e) Viewed around the interlaced channel.

As described above, An unit cell is consisted of six channels and two cages (there is a complete cage in the middle of the cell and eight corners from eight different cages; each corner accounts for one-eighth of a cage). Pink C_3H_4 molecules: located in the interlaced channel (the first binding site); Orange C_3H_4 molecules: located in the cage (the second binding site); Green C_3H_4 molecules: in the other corner-cages (the second binding site).

VI MD simulation

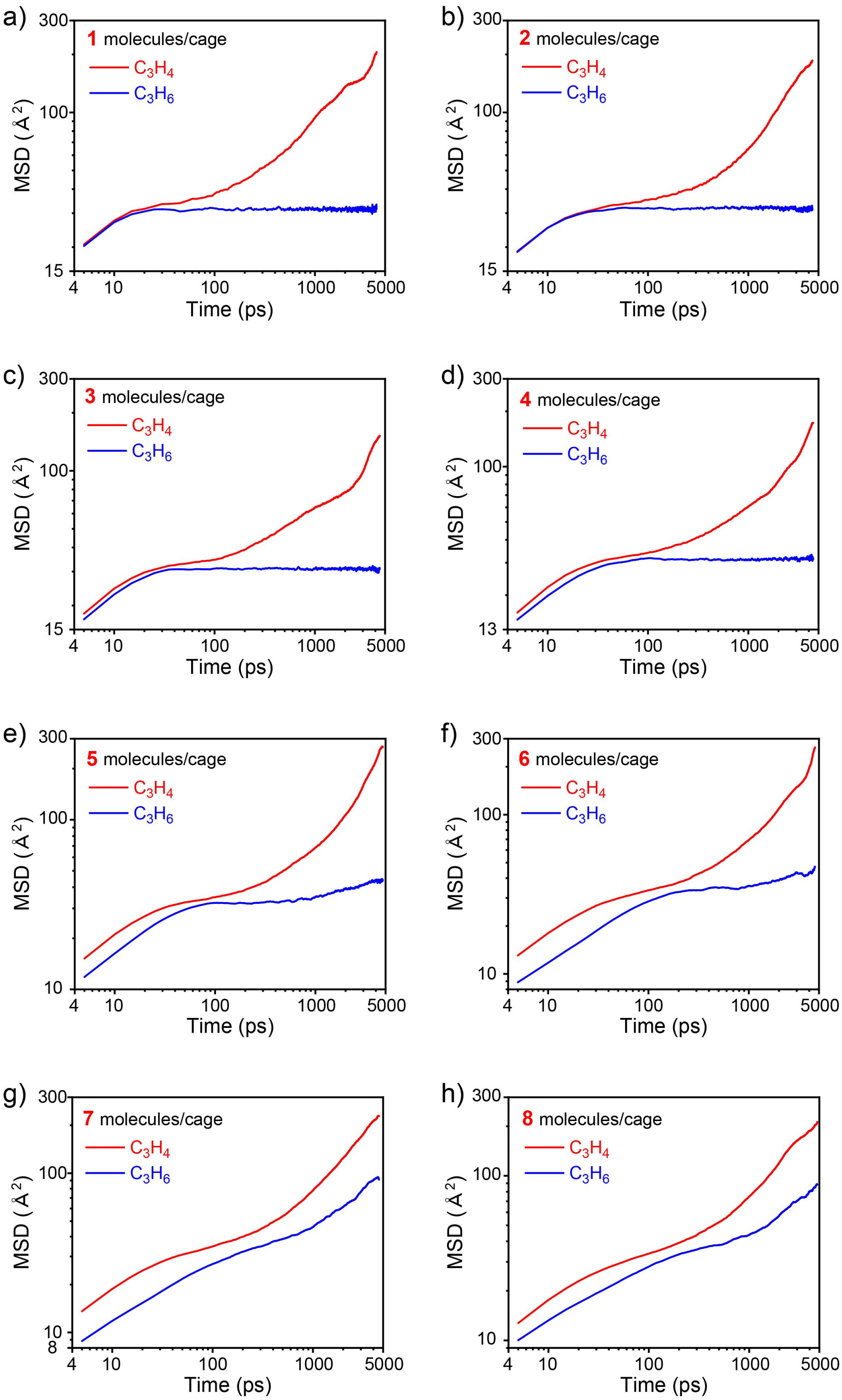

Fig. S70 MD simulations. MSD plot of C_3H_4 and C_3H_6 molecules in ZNU-2-Si with 1-8 molecules in a single cage.

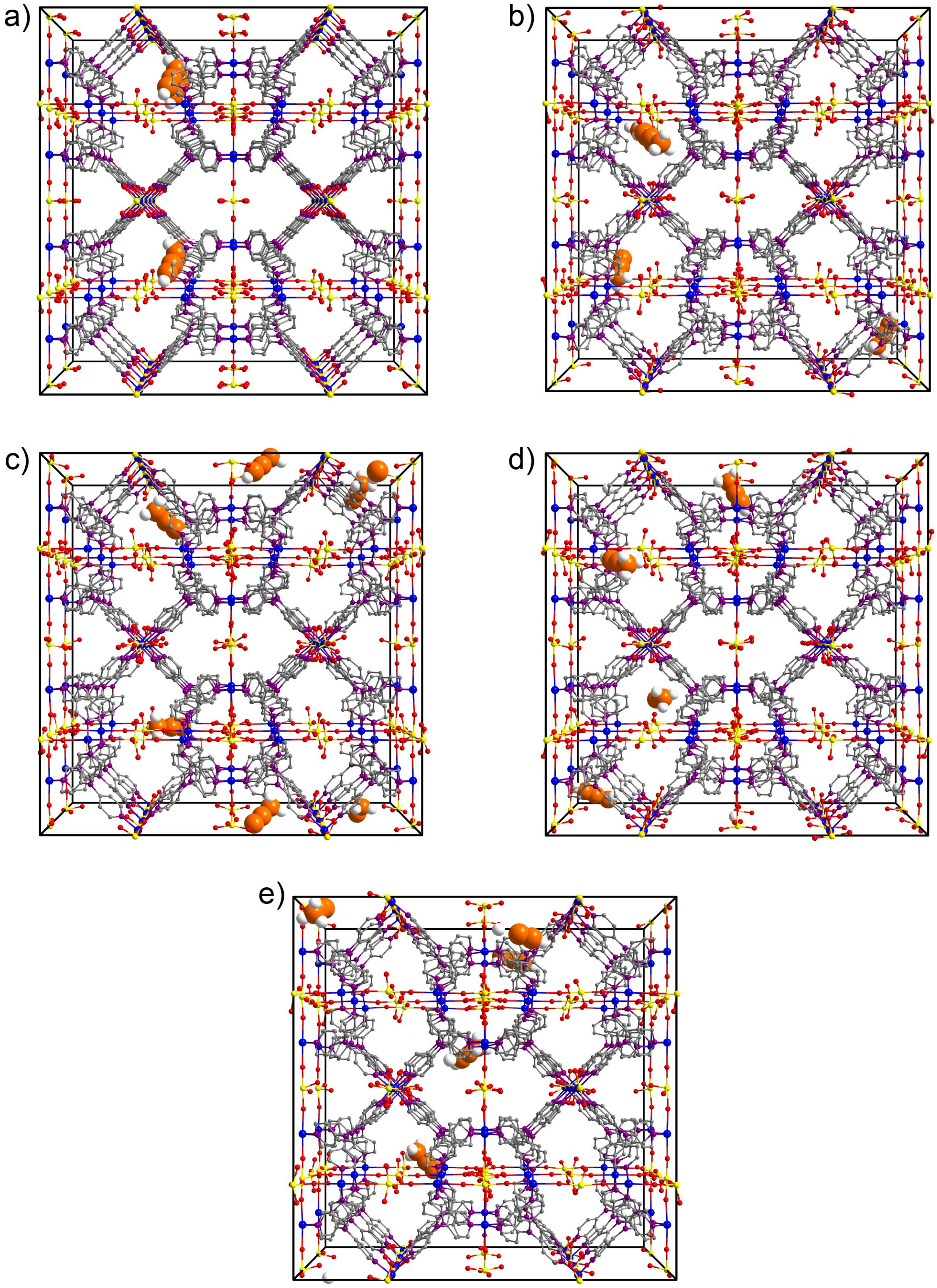

Fig. S71 MD simulations. Snapshots of MD simulation of C_3H_4 molecules in (a) 0, (b) 1250, (c) 2500, (d) 3750 and (e) 5000 ps under the loading of 1 C_3H_4/cage.

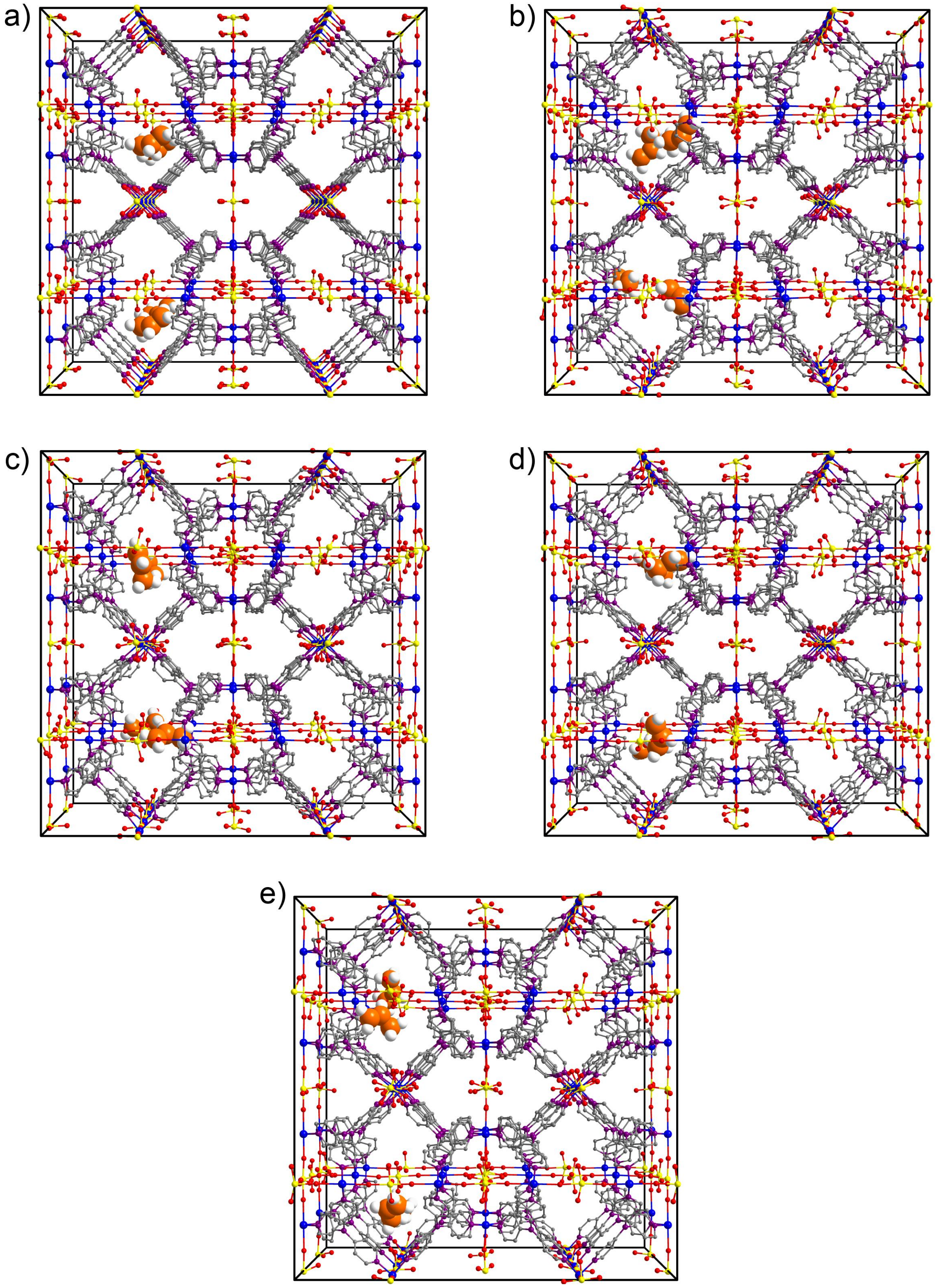

Fig. S72 MD simulations. Snapshots of MD simulation of C_3H_6 molecules in (a) 0, (b) 1250, (c) 2500, (d) 3750 and (e) 5000 ps, under the loading of 1 C_3H_6/cage.

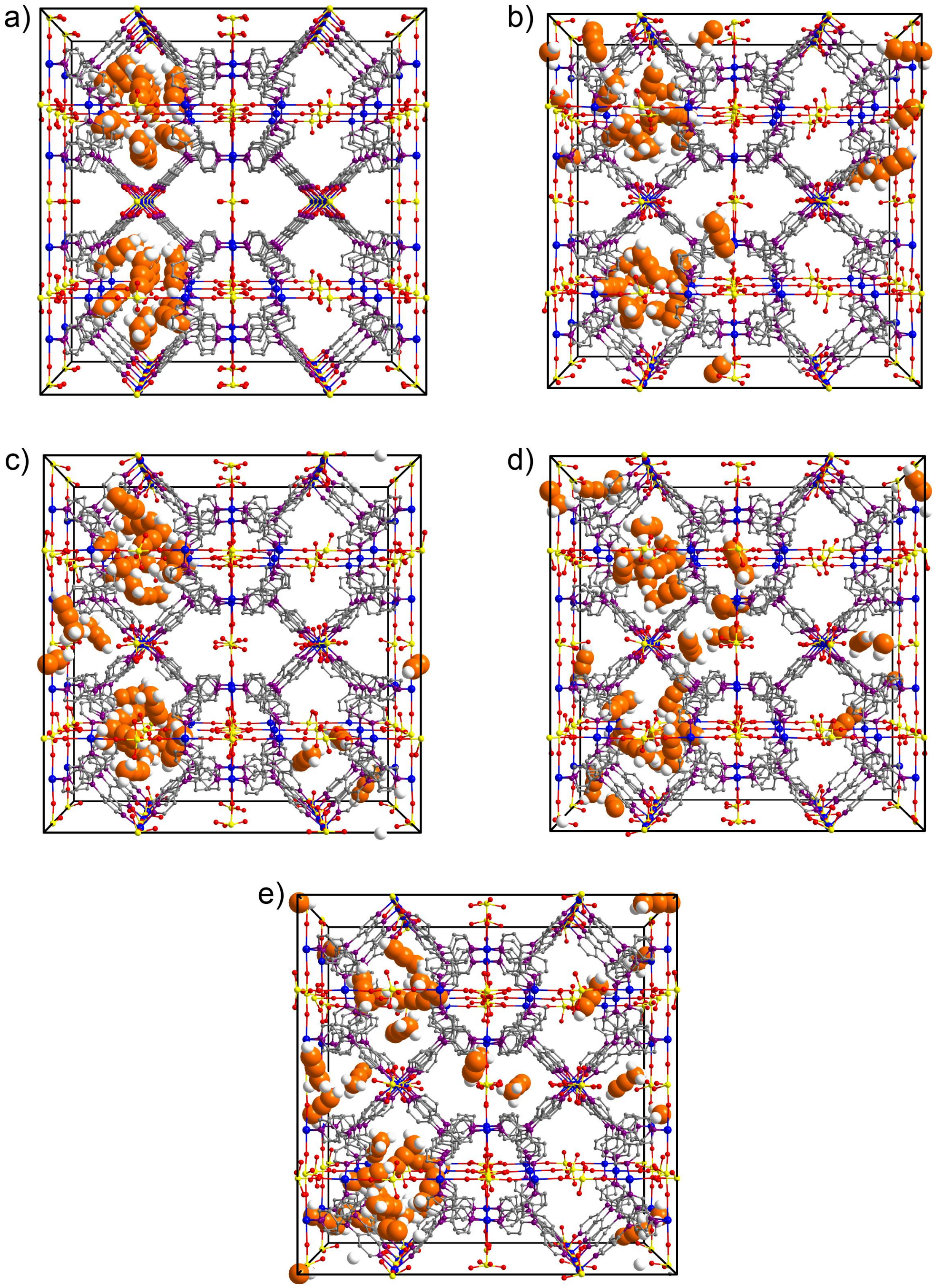

Fig. S73 MD simulations. Snapshots of MD simulation of C_3H_4 molecules in (a) 0, (b) 1250, (c) 2500, (d) 3750 and (e) 5000 ps, under the loading of 7 C_3H_4/cage.

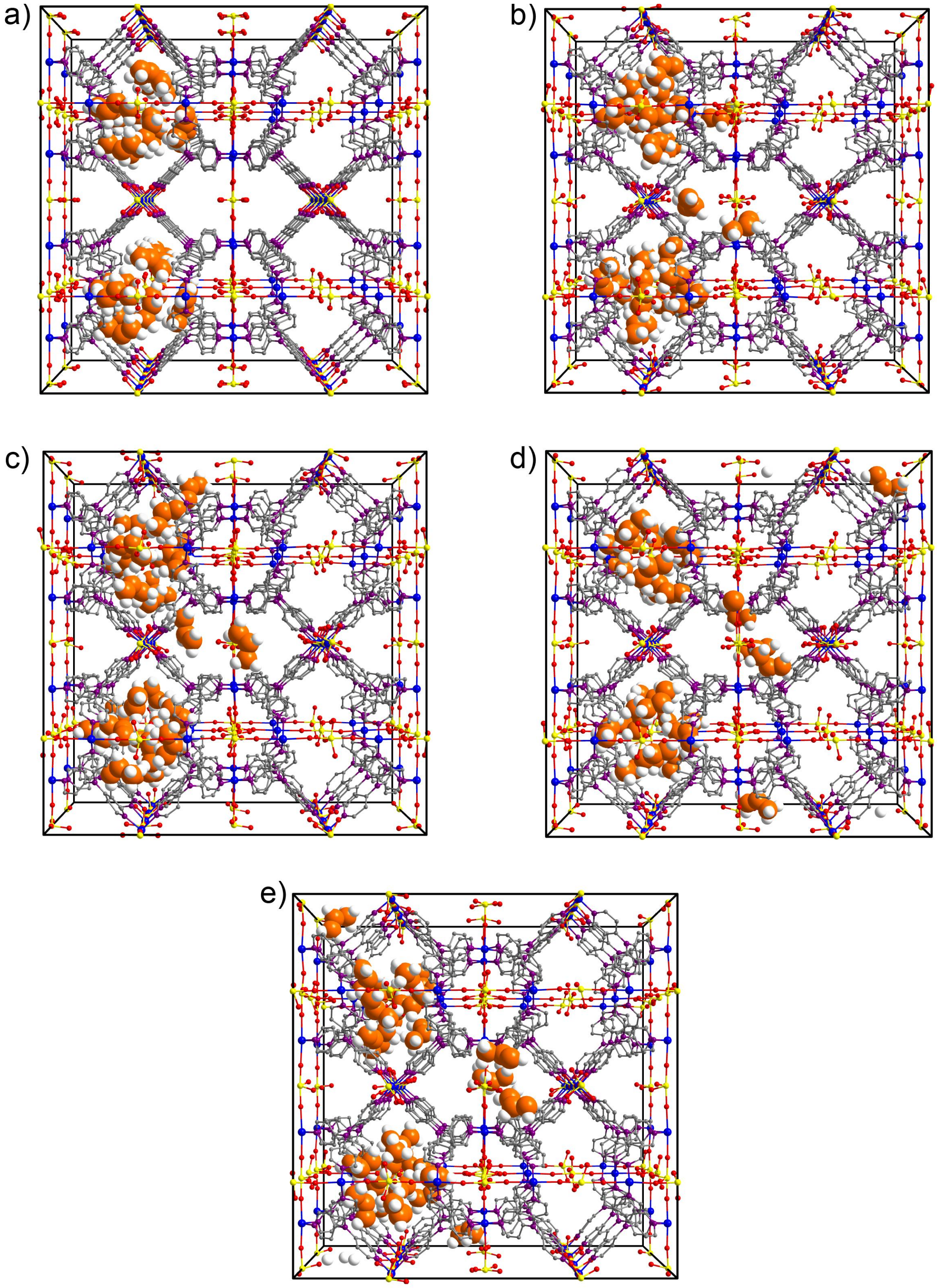

Fig. S74 MD simulations. Snapshots of MD simulation of C_3H_6 molecules in (a) 0, (b) 1250, (c) 2500, (d) 3750 and (e) 5000 ps, under the loading of 7 C_3H_6/cage.

Table S20 The diffusion coefficient of C_3H_4 and C_3H_6 on ZNU-2-Si.

Group	C_3H_4_Slope	C_3H_6_Slope	C_3H_4_diffusion coefficient ($m^2 \cdot s^{-1}$)	C_3H_6_diffusion coefficient ($m^2 \cdot s^{-1}$)
1 molecule	0.02832	4.08E-05	4.72E-11	6.79E-14
2 molecules	0.03908	5.86E-05	6.51333E-11	9.76352E-14
3 molecules	0.02512	9.40E-05	4.18667E-11	1.567E-13
4 molecules	0.02934	2.79E-04	4.89E-11	4.64478E-13
5 molecules	0.05144	0.00284	8.57333E-11	4.73333E-12
6 molecules	0.03942	0.00245	6.57E-11	4.08333E-12
7 molecules	0.04529	0.01499	7.54833E-11	2.49833E-11
8 molecules	0.0449	0.0122	7.48333E-11	2.03333E-11

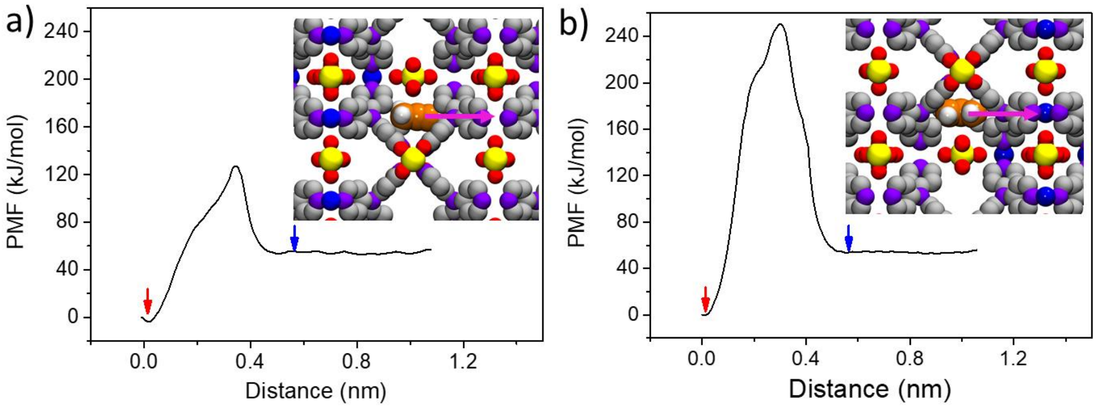

Fig. S75 Potential of mean forces (PMFs) for pulling a C_3H_4 (a) and C_3H_6 (b) from the narrow channel (indicated by red arrow) to cage-like pores (indicated by blue arrow). Insert: Magenta arrows represent the pulling direction of C_3H_4 and C_3H_6 during PMF calculations.

For the above MD simulation, the framework is considered flexible except the Cu atoms. Thus the pyridine ring and SiF_6^{2-} can be rotational and the pore window between the narrow channel and the cage can be expanded to allow guest to transport successfully. These result are consistent with the experiments.

We also tried MD simulation considering the framework is completely rigid. In this case both C_3H_4 and C_3H_6 are very difficult to diffuse from one cage to another due to the limitation of the over-contracted pore window (4.0 Å). Therefore, another method, i.e. comparison of the potential of mean forces, was applied. The free energies of C_3H_4 and C_3H_6 moving from the narrow channel to the cage-like pore were detected by the potential of mean force (PMF) method. The results show that C_3H_4 has lower free energy barrier than C_3H_6, suggesting that C_3H_4 is much easier to transport from channel to cage-like pores than C_3H_6.

VII Breakthrough simulations and experiments

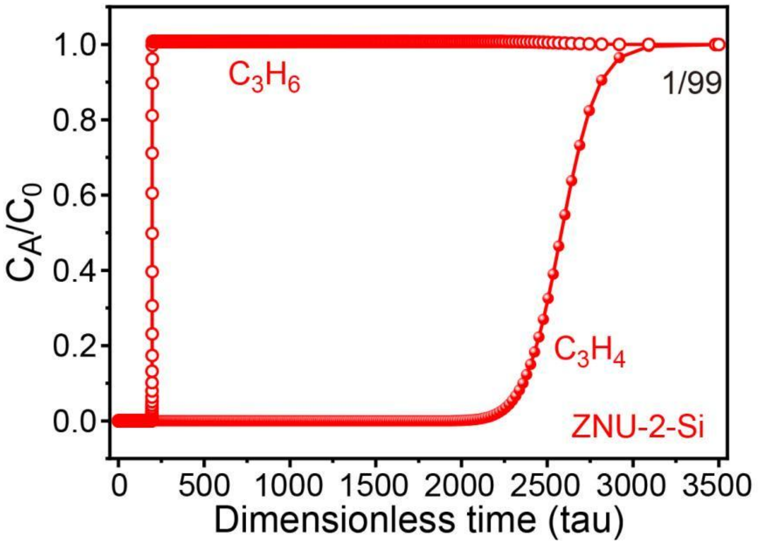

Fig. S76 Simulated breakthrough curves of ZNU-2-Si for C_3H_4/C_3H_6 (1/99) at 298 K.

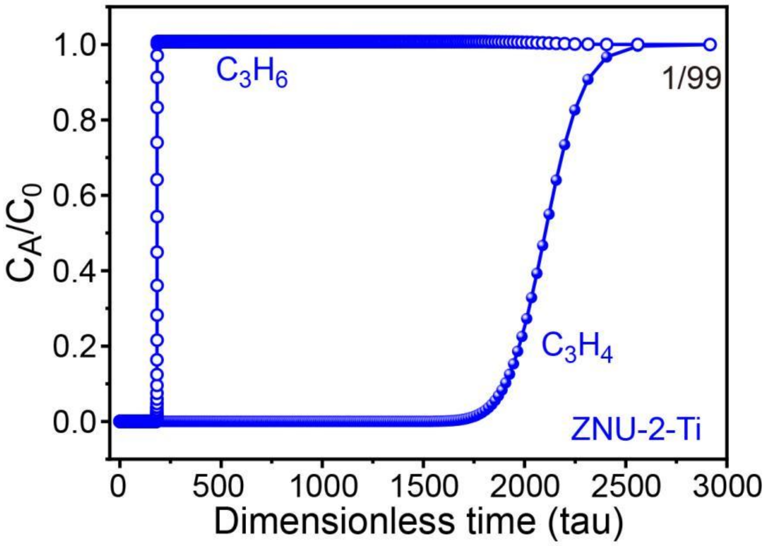

Fig. S77 Simulated breakthrough curves of ZNU-2-Ti for C_3H_4/C_3H_6 (1/99) at 298 K.

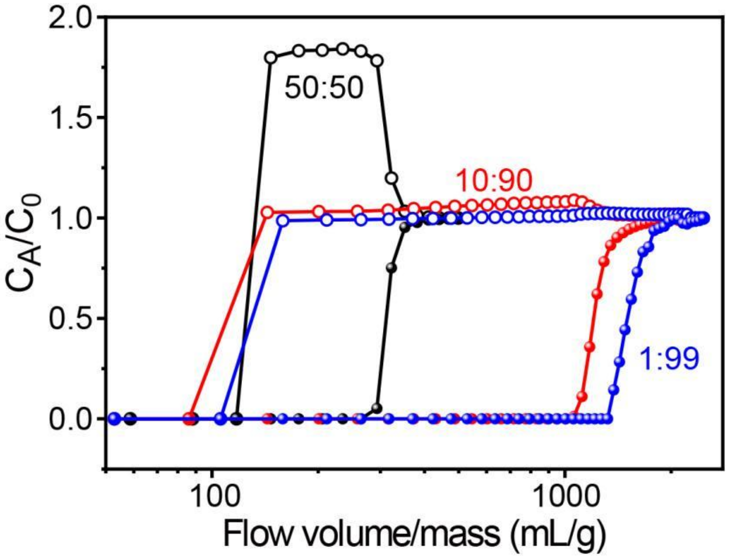

Fig. S78 Comparison of the experimental dynamic breakthrough curves of ZNU-2-Si. Breakthrough for C_3H_4/C_3H_6 with different ratios. Breakthrough conditions: flow rate 4.1 mL/min (50/50), 4.0 mL/min (10/90), 4.3 mL/min (1/99) at 298 K.

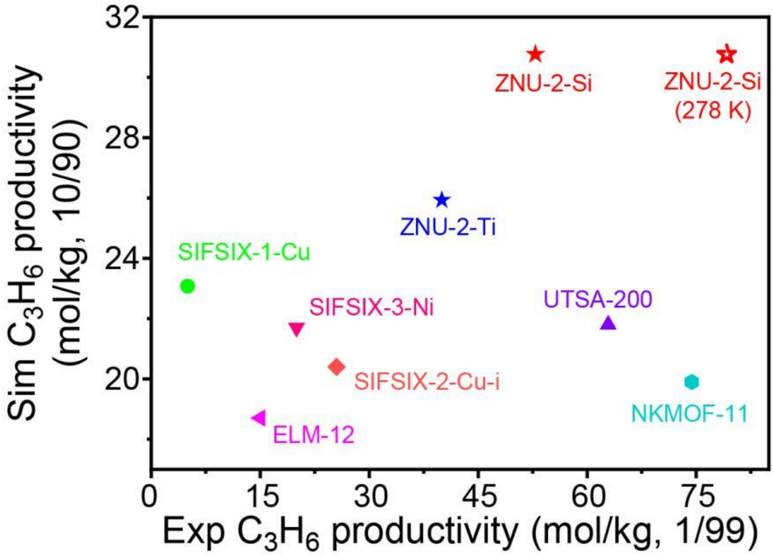

Fig. S79 Comparison of the C_3H_6 productivity from 10/90 (sim) and 1/99 (exp) C_3H_4/C_3H_6.

Table S21 Experimental dynamic C_3H_6 productivity and C_3H_4 uptake for ZNU-2-Si from different gas ratios and under different temperatures.

Conditions	Experimental C_3H_6 productivity (mol/kg)	Experimental C_3H_4 captured amount (mol/kg)
v/v 50/50 298 K	5.38	7.06
v/v 10/90 298 K	37.81	5.54
v/v 1/99 298 K	52.86	0.69
v/v 1/99 278 K	79.20	1.05
v/v 1/99 308 K	47.19	0.30

Table S22 Comparison of the C_3H_6 productivity from 10/90 and 1/99 C_3H_4/ C_3H_6.

	Simulated C_3H_6 productivity from 10/90 mixtures (mol/kg)	Experimental C_3H_6 productivity from 1/99 mixtures (mol/kg)
SIFSIX-1-Cu	23.08	5.0
UTSA-200	21.8	62.9
SIFSIX-3-Ni	21.7	20.0
SIFSIX-2-Cu-i	20.4	25.5
ELM-12	18.7	15.0
NKMOF-11	19.9	74.4
ZNU-2-Ti	25.93 (25.50)[a]	42.0
ZNU-2-Si	30.76 (37.81)[a]	52.9/79.20[b]
[a] Experimental values; [b] 278 K		

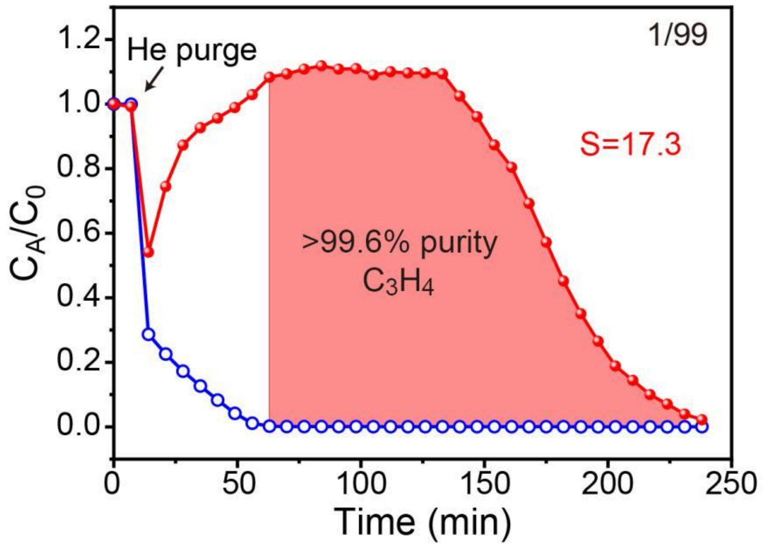

Fig. S80 Experimental dynamic desorption curves of ZNU-2-Si after breakthrough experiment of C_3H_4/C_3H_6 (1/99). Desorption conditions: Ar flow rate 5 mL/min at 348 K.

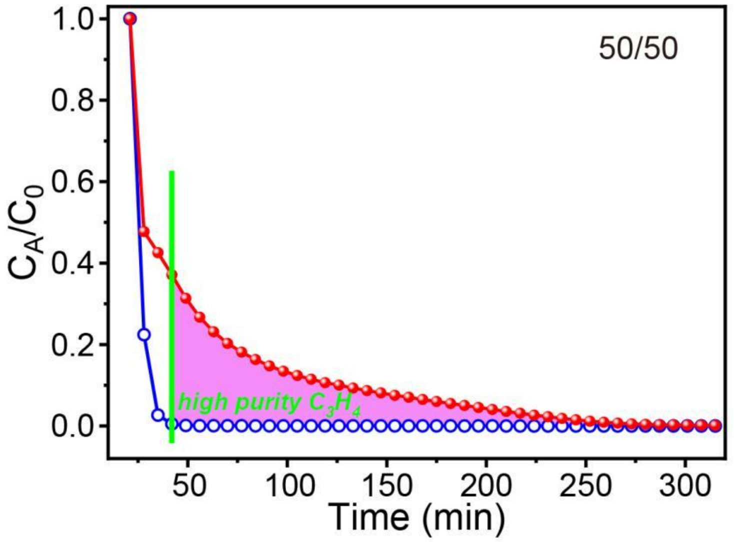

Fig. S81 Experimental dynamic desorption curves of ZNU-2-Si after breakthrough experiment of C_3H_4/C_3H_6 (50/50). Desorption conditions: Ar flow rate 5 mL/min at 348 K. The calculated amount of >99 % purity C_3H_4 (pink area) is 4.7 mmol/g.

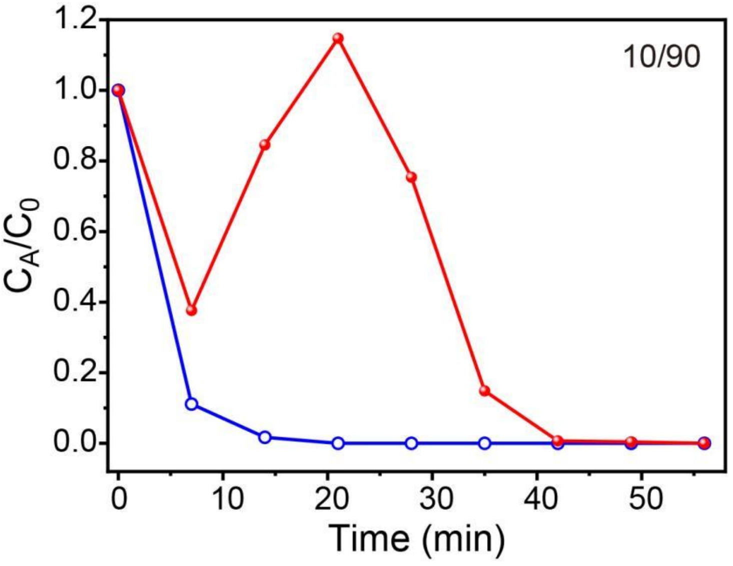

Fig. S82 Experimental dynamic desorption curves of ZNU-2-Si after breakthrough experiment of C_3H_4/C_3H_6 (10/90). Desorption conditions: Ar flow rate 20 mL/min at 393 K.

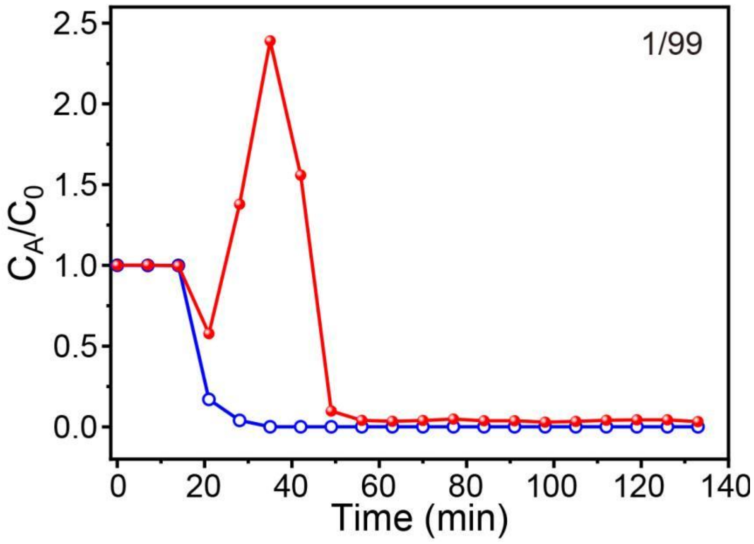

Fig. S83 Experimental dynamic desorption curves of ZNU-2-Si after breakthrough experiment of C_3H_4/C_3H_6 (1/99). Desorption conditions: Ar flow rate 20 mL/min at 393 K.

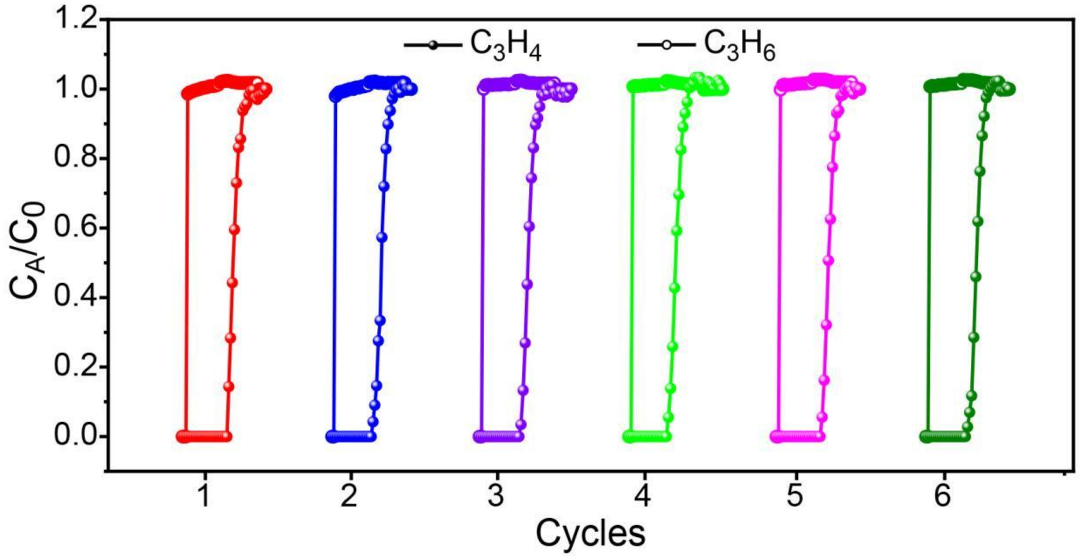

Fig. S84 Six cycles of experimental breakthrough curves of ZNU-2-Si for C_3H_4/C_3H_6 (1/99) at 298 K. (Activation condition of ZNU-2-Si between circles: Ar flow rate 20 mL/min at 393 K).

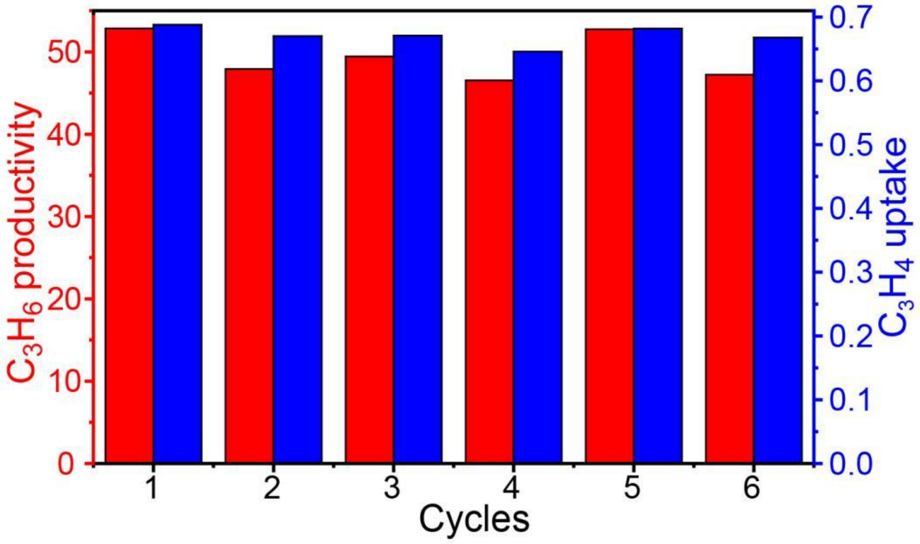

Fig. S85 Comparison of figures for dynamic C_3H_6 production and C_3H_4 uptake of ZNU-2-Si for C_3H_4/C_3H_6 (1/99) at 298 K in 6 cycles (activation condition of ZNU-2-Si between circles: Ar flow rate 20 mL/min at 393 K).

VIII Stability test

Table S23 Comparison of the stability of ZNU-2 with other reported materials in the context of C_3H_4/C_3H_6 separation .

Materials	thermal stability (℃)	air stability	stability in humid air	water stability	stability in acid	stability in base	cycling stability	Ref
ELM-12	295	-	-	√	-	-	√	[1, 17, 18]
ZU-62	230	√	√	√	-	-	√	[14, 19]
SIFSIX-2-Cu-i	170	√	√	×	-	-	√	[20-23]
ZJUT-1	232	√	√	√	-	-	√	[2]
GeFSIX-14-Cu-i	220	-	×	×	-	-	√	[13, 21]
TIFSIX-14-Cu-i	230	-	-	-	-	-	√	[13]
NKMOF-11	-	-	√	√	√	√	√	[3]
JXNU-6	365	-	-	-	-	-	-	[4]
SIFSIX-1-Cu	150	-	×	×	-	-	√	[5, 24, 22]
SIFSIX-3-Ni	264	√	√	×	-	-	√	[5, 22]
SIFSIX-3-Zn	157	-	×	×	-	-	√	[20, 25, 26]

Continued

NbOFFIVE-1-Ni	303	-	√	√	-	-	√	[27-29]
UTSA-200	201	-	×	×	-	-	√	[6, 22, 30]
NKMOF-1-Ni	382	√	√	√	√	√	√	[7, 31]
NKMOF-1-Cu	214	√	√	√	√	√	-	[7, 31]
GeFSIX-dps-Cu	214	√	√	√	-	-	√	[8]
Co-gallate	276	√	-	-	-	-	√	[10]
Mg-gallate	401	√	-	-	-	-	-	[10]
Ni-gallate	290	√	-	-	-	-	-	[10]
Ca-based MOF	520	√	√	√	√	√	-	[11]
Cu-BTC	306	√	×	×	-	-	√	[16, 32-34]
FJI-W1	200	√	√	√	-	-	√	[35]
**ZNU-2-Si**	**250**	√	√	√	√	√	√	**this work**
**ZNU-2-Ti**	**308**	√	√	√	√	√	√	**this work**
**ZNU-2-Nb**	**300**	√	√	√	√	√	√	**this work**

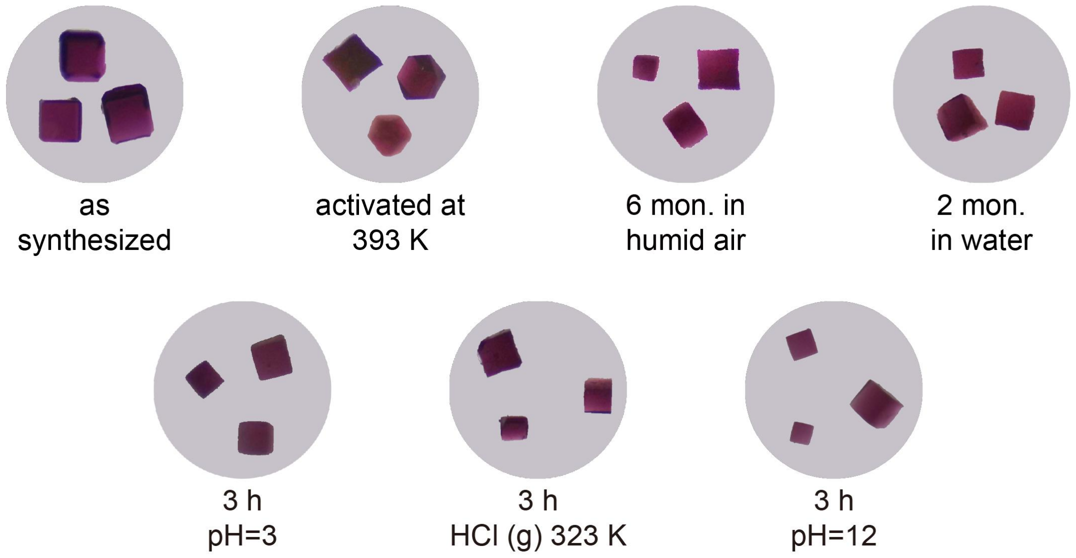

Fig. S86 Photographs of single crystals of ZNU-2-Si after different treatments showing the high stability of ZNU-2-Si after exposure to 393 K heating under vacuum, humid air, water, acid aqueous solution, basic aqueous solution, and acid vapor.

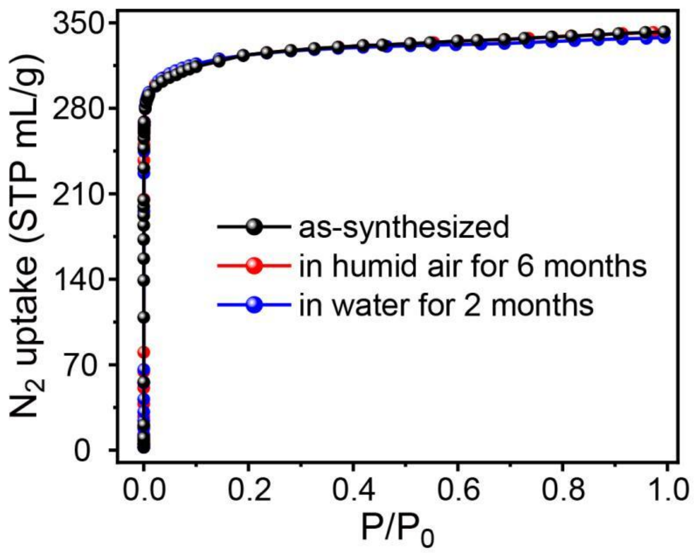

Fig. S87 The adsorption isotherm of N_2 at 77 K on as-synthesized ZNU-2-Si, and ZNU-2-Si after exposure to humid air for 6 months ,soaking in water for 2 months.

Analysis: The overlapping of the N_2 adsorption isotherms on ZNU-2-Si further suggests its good stability.

Table S25 Comparison of the crystallographic parameters of as-synthesized ZNU-2-Si and ZNU-2-Si after multiple sorptions.

Materials	ZNU-2-Si (as-synthesized)	ZNU-2-Si (after multiple sorption experiments)
Cell	a=17.5318(3)	a=17.5267(3)
	b=17.5318(3)	b=17.5267(3)
	c=17.5318(3)	c=17.5267(3)
	α=90	α=90
	β=90	β=90
	γ=90	γ=90
Temperature	293 K	293 K
Volume (Å^3)	5388.6(3)	5383.9(3)
Space group	Pm-3n	Pm-3n
Hall group	-P 4n 2 3	-P 4n 2 3
formula	$C_{20}H_{16}CuF_6N_{5.33}Si$	$C_{20}H_{16}CuF_6N_{5.33}Si$
MW	536.69	536.69
density	0.992	0.993
Z	6	6
R	0.0530(887)	0.0529(904)
wR2	0.1813(1142)	0.1875(1141)
S	1.133	1.131
CCDC Nos.	2190368	2190369

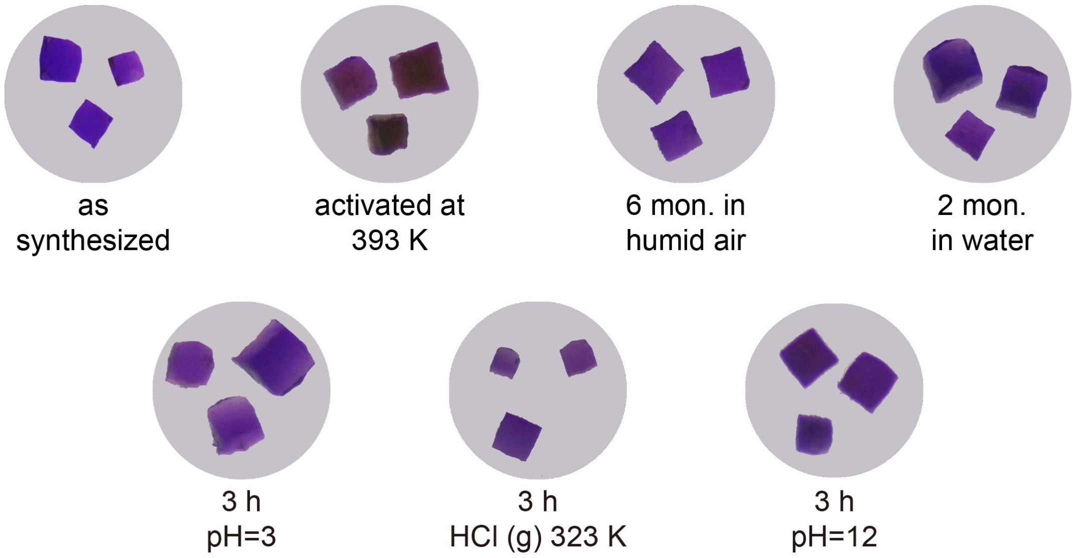

Fig. S88 Photographs of single crystals of ZNU-2-Ti after different treatments showing the high stability of ZNU-2-Ti after exposure to 393 K heating under vacuum, humid air, water, acid aqueous solution, basic aqueous solution, and acid vapor.

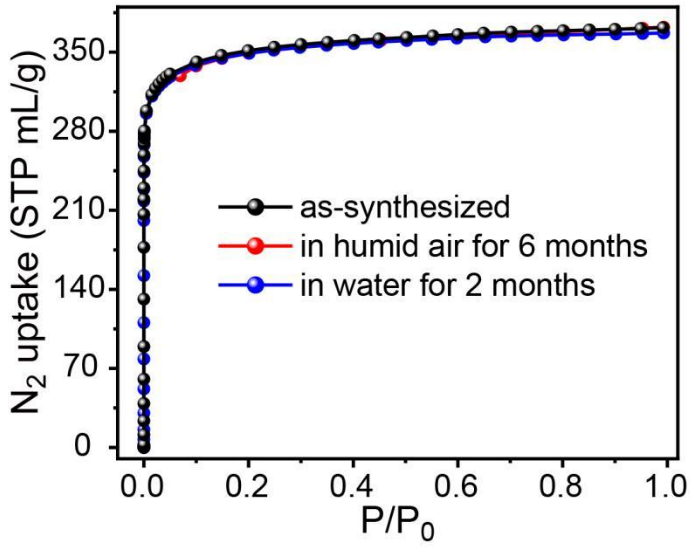

Fig. S89 The adsorption isotherm of N_2 at 77 K on as-synthesized ZNU-2-Ti, and ZNU-2-Ti after exposure to humid air for 6 months ,soaking in water for 2 months。

Analysis: The overlapping of the N_2 adsorption isotherms on ZNU-2-Ti further suggests its good stability.

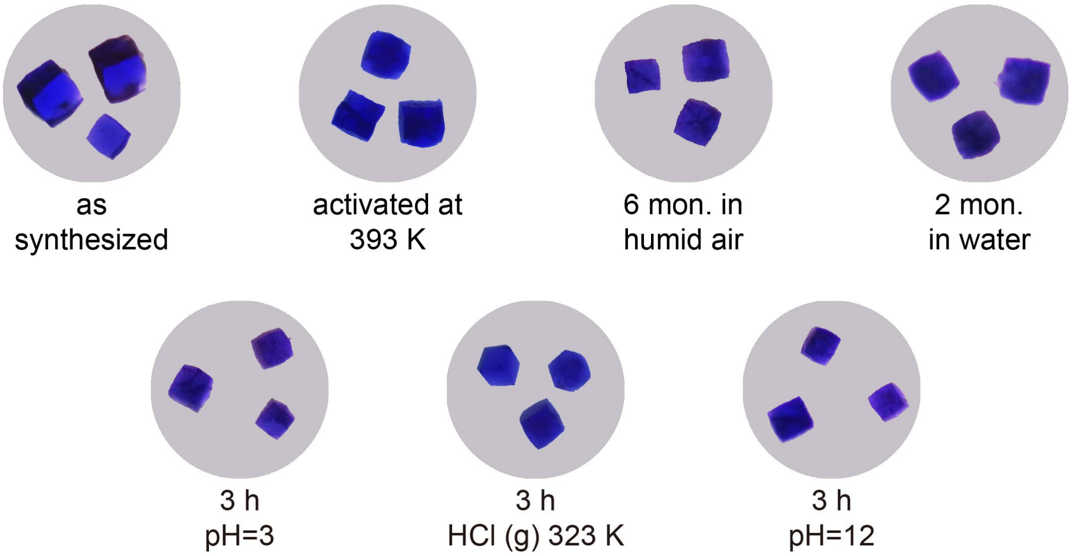

Fig. S90 Photographs of single crystals of ZNU-2-Nb after different treatments showing the high stability of ZNU-2-Nb after exposure to 393 K heating under vacuum, humid air, water, acid aqueous solution, basic aqueous solution, and acid vapor.

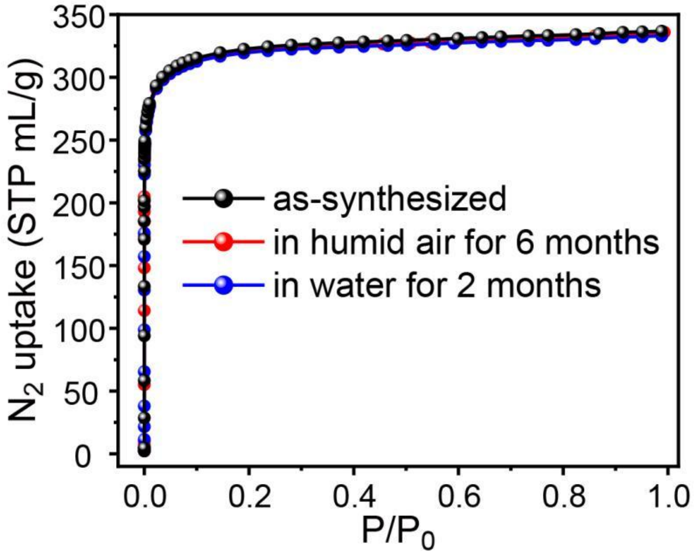

Fig. S91 The adsorption isotherm of N_2 at 77 K on as-synthesized ZNU-2-Nb, and ZNU-2-Nb after exposure to humid air for 6 months ,soaking in water for 2 months.

Analysis: The overlapping of the N_2 adsorption isotherms on the ZNU-2-Nb further suggests its good stability.

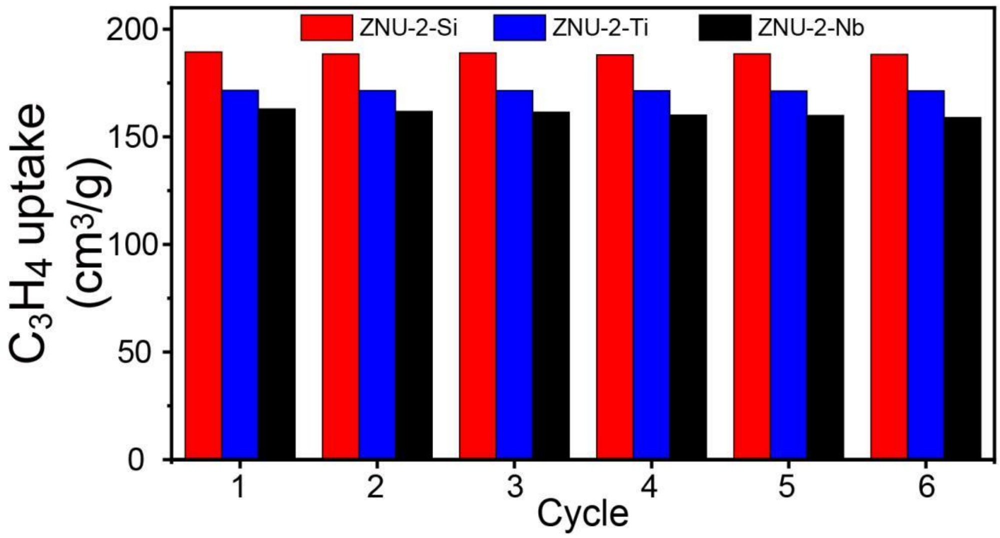

Fig. S92 Comparison of C_3H_4 uptake on the ZNU-2 family at 298 K and 1.0 bar for six cycles.

The retaining of the C_3H_4 uptake on the ZNU-2 family further suggests the good recyclability and stability for applications.

IX References

[1] Li, L. et al. Flexible–Robust Metal–Organic Framework for Efficient Removal of Propyne from Propylene. *J. Am. Chem. Soc.* **139**, 7733-7736 (2017).

[2] Wen, H.-M. et al. Fine-tuning of nano-traps in a stable metal-organic framework for highly efficient removal of propyne from propylene. *J. Mater. Chem. A* **6**, 6931-6937 (2018).

[3] Peng, Y.-L. et al. A robust heterometallic ultramicroporous MOF with ultrahigh selectivity for propyne/propylene separation. *J. Mater. Chem. A* **9**, 2850-2856 (2021)..

[4] Lin, Z. T. et al. Fluorinated Biphenyldicarboxylate-Based Metal-Organic Framework Exhibiting Efficient Propyne/Propylene Separation. *Inorg. Chem.* **59**, 4030-4036 (2020).

[5] Yang, L.et al. A Single-Molecule Propyne Trap: Highly Efficient Removal of Propyne from Propylene with Anion-Pillared Ultramicroporous Materials. *Adv. Mater.* **30**, 1705374 (2018).

[6] Li, L. et al. A Metal-Organic Framework with Suitable Pore Size and Specific Functional Sites for the Removal of Trace Propyne from Propylene. *Angew. Chem. Int. Ed.* **57**, 15183-15188 (2018).

[7] Peng, Y. L. et al. Robust Microporous Metal-Organic Frameworks for Highly Efficient and Simultaneous Removal of Propyne and Propadiene from Propylene. *Angew. Chem. Int. Ed.* **58**, 10209-10214 (2019).

[8] Ke, T. et al. Molecular Sieving of C_2-C_3 Alkene from Alkyne with Tuned Threshold Pressure in Robust Layered Metal-Organic Frameworks. *Angew. Chem. Int. Ed.* **59**, 12725-12730 (2020).

[9] Yu, B. et al. A Solid Transformation into Carboxyl Dimers Based on a Robust Hydrogen-Bonded Organic Framework for Propyne/Propylene Separation. *Angew. Chem. Int. Ed.* **60,** 25942-25948 (2021).

[10] Li, Z. et al. Gallate-Based Metal-Organic Frameworks for Highly Efficient Removal of Trace Propyne from Propylene. *Ind. Eng. Chem. Res.* **59**, 13716-13723 (2020).

[11] Li, L. et al. Calcium-Based Metal-Organic Framework for Simultaneous Capture

of Trace Propyne and Propadiene from Propylene. *ACS Appl. Mater. Interfaces* **12**, 17147-17154 (2020).

[12] Zhang, Z. et al. Fine-Tuning Pore Dimension in Hybrid Ultramicroporous Materials Boosting Simultaneous Trapping of Trace Alkynes from Alkenes. *Small* **16**, 2005360 (2020).

[13] Yang, L. et al. A highly sensitive flexible metal-organic framework sets a new benchmark for separating propyne from propylene. *J. Mater. Chem. A* **6**, 24452-24458 (2018).

[14] Yang, L. et al. An Asymmetric Anion-Pillared Metal-Organic Framework as a Multisite Adsorbent Enables Simultaneous Removal of Propyne and Propadiene from Propylene. *Angew. Chem. Int. Ed.* **57**, 13145-13149 (2018).

[15] Jiang, Y. et al. Comprehensive Pore Tuning in an Ultrastable Fluorinated Anion Cross-Linked Cage-Like MOF for Simultaneous Benchmark Propyne Recovery and Propylene Purification. *Angew. Chem. Int. Ed.* **61**, e202200947 (2022).

[16] Peng, Y. L. et al. Efficient propyne/propadiene separation by microporous crystalline physiadsorbents. *Nat. Commun.* **12**, 5768 (2021).

[17] Kondo, A. et al. Double−Step Gas Sorption of a Two−Dimensional Metal−Organic Framework. *J. Am. Chem. Soc.* **129,** 12362–12363 (2007).

[18] Sotomayor, F. J. Lastoskie, C. M. Carbon dioxide capacity retention on elastic layered metal organic frameworks subjected to hydrothermal cyclin. *Microporous Mesoporous Mater. 304,* 110377 (2020).

[19] Wang, Q. et al. Separation of Xe from Kr with Record Selectivity and Productivity in Anion-Pillared Ultramicroporous Materials by Inverse Size-Sieving. *Angew. Chem. Int. Ed.* **59,** 3423-3428 (2020).

[20] Cui, X. et al. Pore chemistry and size control in hybrid porous materials for acetylene capture from ethylene. *Science* **353**, 141-144 (2016).

[21] Zhang, Z. et al. Sorting of C4 Olefins with Interpenetrated Hybrid Ultramicroporous Materials by Combining Molecular Recognition and Size-Sieving. *Angew. Chem. Int. Ed.* **56**, 16282-16287 (2017).

[22] O'Nolan, D. et al. Water Vapor Sorption in Hybrid Pillared Square Grid

Materials. *J. Am. Chem. Soc.* **139**, 8508-8513 (2017).

[23] Yang, L. et al. A novel interpenetrated anion-pillared porous material with high water tolerance afforded efficient C_2H_2/C_2H_4 separation. *Chem. Commun.* **55**, 5001-5004 (2019).

[24] Noro, S. et al. Framework Engineering by Anions and Porous Functionalities of Cu(II)/4,4'-bpy Coordination Polymers. *J. Am. Chem. Soc.* **124**, 2568-2583 (2002).

[25] Nugent, P. et al. Porous materials with optimal adsorption thermodynamics and kinetics for CO_2 separation. *Nature* **495**, 80-84 (2013).

[26] Meng, S. S. et al. Ultramicroporous metal-organic frameworks for capillary gas chromatographic separation. *J. Chromatogr. A* **1632**, 461604 (2020).

[27] Bhatt, P. M. et al. A Fine-Tuned Fluorinated MOF Addresses the Needs for Trace CO_2 Removal and Air Capture Using Physisorption. *J. Am. Chem. Soc.* **138**, 9301-9307 (2016).

[28] Khraisheh, M. et al.Effective Separation of Prime Olefins from Gas Stream Using Anion Pillared Metal Organic Frameworks: Ideal Adsorbed Solution Theory Studies, Cyclic Application and Stability. *Catalysts* **11**, 510 (2021).

[29] Chen, K. et al. Enhanced CO_2/CH_4 separation performance of mixed-matrix membranes through dispersion of sorption-selective MOF nanocrystals. *J. Membr. Sci.* **563**, 360-370 (2018).

[30] Li, B. et al. An Ideal Molecular Sieve for Acetylene Removal from Ethylene with Record Selectivity and Productivity. *Adv. Mater.* **29**, 1704210 (2017).

[31] Peng, Y. L. et al. Robust Ultramicroporous Metal–Organic Frameworks with Benchmark Affinity for Acetylene. *Angew. Chem. Int. Ed.* **57**, 10971-10975 (2018).

[32] Xu, S. et al. Methyl-Shield Cu-BTC with High Water Stability through One-Step Synthesis and In Situ Functionalization *Ind. Eng. Chem. Res.* **59**, 12451-12457 (2020).

[33] Sun, Y. et al. Room-Temperature Synthesis of $Pyr_{1/3}$@Cu–BTC with Enhanced Stability and Its Excellent Performance for Separation of Propylene/Propane. *Ind. Eng. Chem. Res.* **59**, 6202-6209 (2020).

[34] Kumar, A. et al. Direct Air Capture of CO_2 by Physisorbent Materials. *Angew.*

Chem. Int. Ed. **54**, 14372-14377 (2015).

[35] Zou, S. et al. Stable Fluorinated Hybrid Microporous Material for the Efficient Separation of C_2-C_3 Alkyne/Alkene Mixtures. *Inorg. Chem.* **61**, 7530-7536 (2022).
